# Two legume fatty acid amide hydrolase isoforms with distinct preferences for microbial- and plant-derived acylamides

**DOI:** 10.1038/s41598-023-34754-z

**Published:** 2023-05-09

**Authors:** Omar Arias-Gaguancela, Emily Herrell, Mina Aziz, Kent D. Chapman

**Affiliations:** BioDiscovery Institute, Department of Biological Sciences, University of North Texas, Denton, TX USA

**Keywords:** Enzymes, Biochemistry, Plant sciences

## Abstract

Fatty acid amide hydrolase (FAAH) is a widely conserved amidase in eukaryotes, perhaps best known for inactivating *N*-acylethanolamine lipid mediators. However, FAAH enzymes hydrolyze a wide range of acylamide substrates. Analysis of FAAHs from multiple angiosperm species revealed two conserved phylogenetic groups that differed in key conserved residues in the substrate binding pocket. While the foundation group of plant FAAHs, designated FAAH1, has been studied at the structural and functional level in *Arabidopsis thaliana*, nothing is known about FAAH2 members. Here, we combined computational and biochemical approaches to compare the structural and enzymatic properties of two FAAH isoforms in the legume *Medicago truncatula* designated MtFAAH1 and MtFAAH2a. Differences in structural and physicochemical properties of the substrate binding pockets, predicted from homology modeling, molecular docking, and molecular dynamic simulation experiments, suggested that these two FAAH isoforms would exhibit differences in their amidohydrolase activity profiles. Indeed, kinetic studies of purified, recombinant MtFAAHs indicated a reciprocal preference for acylamide substrates with MtFAAH1 more efficiently utilizing long-chain acylamides, and MtFAAH2a more efficiently hydrolyzing short-chain and aromatic acylamides. This first report of the enzymatic behavior of two phylogenetically distinct plant FAAHs will provide a foundation for further investigations regarding FAAH isoforms in legumes and other plant species.

## Introduction

Fatty acid amide hydrolase (FAAH) is a conserved amidase that hydrolyzes lipophilic *N*-acylethanolamines (NAEs) into ethanolamine and free fatty acid^1–4^. For example; the rat, mouse and human FAAHs hydrolyze endogenous anandamide (NAE20:4) into arachidonic acid and ethanolamine^5,6^. In mammals, this process is linked to a variety of neurological and physiological processes ranging from pain perception to diet regulation^7,8^. In another example, the recombinant FAAH derived from the plant moss, *Physcomitrella patens*, was also able to hydrolyze NAE20:4 into corresponding products, which appeared to be associated with growth and development of the moss species^9^. Elsewhere, *C. elegans* was shown to possess a FAAH enzyme which metabolized NAE20:4 in support of regulation of longevity^10^. These, and numerous other reports have demonstrated a broadly-conserved FAAH machinery across multiple species for NAE regulation in various biological processes.

In the model plant, *Arabidopsis thaliana*, in vitro and in vivo studies showed that *A. thaliana* FAAH (AtFAAH) can cleave the amide bond of endogenous NAEs, including saturated (e.g. NAE12:0), monosaturated (e.g. NAE18:1), and polyunsaturated (e.g. NAE18:3) species, as well as 9-LOX derived NAE oxylipins (9-hydroxy linoleoylethanolamide; NAE-9-HOD)^1,11–13^. Regulation of NAE contents by FAAH appears to be associated with a variety of biological processes^14^. For example, in Arabidopsis, *AtFAAH* overexpressing lines had lower amounts of endogenous NAEs, and exhibited enhanced differences in seedling growth^15^, flowering time^16^ and innate immunity^17^, compared to wild-type plants. By contrast, Arabidopsis *faah*-knockouts had elevated endogenous NAE contents, and were hypersensitive to growth inhibition induced by external applications with NAEs (e.g. NAE12:0 or NAE18:2)^15,18,19^, alkamides^20,21^, *N*-acyl homoserine lactones (AHLs)^22^, or the phytohormone ABA^13^. In another report, upland cotton (*Gossypium hirsutum* L.) seedlings with ectopic overexpression of *AtFAAH* displayed insensitivity to NAE12:0, or the NAE18:2-derived hydroxide (NAE-9-HOD), thus resembling the outcomes found in Arabidopsis^23^. These reports suggest that regulation of *N*-acylethanolamide content by FAAH may influence a number of physiological processes in plants.

*N*-*A*cyl-ʟ-homoserine lactones (AHLs) or *N*-aryl-ʟ-homoserine lactones (aryl-HLs) are bacteria-derived lipids that are utilized in cell-to-cell communication (quorum sensing; QS) processes (e.g. biofilm production) or interactions with a host (e.g. recognition of symbiotic or pathogenic bacteria)^24–26^. Due to the structural similarities between NAEs and these QS molecules (polar head group with an amide-linked acyl tail), it was hypothesized that plant FAAHs could hydrolyze AHLs and aryl-HLs^22^. Further experiments revealed that recombinant AtFAAH hydrolyzes long acyl chain AHLs such as OdDHL (N-(3-oxododecanoyl)-ʟ-homoserine lactone) or OtDHL (N-(3-oxotetradecanoyl)-ʟ-homoserine lactone) better than AHLs with shorter chains such as OHHL (N-(3-Oxohexanoyl)-ʟ-homoserine lactone)^22^, thus indicating that AtFAAH activity can be influenced by the length/nature of the acyl chain in the substrate. In the same experiment, the aryl-HL, *p*-coumaryl-HL was a poor substrate for AtFAAH^22^. Also, AHLs were shown to trigger a “biphasic” growth response in *A. thaliana* seedlings. In their experiments, wild-type Arabidopsis seedlings fed with AHLs displayed enhanced or reduced growth phenotypes at low (e.g. 0.1 µM) or elevated AHL (e.g. 100 µM) concentrations, respectively^22^. Then, side-by-side comparisons with Arabidopsis *faah*-knockouts, revealed that such sensitivity can be modulated in a FAAH-dependent manner, that is, hydrolysis of AHLs by FAAH was required for modulation of the growth signal^22^.

Alkamides are fatty acid amides of fungal- or plant-origin^21,27,28^ and constitute another group of NAE-like molecules that have been associated with FAAH-mediated hydrolysis^20^. In wild-type Arabidopsis, exogenous applications of the alkamide, affinin (*N*-isobutyl head group and acyl chain) resulted in a “biphasic” effect that resembled that of AHLs (see above). Indeed, Arabidopsis wild-type seedlings fed with affinin at concentrations below 28 µM had primary roots that were longer than controls^21^. Conversely, affinin at concentrations above 28 µM led to markedly impaired root development^21^. In another study, Arabidopsis *FAAH* overexpressors or *faah*-knockouts demonstrated that alkamide susceptibility was modulated via FAAH-hydrolysis^20^. Interestingly, the in vitro AtFAAH activity towards affinin or other 12 alkamides of various acyl chain lengths and alkyl head groups was far more inferior compared to that of AtFAAH hydrolysis of NAEs^20^. Nonetheless, these reports support a connection between FAAH-hydrolysis of alkamides and plant growth effects.

FAAH is characterized by the presence of a conserved Ser-Ser-Lys catalytic triad^1,29,30^. The *N*-acyl amides that reach the active site at the acyl binding channel (ABC) form a covalent intermediate between the substrate and the enzyme. This is achieved by activation of the catalytic serine (nucleophile) which attacks the carbon of the carbonyl group in the substrate^31^. Mammalian and plant FAAHs appeared to have distinct mechanisms for substrate binding. For example, AtFAAH is proposed to undergo conformational changes that lead to the movement of a helix region (residues 531–537) and the closure of its membrane access channel (MAC), by a process termed “squeeze and lock”^1^. By contrast, in the rat FAAH, the ligand-enzyme complex is secured by a “dynamic paddle” which closes its narrower MAC and “traps” the substrate in the active site. In both cases, following FAAH catalysis, ethanolamine is released into the cytosol via the cytosolic access channel (CAC) whereas the fatty acid product is released back into the hydrophobic environment of the membrane bilayer^1,29^. The resolved crystal structures of both plant and mammalian FAAHs have provided insights into key structural and functional components that could be used as reference for FAAHs from other organisms.

Recently, the comparison of 88 FAAH amino acid sequences from diverse angiosperm species revealed two major phylogenetic groups, FAAH1 and FAAH2^32^. Plant species in the Brassica family such as *A. thaliana* or *Camelina sativa* exhibited only one FAAH isoform (FAAH1) whereas all dicot and monocot species, including *Amborella trichopoda* (the “angiosperm ancestral species”) had both FAAH groups, and many having more than one member^32^. For example, tomato (*Solanum tuberosum*) has three FAAH1 isoforms and one FAAH2, whereas rice (*Oryza sativa*) has one FAAH1 and two FAAH2 isoforms^32^. Analysis of multiple sequence alignments exhibited residue changes in regions corresponding to the substrate binding pocket of FAAH1 and FAAH2 isoforms. Homology modeling of soybean (*Glycine max*) FAAHs revealed additional structural and chemical differences between the soybean FAAH1 and FAAH2^32^. These differences were hypothesized to influence the substrate selectivity of FAAH1 and FAAH2 enzymes, and further suggest that FAAHs may have evolved distinct FAAH machineries to modulate an expanded repertoire of lipophilic signaling lipids^32,33^. Although conceptually this notion appears plausible, thus far, experimental evidence to support this hypothesis is lacking, largely because there is not biochemical information related to the FAAH2 group of enzymes. Here, we address this gap in knowledge by combining computational and biochemical approaches to study and characterize two FAAHs from the legume *Medicago truncatula*, MtFAAH1 and MtFAAH2a, and provide evidence that these two isoforms have reciprocal substrate preferences.

## Results

### Group I and II FAAHs of the legume *M. truncatula*

**Figure 1:**
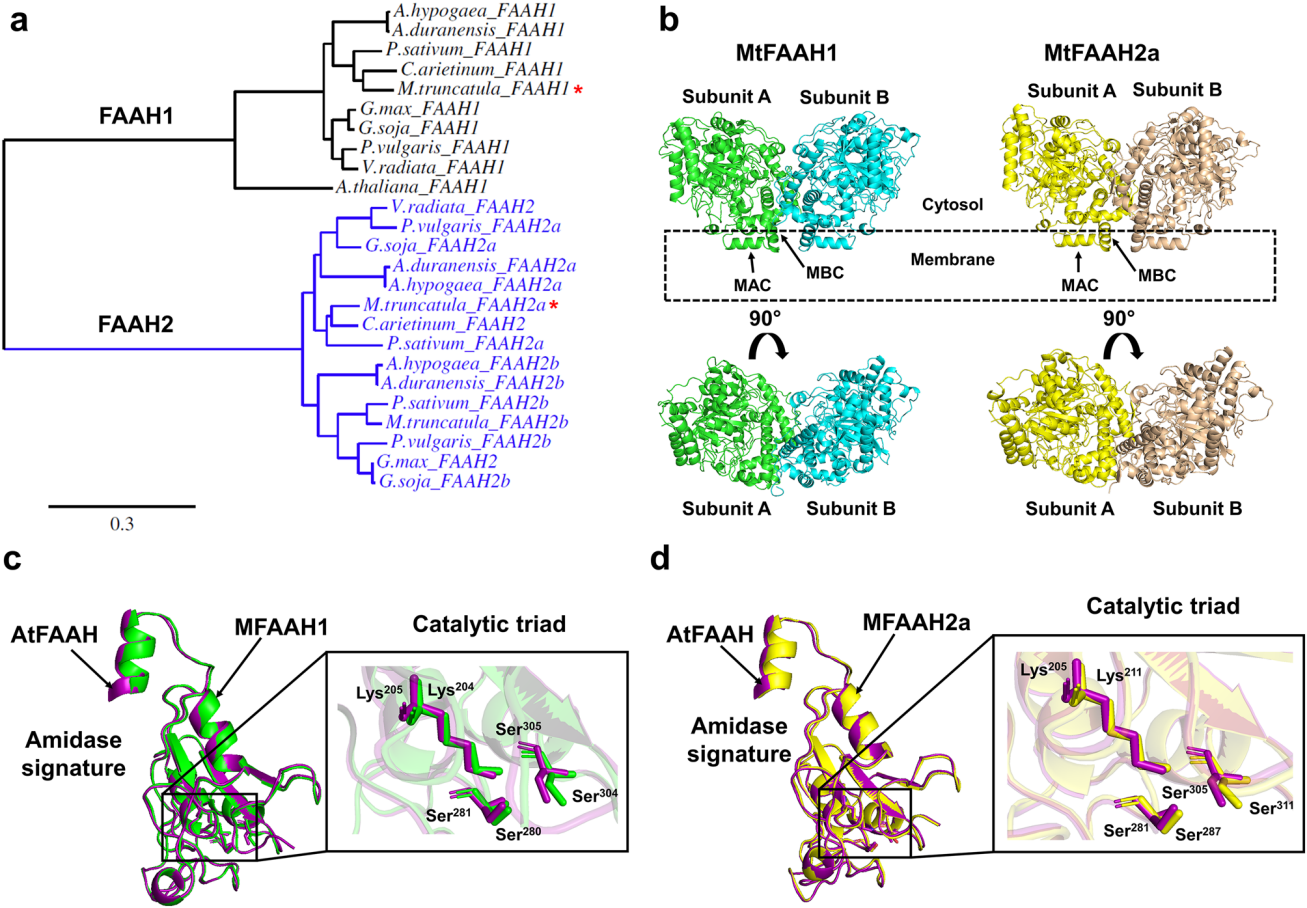
Analysis of sequences and homology models of *Medicago truncatula* FAAHs, namely, MtFAAH1 and MtFAAH2a. (**a**) Phylogenetic analysis of amino acid sequences of FAAHs from 10 different legume species. *A. thaliana* FAAH (AtFAAH) sequence was also included in the analysis, as FAAH1 control. Asterisks denote the MtFAAH sequences that were further investigated in this study. (**b**) Homology models of MtFAAH1 and MtFAAH2a (upper panel). Membrane access channel (MAC) and membrane binding cap (MBC) are labelled in the structures. A 90° view of the same models are included (lower panel) for visualization purposes. The amidase signature (AS) domain of AtFAAH (purple) was superimposed with the (**c**) AS of MtFAAH1 (green) or (**d**) the AS of MtFAAH2a (yellow). A close-up view at their catalytic triad residues is presented for both (**c**) and (**d**).

To examine the FAAH diversity within legumes, we compared FAAH amino acid sequences of ten selected species using AtFAAH (a FAAH1 enzyme) as reference (Fig. 1a; Supplementary Table S1). Data showed that these legume FAAHs clustered in two major groups, designated as FAAH1 and FAAH2 (Fig. 1a). Only one FAAH1 isoform was identified in each legume species. Notably, FAAH2 formed two subgroups in most species, FAAH2a and FAAH2b. With the exception of *G. max* and *Vigna radiata*, the rest of legumes (including *M. truncatula*) had two FAAH2 isoforms. Compared to the founding FAAH1 member, AtFAAH, the percentages of amino acid sequence similarity for MtFAAH1, MtFAAH2a, or MtFAAH2b were approximately 66%, 44%, and 44%, respectively (Supplementary Fig. S1; Supplementary Table S2).

Inspection of the transcript expression profiles of *MtFAAH1*, *MtFAAH2a* and *MtFAAH2b* in the *Medicago truncatula* Gene Expression Atlas “MtExpress”^34^ showed that *MtFAAH1* or *MtFAAH2a* were highly expressed in different tissues, temperature conditions, time points, abiotic stresses (e.g. N- starvation) and biotic stresses (e.g. exposure to the arbuscular mycorrhizal fungus *Rhizophagus irregularis*) whereas in the same conditions *MtFAAH2b* was expressed at a much lower level (Supplementary Fig. S2). Based on these data, we selected MtFAAH1 and MtFAAH2a for further analysis with both computational and biochemical approaches.

Homology models of MtFAAH1 and MtFAAH2a were predicted based on the crystal structure of AtFAAH (PBD: 6DHV) for comparisons of their structural features (Fig. 1b). The quality of the MtFAAH1 and MtFAAH2a models was supported with GMQE (Global Model Quality Estimate) scores (0.90 for MtFAAH1 and 0.80 for MtFAAH2a), QMEANDisCo (Qmean consensus-based distance constraint) scores (0.88 for MtFAAH1 and 0.79 for MtFAAH2a), and Ramachandran scores (Ramachandran favored; 95.59% for MtFAAH1 and 94.31% for MtFAAH2a) (Supplementary Figs. S3, S4; Supplementary Table S3, S4). Both models are shown as homodimers (Fig. 1b) with similar overall predicted content and organization of alpha helices, beta-sheets, turns, and unstructured coils (Supplementary Fig. S5). Both MtFAAH1 and MtFAAH2a have conserved membrane binding caps (MBCs) and membrane access channels (MACs) at their N-termini (Fig. 1b, Supplementary Fig. S6), which are predicted to anchor FAAH to the membrane^1^. Like in AtFAAH crystal structure, the MBCs of both MtFAAHs are characterized by a prevalence of hydrophobic residues (Supplementary Fig. S6). As expected, the amidase signature (AS) domain of AtFAAH is characterized by the presence of a Ser-Ser-Lys catalytic triad as in other FAAH family members, and these residues are conserved here in MtFAAH1 and MtFAAH2a (Fig. 1c,d). Altogether, these data highlight conservation of predicted membrane association and active site residues for MtFAAH1 and MtFAAH2a.

### Residue differences between the substrate binding pockets of MtFAAH1 and MtFAAH2a

**Figure 2:**
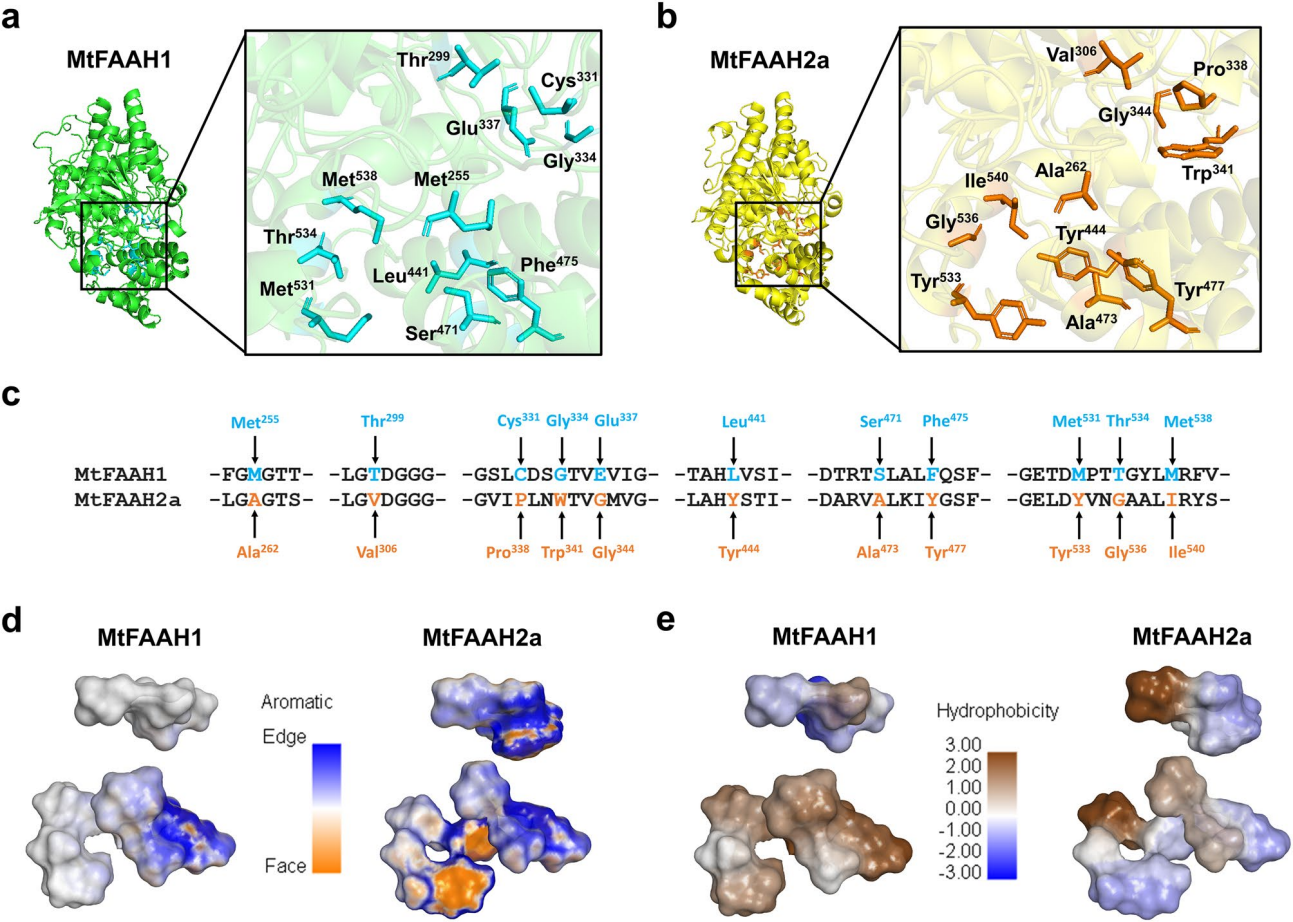
Substrate binding pockets (SBPs) of MtFAAH1 and MtFAAH2a reveal different structural and physicochemical profiles. Residues predicted to be different in the SBPs of (**a**) MtFAAH1 (cyan color sticks) or (**b**) MtFAAH2a (orange color sticks) are displayed. The complete list of residues predicted to form the SBPs of both MtFAAHs can be found in Supplementary Table S3. (**c**) Partial alignment between MtFAAH1 and MtFAAH2a amino acid sequences. Arrows pointing at residues in cyan or orange fonts represent distinct residues in the SBP of MtFAAH1 or MtFAAH2a, respectively. (**d**) Aromatic surfaces for the residues highlighted in (**a**), (**b**), and (**c**) reveal different aromatic profiles. Aromatic scale describes the edge (blue)-to-face (orange) conformations of residues with an aromatic side-chain. (**e**) Hydrophobicity surface profiles for the residues highlighted in (**a**), (**b**), and (**c**). Hydrophobicity scale ranges from 3.00 for highest (brown) or − 3.00 for lowest (blue) hydrophobic regions.

Previously, a report highlighted several key residue differences that altered the predicted shape and properties of the substrate binding pockets (SBPs) of soybean FAAH1 and FAAH2^32^. In order to corroborate that such findings are conserved in a different legume species, we analyzed the SBPs of *M. truncatula* FAAHs, MtFAAH1 and MtFAAH2a. Data revealed multiple residue differences especially within the cytosolic access (CAC) and acyl binding (ABC) channels (Fig. 2; Supplementary Table S5). The CAC of MtFAAH1 has several polar (e.g. Thr^299^, Glu^337^) whereas these residues in the CAC of MtFAAH2a are nonpolar amino acids (e.g. Val^306^, Trp^341^) (Fig. 2a–e). Further, MtFAAH1 has positioned the less bulky residue, Gly^334^ in its CAC, this appeared to provide a more opened cavity compared to that of its FAAH2 counterpart (Fig. 2a–e). Conversely, MtFAAH2a has the large aromatic residue, Trp^341^ in its CAC, and this results in a more restrictive CAC (Fig. 2a–e). The ABC of MtFAAH1 is characterized by nonpolar/hydrophobic residues such as Leu^441^, Phe^475^, Met^531^ whereas in MtFAAH2a those residues have been replaced by three tyrosine residues (Tyr^444^, Tyr^477^, and Tyr^533^) which provide more neutral, polar and aromatic environments (Fig. 2a–e). Further, the tyrosine residues in MtFAAH2a resulted in a more closed, shorter ABC (Fig. 2a–e). By contrast, the residues in MtFAAH1 yielded a more opened ABC, as shown in their aromatic and hydrophobic surface profiles (Fig. 2d,e) more similar to the ACB of AtFAAH1^1^. Notably, several of the residues found in the SBPs of MtFAAH1 or MtFAAH2 are conserved among the FAAH1 or FAAH2 groups of multiple legume species (Supplementary Fig. S7). Together, these data suggest that MtFAAH1 and MtFAAH2a have distinct structural and physiochemical properties in their SBPs. Therefore, these different SBPs are likely to accommodate different acylamide structures.

### Molecular docking of MtFAAH1 and MtFAAH2a

**Figure 3:**
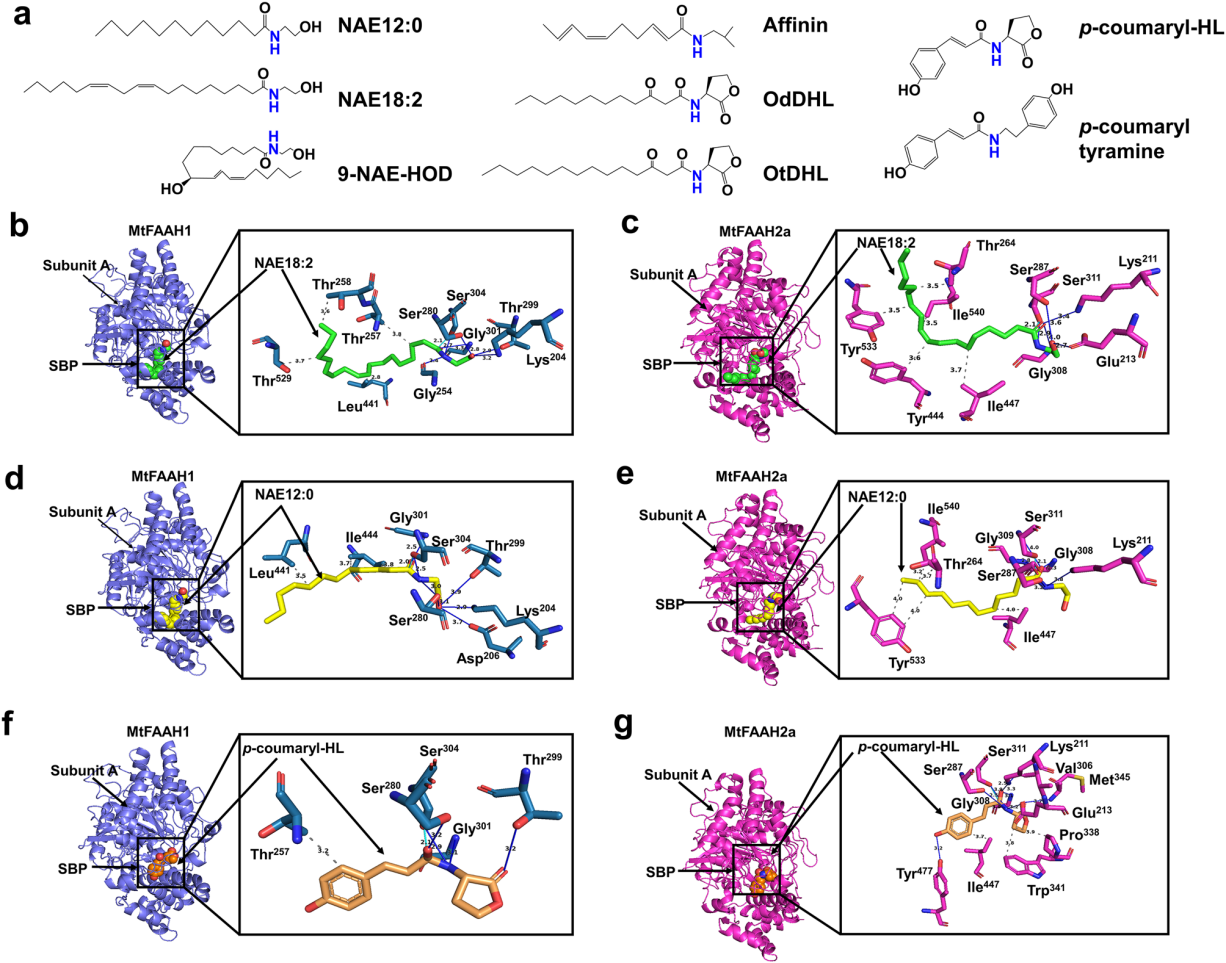
Molecular docking of subunit A of MtFAAH1 or MtFAAH2a with different *N*-acyl amides. The 2D structures of eight potential ligands for MtFAAHs were drawn using ChemDraw (Molecular Editor) software (**a**). Out of the eight ligand candidates, we selected three for docking experiments, namely, NAE18:2, NAE12:0 and *p*-coumaryl-HL. Apo MtFAAH1 and MtFAAH2a were subjected to molecular dynamic simulations (MDS) (during 100 ns) prior to all docking experiments. Wider and close-up views of NAE18:2 (**b**, **c**), NAE12:0 (**d**, **e**), or *p*-coumaryl-HL (**f**, **g**) docked in MtFAAH1 or MtFAAH2a. Covalent bonds are represented as cyan lines between the side-chain oxygens of the catalytic residues of MtFAAH1 (Ser^304^) or MtFAAH2a (Ser^311^) and the carbon of the carbonyl group in the substrates. Hydrogen bonds are displayed as blue lines whereas hydrophobic (van der Waals) interactions are denoted as gray-dashed lines between the interacting atoms. Numbers displayed in the bonds, represent distances (Å) between the contact points. Abbreviations: Substrate binding pocket (SBP).

To test how the differences in the SBPs of MtFAAH1 and MtFAAH2a could alter substrate binding and accommodation, we carried out molecular docking experiments (Fig. 3). To geometrically stabilize both MtFAAH1 and MtFAAH2a prior to the docking experiments, we conducted molecular dynamic simulations (MDS) of the apo forms of MtFAAH1 and MtFAAH2a for 100 ns. The resulting MD trajectories were studied via Root-Mean-Square Deviation (RMSD) and Root-Mean-Square-Fluctuation (RMSF) calculations. Both MtFAAHs appeared stable throughout the entire simulation with RMSD values of approximately 1.5–1.7 Å for MtFAAH1 and 1.8–2.2 Å for MtFAAH2a (Supplementary Fig. S8). The RMSF values showed similar fluctuation profiles for both MtFAAHs, except for a sequence region in MtFAAH2a comprised by residues 366–387 where a higher fluctuation was observed when compared to MtFAAH1 (Supplementary Fig. S8).

For docking studies three structurally different acylamides (Fig. 3a) were compared– NAE18:2, NAE12:0, or *p*-coumaryl-HL. The MD stabilized MtFAAH1 or MtFAAH2a homology models were docked with these three ligands (Fig. 3b–g). Docking revealed the potential for covalent bonds between the side-chain oxygen (Oγ) of Ser^304^ or Ser^311^ and the carbon of the carbonyl group of the substrates with bond distances that ranged from 2.1 to 2.6 Å. The polar ethanolamine head groups of NAEs (NAE18:2 or NAE12:0) were supported by multiple hydrogen-bonds in the SBPs of both MtFAAH1 (Fig. 3b,d) and MtFAAH2a (Fig. 3c,e); this involves residues such as Gly^301^, Gly^254^, Thr^299^ and Asp^206^ in MtFAAH1, and residues such as Gly^308^ and Gly^309^ in MtFAAH2a. Inspection of MtFAAHs bound to *p*-coumaryl-HL revealed some interesting similarities and differences. Both MtFAAH1 and MtFAAH2a required glycine residues (Gly^301^ for MtFAAH1 and Gly^308^ for MtFAAH2a) for hydrogen bonding with the head group of *p*-coumaryl-HL (Fig. 3f,g). However, the homoserine lactone (HL) head group of *p*-coumaryl-HL was far more supported in the SBP of MtFAAH2a with additional hydrogens bonds mediated by Glu^213^, Met^345^ and Val^306^.Further, MtFAAH1 positioned Thr^299^ for hydrogen bond interactions with *p*-coumaryl-HL head group whereas MtFAAH2a had the aromatic side-chain of Trp^341^ and the pyrrolidine loop of Pro^338^ for hydrophobic interactions with the HL head (Fig. 3f,g). These data indicate similar organization of the catalytic triad residues to achieve catalysis, and suggest similar and distinct interactions to stabilize the different polar “head regions” of the substrates in the active sites of MtFAAHs.

Analysis of the interactions between the acyl chains of NAEs or *p*-coumaryl-HL and the residues of the SBPs of MtFAAHs revealed certain similarities and differences. The acyl chain of NAE18:2 is supported in MtFAAH1 by hydrophobic interactions with Leu^441^ and an array of three threonine residues (Thr^257^, Thr^258^, Thr^529^). The geometric positioning of these residues in MtFAAH1 appears to provide a flexible environment for accommodation of long acyl chains (Fig. 3b; Supplementary Fig. S9) similar to that reported for AtFAAH1^1^. Binding of NAE18:2 in MtFAAH2a was supported by predicted hydrophobic interactions with Thr^264^, Ile^447^, Ile^540^, and two tyrosine residues, Tyr^533^ and Tyr^444^ (Fig. 3c). Compared to MtFAAH1, the surface of MtFAAH2a binding pocket appeared to be shaped differently to accommodate NAE18:2 (Fig. 3c; Supplementary Fig. S9). It appears that the acyl chain of NAE18:2 may be structurally more compressed in MtFAAH2a, presumably, to adjust to a more restrictive-sized SBP in MtFAAH2a (Fig. 3c; Supplementary Fig. S9). The acyl chain of the shorter NAE (NAE12:0) docked in MtFAAH1 or MtFAAH2a is supported in both cases by van der Waals interactions with the aliphatic amino acid isoleucine (Ile^444^ for MtFAAH1 or Ile^540^ for MtFAAH2a) (Fig. 3d,e). Further, MtFAAH2a has positioned the aromatic residue tyrosine (Tyr^533^) for interactions at positions C11 and C12 of NAE12:0 tail whereas MtFAAH1 displays Leu^441^ for interactions at C10 of the acyl chain of NAE (Fig. 3d,e). Unlike MtFAAH1, MtFAAH2a appears to possess a more aromatic surface to accommodate NAE12:0 into its SBP (Fig. 3d,e; Supplementary Fig. S9). Finally, MtFAAH1 accommodates *p*-coumaryl-HL by predicted van der Waals interactions between Thr^257^ and the aromatic tail of the aryl-HL (Fig. 3f) whereas MtFAAH2a, besides the interactions with similar residues (Ile^447^), also has positioned an aromatic residue, Tyr^477^, for additional hydrogen bonding and π-π interactions with the phenolic acyl moiety of *p*-coumaryl-HL (Fig. 3g; Supplementary Fig. S9). These data suggest that MtFAAH1 or MtFAAH2a can accommodate NAEs or aryl-HLs via residues with distinct physicochemical properties. Indeed, MtFAAH1 appears to generally utilize hydrophobic residues with aliphatic side chains whereas MtFAAH2a consistently relies on neutral residues with aromatic side chains.

### Molecular dynamic simulation experiments of ligand bound MtFAAH1 and MtFAAH2a complexes

**Figure 4:**
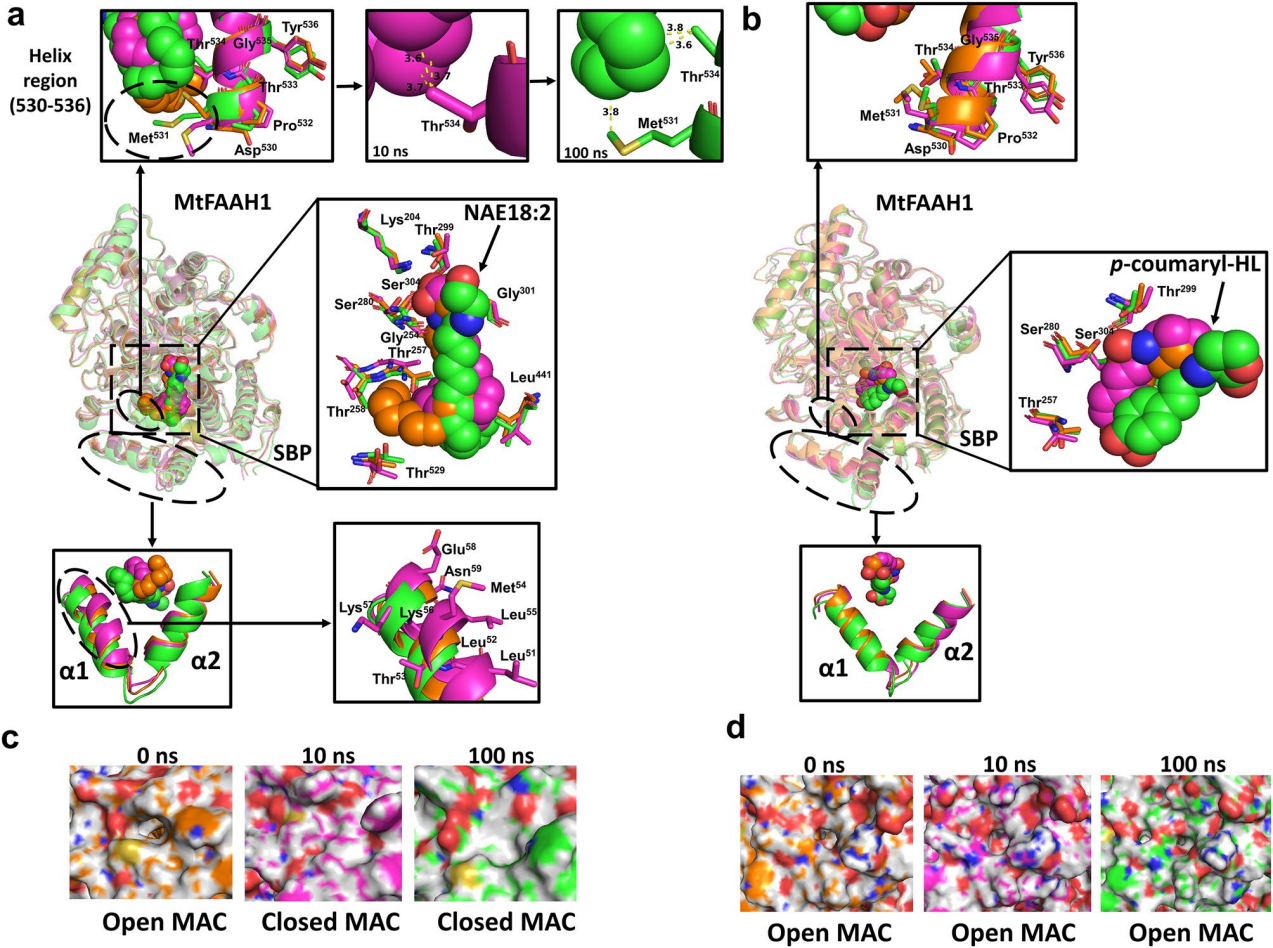
Molecular dynamics simulations (MDS) of subunit A of MtFAAH1 bound to NAE18:2 (**a**, **c**) or *p*-coumaryl-HL (**b**, **d**). “0 ns” corresponds to the equilibrated complexes (orange), “10 ns” and “100 ns” represent MDS processed complexes at 10 (purple) and 100 ns (green). Wider and close-up views of helices regions (residues 530–536) displayed as superimposed cartoons and sticks, substrate binding pocket (SBP) with ligands (spheres) and superimposed residues (sticks) chosen from docking experiments, and predicted α1 and α2 helices are displayed as overlapping cartoons and sticks (**a**, **b**). Numbers displayed in the van der Waals bonds (yellow-dashed lines) represent distances (Å) between the contact points. Close-up surface view of the membrane access channel (MAC) of MtFAAH1 bound to NAE18:2 (**c**) or *p*-coumaryl-HL (**d**).

**Figure 5:**
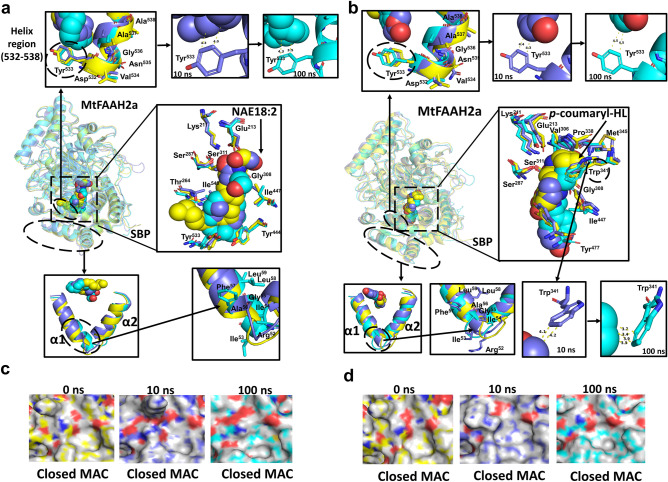
Molecular dynamics simulations (MDS) of subunit A of MtFAAH2a bound to NAE18:2 (**a**, **c**) or *p*-coumaryl-HL (**b**, **d**). “0 ns” corresponds to the equilibrated complexes (yellow), “10 ns” and “100 ns” represent MDS processed complexes at 10 (blue) and 100 ns (cyan). Wider and close-up views of helices regions (residues 530–536) displayed as superimposed cartoons and sticks, substrate binding pocket (SBP) with ligand (spheres) and superimposed residues (sticks) chosen from docking experiments, and predicted α1 and α2 helices are displayed as overlapping cartoons and sticks (**a**, **b**). Numbers displayed in the van der Waals bonds (yellow-dashed lines) represent distances (Å) between the contact points. Close-up surface view of the membrane access channel (MAC) of MtFAAH1 bound to NAE18:2 (**c**) or *p*-coumaryl-HL (**d**).

To further dissect additional residues and mechanisms that could be potentially involved in the accommodation of substrates in MtFAAHs, we carried out molecular dynamic simulation (MDS) on MtFAAH1 or MtFAAH2a docked with NAE18:2 or *p*-coumaryl-HL during 100 ns (Figs. 4, 5; Supplementary Figs. S10, S11; Supplementary Table S6; Supplementary Videos 1–12). The MDS trajectories for MtFAAH1 and MtFAAH2a revealed that NAE18:2 or *p*-coumaryl-HL can be accommodated through distinct predicted interactions.

In the SBP of MtFAAH1, NAE18:2 appears to require accommodation of its acyl chain via Thr^534^ and Met^531^ (Fig. 4a; Supplementary Table S6, Supplementary Video S1, S2). These residues are within a helix region (residues 530 to 536) known to be necessary for the “squeeze” and “lock” mechanism, as reported for AtFAAH elsewhere^1^. At 10 and 100 ns, van der Waals interactions were observed between Thr^534^ and several carbons of the acyl chain of NAE18:2, namely, C14 to C16 and C12–C13, respectively. Additionally, the residue Met^531^ which is located at the end of the helix region, appears to be highly flexible, and could potentially form similar interactions with NAE18:2 at the C10 position, as shown at 100 ns (Fig. 4a). Close inspection at the SBP revealed that the conformation of the acyl tail of NAE18:2 changed over time. Indeed, NAE18:2 tail was highly flexible during the simulation especially from C12 to C18, and oriented itself from C1 to C9 in a straight line at 100 ns (Fig. 4a; Supplementary Video S1, S2). Thus, suggesting a highly flexible SBP suitable for accommodation of acyl amides with long acyl chains. Furthermore, analysis of the MAC revealed an inward movement of the α1 helix (residues 51–58). This motion seems to lead to the closing of the MAC following binding to NAE18:2 (Fig. 4c), which may suggest a “lock” mechanism to confine the substrate in the binding pocket for catalysis (Supplementary Video S3). By contrast, analysis of MtFAAH1 bound to *p*-coumaryl-HL suggested that this aryl-HL is poorly accommodated in the SBP of MtFAAH1 with few potential interactions (Fig. 4b; Supplementary Video S4, S5). Indeed, the helix region (residues 530–536) did not seem to interact at all with the *p*-coumaryl-HL substrate. Further, residues such as Met^531^ and Thr^534^ were far away to interact with the aromatic tail of *p*-coumaryl-HL (Supplementary Video S5), suggesting protein–ligand interaction(s) in this region of the SPB are highly unlikely for *p*-coumaryl-HL. Lastly, no dramatic changes were observed in either the conformation of the *p*-coumaryl-HL structure or in the movement of the α1 helix of MtFAAH1 MAC, resulting in an opened or partially opened MAC during the course of the simulation (Fig. 4b,d; Supplementary Video S6).

Unlike the case with MtFAAH1, the helix region (residues 532–538) of MtFAAH2a showed no dramatic movement toward NAE18:2 substrate upon binding (Fig. 5a; Supplementary Table S6; Supplementary Video S7, S8). Nevertheless, van der Waals interactions were found between Tyr^533^ and the acyl chain of NAE18:2 at C13 and C16 (10 ns), and at C11 and C12 positions (100 ns) (Fig. 5a). No other residue in this region seemed to be involved or in close contact with the acyl chain of NAE18:2. Further, the conformation of the acyl chain of NAE18:2 from C1 to C9 appeared to be highly compressed and with few motions over the course of the simulation. This ligand exhibited multiple contortions at different carbon positions of its tail at the end of the simulation (Fig. 5a; Supplementary Video S7, S8). Different from the more open SBP of MtFAAH1, NAE18:2 fit within the smaller SBP of MtFAAH2a seemed to be much more limited and restrictive. A very slight outward movement of the α1 helix (residues 52–59) was recorded during the course of the simulation; however, this movement did not coincide with the opening or closing of the MAC of MtFAAH2a MAC (Fig. 5c; Supplementary Video S9). Binding of MtFAAH2a to *p*-coumaryl-HL was supported by multiple interactions including the residue Tyr^533^ (Fig. 5b; Supplementary Video S10, S11). Over the simulation time, there were consistent close interactions between this tyrosine residue and the aromatic tail of *p*-coumaryl-HL. These likely π- π interactions could be fixating this ligand in place for catalysis (Fig. 5b; Supplementary Video S11). Furthermore, we also noted consistent hydrophobic interactions between the head HL ring and the residue Trp^341^ located at the cytosolic access channel (CAC) of the SBP of MtFAAH2a (Fig. 5b; Supplementary Video S10). Finally, although some slight outward displacements were detected in the α1 helix (residues 52–59) of the MAC of MtFAAH2a (Fig. 5b), no substantial changes were found and the MAC remained in a closed conformation during the 100 ns simulation (Fig. 5d; Supplementary Video S12).

Altogether these data suggest distinct means for fitting of acylethanolamide substrates into the SBPs of MtFAAH1 or MtFAAH2a enzymes. Our MDS experiments highlight potential residues that could be involved in the preferential accommodation of NAEs or aryl HL substrates by these two enzyme isoforms.

### Recombinant production of MtFAAH1 and MtFAAH2a

**Figure 6:**
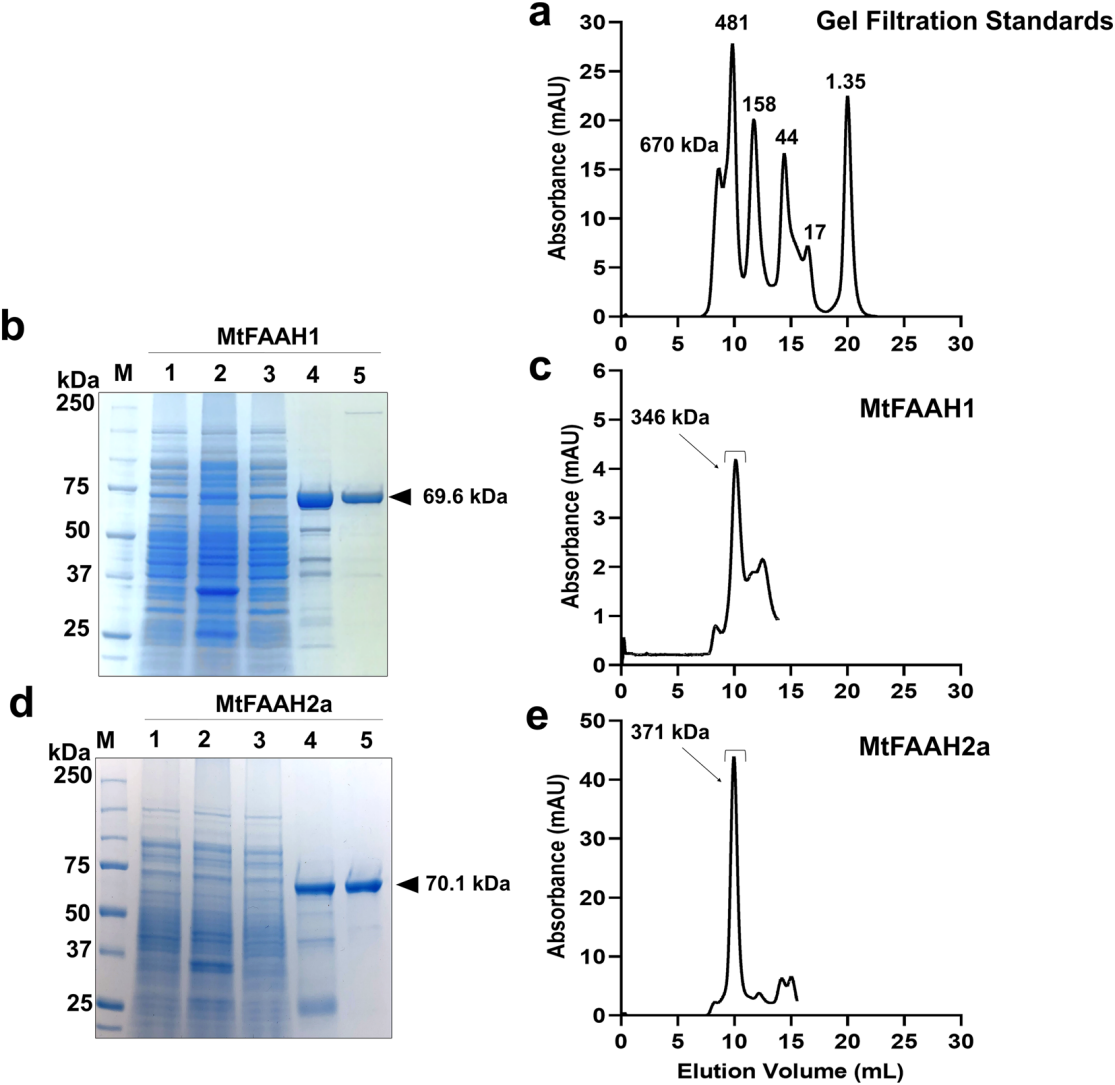
Purification of *Medicago truncatula* FAAH1 and FAAH2a. (**a**) Superdex 200 Increase 10/300 GL chromatogram of gel filtration standards: thyroglobulin, 670 kDa; apoferritin, 481 kDa; γ-globulin, 158 kDa; ovalbumin, 44 kDa; myoglobin, 17 kDa; vitamin B12, 1.35 kDa. (**b**) Coomassie stained SDS-PAGE gel of fractions taken during MtFAAH1 purification. Molecular weight ladder (lane M), cell lysate of BL21 (DE3) cells after sonication (lane 1), pelleted cell debris after sonication (lane 2), flow through of IMAC column (lane 3), IMAC elution (lane 4), size exclusion chromatography purified protein (lane 5). (**c**) Superdex 200 Increase 10/300 GL chromatogram of MtFAAH1 purification. (**d**) Coomassie stained SDS-PAGE gel of fractions taken during MtFAAH2a purification. Molecular weight ladder (lane M), cell lysate of BL21 (DE3) cells after sonication (lane 1), pelleted cell debris after sonication (lane 2), flow through of nickel affinity column (lane 3), nickel affinity purified protein (lane 4), size exclusion chromatography purified protein (lane 5). (**e**) Superdex 200 Increase 10/300 GL chromatogram of MtFAAH2a purification. Arrows and brackets point at the SEC-purified fractions that were analyzed by SDS-PAGE and used for enzyme activity assays. Uncropped/unedited SDS-PAGE gels displayed in (**b**) and (**d**) can be found in Supplementary Figs. S19, and S20, respectively.

To test the functional differences between MtFAAH1 and MtFAAH2, we cloned MtFAAHs (Supplementary Fig. S12; Supplementary Table S8) into expression vectors as His-tagged fusions for production of recombinant proteins in *E. coli*. To purify the recombinant proteins, we utilized immobilized metal (nickel) affinity chromatography (IMAC) and size exclusion chromatography (SEC) techniques (Fig. 6). We observed prominent bands at ≈ 70 kDa representing MtFAAH1 and MtFAAH2a from the IMAC-purified and SEC-purified samples (Fig. 6b,d). These bands were equivalent to the calculated molecular weights (MW) of MtFAAHs proteins. To assess whether the oligomerization states of each purified MtFAAH differ from that of AtFAAH^1^, the elution volumes from the chromatograms of gel filtration standards and AtFAAH were used as reference for MtFAAH calculations (Fig. 6a; Supplementary Fig. S13). We observed an average elution volume of 10.08 mL (at pH 9.0) and 10.14 mL (at pH 7.5) for MtFAAH1 and MtFAAH2a, respectively (Supplementary Fig. S13). In solution, the MW of MtFAAH1 was estimated as ≈ 346 kDa and MtFAAH2a as ≈ 371 kDa (Fig. 6c,e; Supplementary Fig. S13). Considering the molecular weights of individual subunits of MtFAAH1 and MtFAAH2 as 69.6 or 70.1 kDa, respectively (Fig. 6; Supplementary Fig. S13), and the reported MW of DDM micelles as ≈ 70 kDa^35^, we estimated that MtFAAH1 and MtFAAH2a were each purified as tetramers (Supplementary Fig. S13). The uncropped/unedited SDS-PAGE gels used for Fig. 6b,d can be found in Supplementary Figs. S19, and S20, respectively whereas the original gels used for Supplementary Fig. S13 can be found in Supplementary Figs. S21–S23.

Additionally, we utilized liquid chromatography with tandem mass spectrometry (LC/MS/MS) to confirm the identity of purified MtFAAH and AtFAAH recombinant proteins (Supplementary Figs. S14, S15, S16). MtFAAH1, MtFAAH2a, and AtFAAH had coverage values of 77% (43 unique peptides), 37% (17 unique peptides), and 100% (243 unique peptides), respectively. Taken together, these results demonstrate the production and purification of MtFAAH1 and MtFAAH2a proteins, and suggest that both purified MtFAAH protein complexes are larger than the AtFAAH dimers^1^, likely organized as homotetramers. The uncropped/unedited gels used for Supplementary Figs. S14–S16 can be found in Supplementary Figs. S24–S26.

### Assessment of MtFAAH1 and MtFAAH2 enzyme activities

As an initial characterization step, the enzymatic activities of MtFAAH1 or MtFAAH2a towards NAE12:0 were measured at different pH or temperature conditions (Supplementary Fig. S17). SEC-purified proteins were incubated with 100 µM NAE12:0 in reaction buffers with different pH (6.0, 7.0, 8.0, and 9.0) or temperature (20, 30, 40, and 50 °C) conditions. A flexible, fluorescamine-based assay was utilized for detection of reaction products, as reported elsewhere^22,36^. Enzyme activity was calculated as μmol of ethanolamine produced per minute per milligram of protein based on a standard curve for ethanolamine. MtFAAH1 activity was the highest at pH 8.0 or 9.0 (Supplementary Fig. S17), whereas MtFAAH2a operated maximally at pH 7.0 or 8.0 (Supplementary Fig. S17). Notably, the activity of MtFAAH2a dramatically decreased at pH 9.0. Temperate dependencies were similar for both MtFAAH1 and MtFAAH2a, with optimal enzyme activities at temperatures between 30 and 40 °C (Supplementary Fig. S17). GC–MS further confirmed conversion of NAE12:0 into its corresponding free fatty acid (FFA) (Supplementary Fig. S18) product for both MtFAAH enzymes and the positive control, AtFAAH; boiled (“denatured”) enzymes were used as negative controls and showed no conversion of substrate to product. The identity of NAE12:0 peak was confirmed by retention time and diagnostic ions (Supplementary Fig. S18) as reported elsewhere^23^. The identity of FFA 12:0 (dodecanoic acid) was confirmed by diagnostic ions (Supplementary Fig. S18) and via NIST library comparison (≈ 98% match). In the reactions with boiled MtFAAH1, MtFAAH2a, or AtFAAH, only the peaks corresponding to NAE12:0 substrate appeared in the chromatograms (Supplementary Fig. S18).

To evaluate the progress of MtFAAH1 or MtFAAH2a purification, enzyme activities were measured in cell lysates, IMAC-, and SEC-purified fractions towards NAE12:0 (100 µM) (Supplementary Table S7). Purification levels and yields were calculated relative to the crude lysates. MtFAAH1 and MtFAAH2a were estimated to be enriched over the cell extracts by 1162-fold and 3263-fold, respectively. SDS-gel electrophoresis confirmed the purity of each protein in the SEC-fractions (Fig. 6; Supplementary Fig. S13).

**Figure 7:**
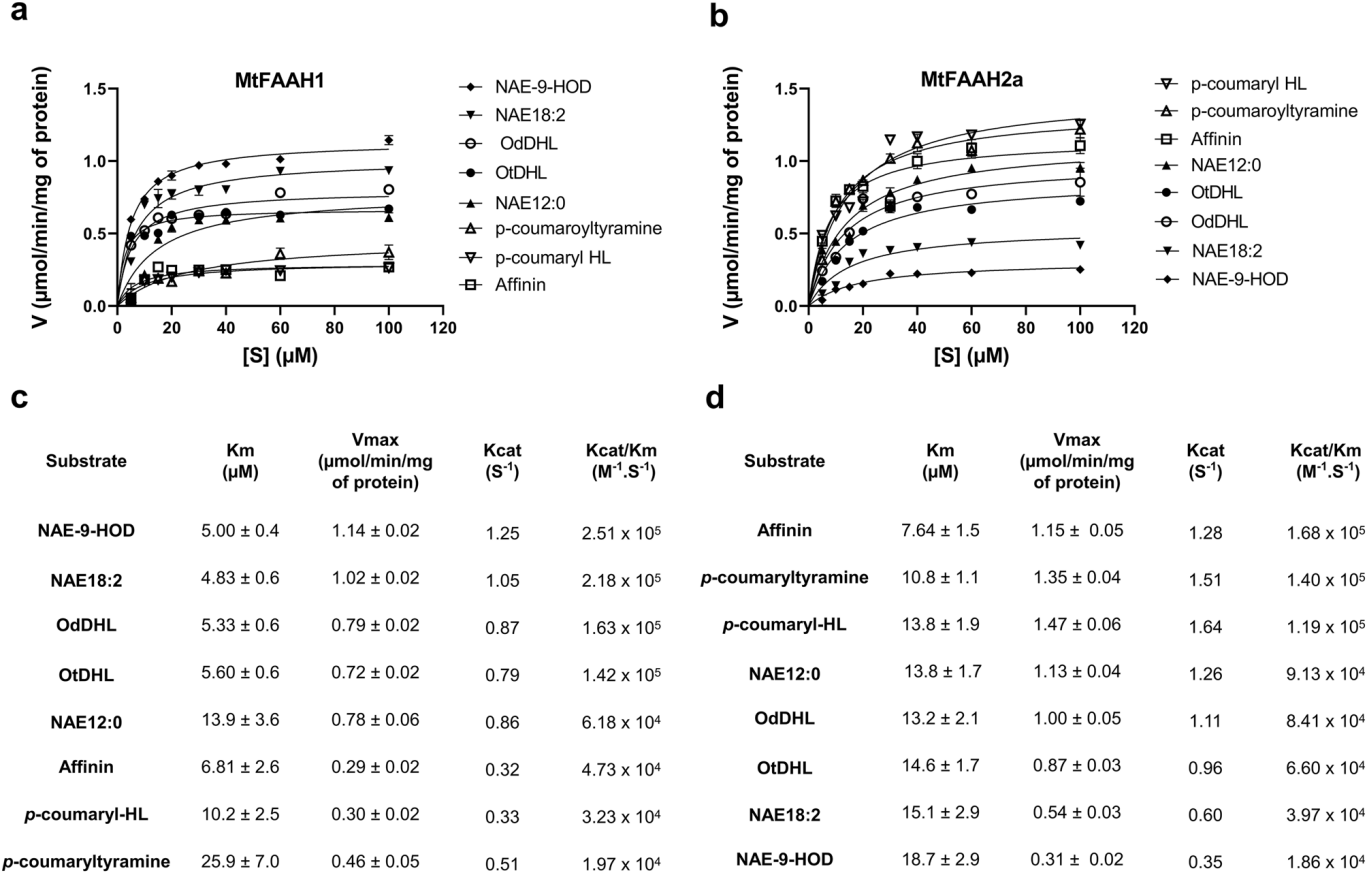
Enzyme kinetic curves for (**a**) MtFAAH1 and (**b**) MtFAAH2a. MtFAAHs were incubated with increasing concentrations of NAE12:0, NAE18:2, NAE-9-HOD, OtDHL, OdDHL, *p*-coumaryl HL, *p*-coumaryltyramine, or affinin. Reactions were conducted in reaction buffer (25 mM HEPES, 100 mM NaCl) at pH = 9 and 0.03% DDM for MtFAAH1 and at pH = 7.5 and 0.06% DDM for MtFAAH2a. In the X-axis different substrate (S) concentrations (5–100 µM) are displayed. In the Y-axis the velocity of reaction (V) is reported as μmol of amide produced per unit of time (min) per amount of protein used (mg). Km (Michaelis and Menten constant) and V were calculated in GraphPad Prism 8.0. Kcat (turnover number)/Km ratio denote the apparent amidohydrolase efficiency of (**c**) MtFAAH1 or (**d**) MtFAAH2a towards a given substrate. Data represent means ± S.D. of triplicate assays.

**Figure 8:**
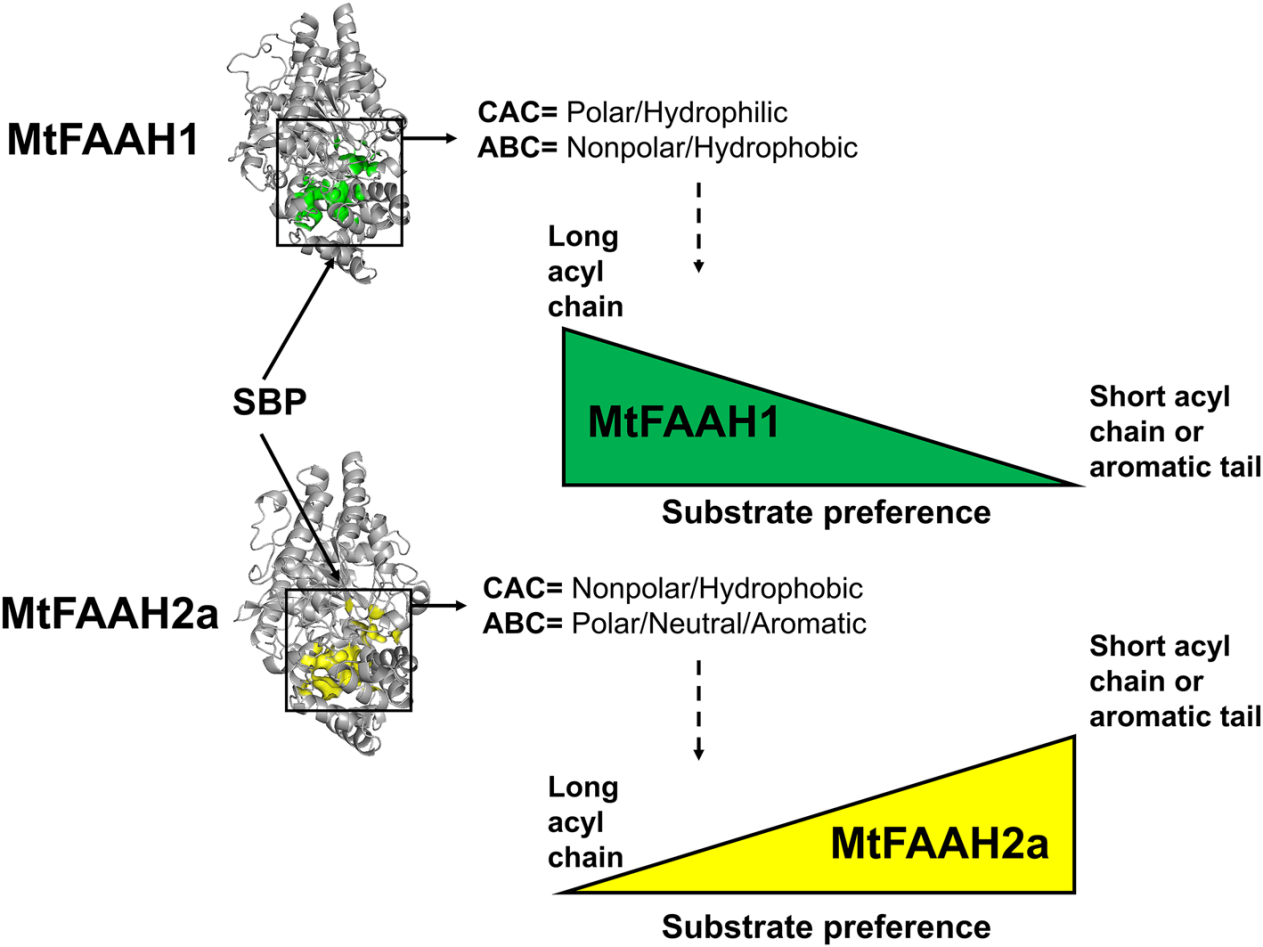
Simplified model showing the apparent amidohydrolase efficiencies of MtFAAH1 or MtFAAH2a towards selected *N*-acyl amides. The model proposes that structural and physicochemical differences between the substrate binding pockets (SBPs) of these two MtFAAHs, specifically at the cytosolic access channel (CAC) and acyl binding channel (ABC), are associated with FAAH selectivity to a given substrate. Indeed, computational and biochemistry data suggest a scenario in which MtFAAH1 prefers to hydrolyze long *N*-acyl amides whereas MtFAAH2a is better at hydrolyzing substrates with either a short acyl chain or an aromatic tail.

Given the key residue differences between the SBPs of MtFAAH1 and MtFAAH2a, and the fact that docking experiments suggest differential accommodation of *N*-acyl substrates, we hypothesized that such differences could result in distinct substrate preferences for MtFAAH1 and MtFAAH2a. Increasing concentrations of NAE12:0, NAE18:2, NAE-9-HOD, OdDHL, OtDHL, *p*-coumaryl-HL, *p*-coumaryltyramine, and affinin were incubated with purified MtFAAH1 or MtFAAH2a at 30 °C for 30 min (Fig. 7). Fluorescamine-based assays were used for detection of reaction products. Initial velocity (V) of the reactions are presented as μmol of amine produced per minute per milligram of protein. The Kcat/Km ratios derived from Michaelis and Menten plots were used to compare the catalytic efficiencies of the enzymes for their substrates (Fig. 7). MtFAAH1 had the highest Kcat/Km ratios for long chain NAEs (NAE-9-HOD or NAE18:2), or AHLs (OdDHL or OtDHL). Then, compared to OtDHL, Kcat/Km ratios decreased by ≈ 2- to threefold for NAE12:0 or affinin. The Kcat/Km values for *p*-coumaryl-HL or *p*-coumaryltyramine were the lowest among all substrates tested for MtFAAH1 (≈ 7- to 12-fold lower compared to NAE-9-HOD) (Fig. 7a,c). By contrast, MtFAAH2a had the highest Kcat/Km values for affinin, *p*-coumaryltyramine, or *p*-coumaryl-HL. Compared to *p*-coumaryl-HL, the Kcat/Km of MtFAAH2a decreased by ≈ twofold for NAE12:0, OdDHL or OtDHL. Notably, MtFAAH2a had the lowest Kcat/Km ratios for NAE18:2 or NAE-9-HOD (≈ 4- to ninefold lower than that of affinin) (Fig. 7b,d). These data indicate that under these in vitro conditions, MtFAAH1 prefers lipophilic substrates with longer acyl moieties whereas MtFAAH2a performs best with substrates with short or aromatic acyl moieties (summarized in Fig. 8).

## Discussion

FAAH hydrolyzes NAE or NAE-like structures into their corresponding amine (head group) and free fatty acid (tail group) products^1,30,37^. Recently, structural and phylogenetic comparisons of FAAHs from multiple angiosperms led to the discovery of two groups of FAAHs, FAAH1 and FAAH2, suggesting a more complex FAAH-acylamide substrate profile than what was previously thought^32^. Here, two FAAH isoforms from the legume *Medicago truncatula* were compared at the structural (predicted) and functional levels for the first time, revealing surprising findings. Although both isoforms hydrolyze a range of acylamide substrates, they do so with opposite efficiencies suggesting that one evolutionary consequence of elaboration of FAAHs in angiosperms is to expand the range of acylamide substrates that can be utilized by these enzymes. These differences are only now being revealed by looking at FAAH isoforms distributed outside the Brassicaceae^32^, where only one FAAH (FAAH1) occurs, and where most molecular and biochemical studies have been conducted.

In kinetic comparisons of these FAAHs in vitro, MtFAAH1 appeared to utilize long chain NAEs (e.g. NAE18:2) better than shorter chain or aromatic substrates. By contrast, MtFAAH2a was far less efficient at hydrolysis of long chain NAEs, and instead hydrolyzed short chain or aromatic substrates (e.g. *p*-coumaryl-HL) with ~ 10 times greater efficiency. Docking and molecular dynamic simulation (MDS) experiments with substrates in the SBPs of these two FAAHs supported these differences in enzyme behavior. MtFAAH1 was predicted to have a more open and less restrictive SBP and a more flexible membrane access channel (MAC) that would better accommodate long-chain NAEs (e.g. NAE18:2). By contrast, the SBP of MtFAAH2a with bound NAE18:2 was shaped differently and led to a more compressed orientation of the longer acyl chain and an unaltered closed MAC. Similarly, recombinant AtFAAH (a FAAH1) hydrolyzed a range of NAEs and AHLs in a manner that was dependent on the length the acyl chain rather than the polar head group^22^. Indeed, it has been shown elsewhere that AtFAAH activity was the highest for long-chain AHLs (e.g. OdDHL or OtDHL), and lowest for shorter AHLs (e.g. OHHL or OOHL)^22^. Similarly, we observed that MtFAAH1 was significantly better at hydrolyzing OdDHL or OtDHL than short-chain or aromatic acyl substrates (e.g. *p*-coumaryl-HL). On the other hand, the short chain or aromatic acylamides were accommodated well in the SBP of MtFAAH2a; in the docking and MDS experiments with an aryl-HL, both the lactone ring head and aromatic tail of *p*-coumaryl-HL appeared to be better accommodated in the SBP of MtFAAH2a than in MtFAAH1, perhaps attributed in part to the presence of large aromatic residues in MtFAAH2a (e.g. Tyr^533^, Tyr^444^, Trp^341^). Alkamides (e.g. affinin) are poor substrates for AtFAAH^20^. Here, kinetic data for MtFAAH1 support a similar conclusion where MtFAAH1 more efficiently hydrolyzed long-chain NAEs than affinin. By contrast, MtFAAH2a exhibited the highest catalytic efficiencies toward affinin when compared to NAEs, and it is likely the more non-polar residues in the cyctosolic access channel of MtFAAH2a helped to support the interaction with alkamides. Altogether, it appears that the structural differences between the SBPs of MtFAAH1 and MtFAAH2a and their predicted interactions with substrates were consistent overall with observed differences in hydrolytic efficiencies (Fig. 6). Notably, the quorum sensing molecule *p*-coumaryl-HL^38^ was an efficient substrate for MtFAAH2a. It is tempting to speculate that *M. truncatula* has evolved a FAAH2 machinery that expands the plants’ chemical “communication” range for microbial interactions. Although the biological implications *p*-coumaryl-HL hydrolysis by MtFAAH2a are beyond the scope of this study, our results provide interesting ideas for testing the potential function(s) of MtFAAH2a *in planta*.

The observed divergence in substrate preferences between the two Medicago FAAH isoforms is somewhat reminiscent of the concept in placental mammals where two diverged FAAH isoforms are present. In humans, the hydrolytic rates of two different FAAHs (≈ 20% identity) showed some distinct and overlapping preferences towards different substrates^39^. In that study, a FAAH1 was far more efficient at hydrolyzing polyunsaturated substrates (e.g. NAE20:4) whereas FAAH2 preferred monounsaturated acyl amides (e.g. oleamide). In the same report; FAAH1 was capable of processing the amino acid conjugated acyl amide, *N*-oleoyl-taurine (NAT), whereas FAAH2 was incapable of utilizing this substrate. Interestingly, both FAAHs similarly hydrolyzed NAE18:1^39^. At least for mammalian FAAH1, it is reported that substrate binding in the active site is influenced by two aromatic amino acid residues that form a “dynamic paddle” and this may contribute to substrate selectivity^1,6^. A third type of amide hydrolase in mammals has a very strict substrate preference. In rabbit (*Oryctolagus cuniculus*), a cysteine hydrolase, designated *N*-acylethanolamine acid amidase (NAAA) was shown to have a marked preference towards NAE16:0, and this seems to be dependent on the nature of its SBP. Indeed, it appears that through evolution, NAAA acquired a restrictively shaped SBP that is optimal for NAE16:0, and less efficient towards other acyl amides of longer or shorter acyl chain^40^. Thus, similar to mammals, the legume *M. truncatula* may have elaborated FAAH isoforms that selectively process a range of acylamide substrates.

Biochemical properties of what are now considered to be group 1 FAAHs have been characterized previously from Arabidopsis^1,41^, rice^42^, *Medicago truncatula*^42^ and a moss (*P. patens*)^9^, although not all studies were carried out with purified enzymes, and not all FAAH1 homologs were tested with a broad range of acylamide substrates. Here, the biochemical properties of a group 2 FAAH enzyme are described for the first time, and some similarities and differences with group 1 FAAH isoforms were noted in the progress of purification and in the assays of enzymatic activity. In addition to differences in apparent substrate preferences as described above, other features of the enzymes were different. The isoelectric point of MtFAAH2a is significantly higher than that of MtFAAH1 (or other FAAH1 proteins) which required altered conditions for solubility during the course of purification (and also likely influenced the pH optimum for assays in vitro). The consideration of buffer pH in relation to the protein’s isoelectric point is important for recombinant protein purification, as reported elsewhere^43^. In addition, MtFAAH2 required twice the concentration of DDM compared with MtFAAH1 to maintain solubilization during the progress of purification. Despite these differences, both MtFAAH1 and MtFAAH2a were purified to near homogeneity, and based on their migration relative to known standards, appeared to both resolve as homomeric tetramers in SEC. Overall, the factors identified here will facilitate future studies of FAAH enzymes in other plant species.

Our data consistently supported the notion that both MtFAAH1 and MtFAAH2a can be found as tetrameric oligomers when purified in solution with detergent, whereas AtFAAH was solubilized as a dimer. Previous reports support oligomeric states for FAAHs of multiple organisms. For example, the crystal structure of FAAH from Arabidopsis or the fungus *Candida albicans* indicated dimers^1,44^. Notably, Rat FAAH is also reported as a dimer when purified in solution but can form three octamers of dimers in the absence of detergent^45^. The evident size differences of the apoenzymes purified here from *Medicago truncatula* (different from the only other plant FAAH, AtFAAH, for which there is a structure) is intriguing and may have important functional implications, but the quaternary structure and its functional relevance must await future studies. Further, it remains to be seen if this predicted tetrameric organization is a feature of FAAH isoforms from other plant species outside the Brassicaceae.

In conclusion, MtFAAHs differentially hydrolyze a wide range of NAEs, acyl-HLs, aryl-HLs, alkamides, or phenolic amides. Such outcomes coincide with differences between the predicted structural organization of the SBPs of MtFAAH1 and MtFAAH2a. Although in our investigations we screened the in vitro capabilities of MtFAAHs against substrates reported to be either endogenously accumulated in plant tissues (e.g. NAEs) or in bacteria (e.g. AHLs), it is possible that additional lipophilic substrates with physiological relevance are yet to be discovered. Further studies at the genetic and physiological levels will help to unravel the function of additional FAAH isoforms in *Medicago truncatula*. Moreover, additional examination of FAAH isoforms in other plant species will be required to determine the broader significance of FAAH-mediated acyl amide hydrolysis in plants. Nevertheless, this study provides a foundation for future work to uncover potential physiological significance and possible biotechnological applications.

## Materials and methods

### Phylogenetic analysis and homology modeling

The legume sequences used for Fig. 1a were retrieved from NCBI database (Supplementary Table S1). Phylogenetic analysis was carried out with “Phylogeny.fr” online tool with default settings^46^. Sequence alignment was conducted in Clustal Omega^47^ (Supplementary Figs. S1, S7). MtFAAH1 and MtFAAH2a homology models were built with Arabidopsis FAAH as template (AtFAAH; 6DHV) in SWISS-MODEL^48^. 3D models were visualized in Pymol^49^ and BIOVIA Discovery Studio Visualizer^50^. Residues that comprise membrane binding cap (MBC), membrane access channel (MAC), helix region (predicted to be involved in acyl amide tail accommodation), and substrate binding pocket (SBP) structures for MtFAAHs were predicted from details in the AtFAAH structure^1^ (Figs. 4, 5; Supplementary Table S5, S6). Antheprot3D was used for prediction of secondary structure content^51^ (Supplementary Fig. S5). The quality parameters for the MtFAAH models generated in SWISS-MODEL include; GMQE (Global Model Quality Estimate), QMEANDisCo (Qmean consensus-based distance constraint) scores, and Ramachandran plots and scores (Supplementary Figs. S3, S4; Supplementary Table S3, S4).

### Transcript expression profiles

The expression profiles of *MtFAAH1*, *MtFAAH2a*, and *MtFAAH2b* were retrieved from the *Medicago truncatula* Gene Expression Atlas “MtExpress”^34^. The corresponding MtFAAH sequences (see Supplementary Table S1 for accession numbers) were used as input in the BLAST tool built within the Atlas.

### Molecular dynamic simulation experiments of apo MtFAAHs

The topologies of the apo forms of MtFAAH1 and MtFAAH2a were carried out with Amber force field (ff19SB)^52^. A cubic box of 12 Å was constructed around the apo MtFAAH isoforms, and the systems were solvated in TIP3P water^53^. Neutralization of the charges was conducted by Na^+^ and Cl^-^ ions at a concentration of 0.15 M. The system was subjected to energy minimization with a temperature of 298 K, and pressure of one bar. Then, the systems were equilibrated with a force constant of 500 kJ/mol during 5 ns. Molecular dynamic simulations (MDS) were carried out in the cloud-based molecular simulations environment developed elsewhere^54^. This pipeline utilizes the Open MM toolkit^55^ to generate constant temperature, constant pressure (NPT) ensembles. The MD environment had a temperature of 298 K and pressure of one bar. The GPU and computer units in Google Collab were used to run 100 ns simulations for both MtFAAHs. Each simulation took 12 to 13 h to reach completion. RMSD and RMSF trajectories were generated with the MDAnalysis^56^ or PyTraj^57^ toolkits, and then further processed in Excel 2021 (Supplementary Fig. S8).

### Molecular docking experiments

All 2D structures were drawn using ChemDraw (Molecular Editor) software. Substrates and enzymes were prepared as follows; the SDF files of NAE18:2 (CID: 5,283,446), NAE12:0 (CID: 8899) or *p*-coumaryl-HL (CID: 71,311,837) were retrieved from PubChem. Then, Babel 3.0.1^58^ was used to convert the SDF files into PDB format. The PDB files of the apo MtFAAH structures generated after MDS of 100 ns (see above) were used for docking experiments prior removal of water, Na^+^ and Cl^-^ ions. Hydrogens were added to substrates and MtFAAH1 or MtFAAH2a protein models in Pymol^49^. Energy minimization was conducted in PyRx^59^ with default settings.

Docking in GOLD 3.0.1 software^60^ was centered at the covalent bond between the side-chain oxygens (Oγ) of the catalytic residues of MtFAAH1 (Ser^304^; atom number 4628) or MtFAAH2a (Ser^311^; atom number 4678) and the carbon of the carbonyl of NAE18:2, NAE12:0 or *p*-coumaryl-HL. The cavity detection was set with a radius of 20 Å around the active site. Spring constants between 50 and 500, and minimum (1.30 Å) and maximum (1.60 Å) separations were used in the constraint settings. Annealing parameters for van der Waals and hydrogen bonding were set at 6 and 3 Å, respectively. The algorithm was set with external and internal energy values of 1.4 and 1.0, respectively. Predicted poses were ranked on the basis of the fitness GOLD score. The pose with the highest score was chosen for further analysis. All poses chosen in this study had GOLD scores above 10. Docking predictions were visualized in Pymol^49^ and BIOVIA Discovery Studio Visualizer^50^. Putative interactions such as hydrogen bonds, and hydrophobic interactions were predicted in Protein–Ligand Interaction profiler (PLIP) server^61^.

### Molecular dynamic simulations of MtFAAH bound ligand complexes

The topologies of proteins (MtFAAH1 or MtFAAH2a) and ligands (NAE18:2 or *p*-coumaryl-HL) were generated with the Amber force fields ff19SB^55^ and GAFF2^62^, respectively. A cubic box of 15 Å was constructed around the protein–ligand complexes, then systems were solvated in TIP3P water^53^. Neutralization of the charges was conducted by Na^+^ and Cl^-^ ions at a concentration of 0.15 M. The system was subjected to energy minimization with a temperature of 298 K, and pressure of one bar. The systems were equilibrated with a force constant of 1600 kJ/mol during 5 ns. MDS experiments were carried out in the cloud-based molecular simulations pipeline developed elsewhere^54^. The same temperature and pressure used for equilibration were used for MD production. The GPU and computer units in Google Collab were used to run 100 ns simulations for all MtFAAH-ligand complexes. Each simulation took 12–13 h to reach completion. The trajectories and log files were recorded every 100 ps. Further, to mimic the covalent bond interaction between the side-chain oxygens (Oγ) of the catalytic residues of MtFAAHs and the carbon of the carbonyl group of the ligands, we added a harmonic constraint between those atoms under the MonteCarloBarostat tool in the pipeline “Make it rain”^54^. RMSD and RMSF trajectories were generated with the MDAnalysis^56^ or PyTraj^57^ toolkits, and then processed in Excel 2021 (Supplementary Fig. S10). Pearson’s Cross Correlation analysis was generated within the same pipeline^54^ (Supplementary Fig. S11). The equilibrated complexes (time = 0 ns), and the MD processed complexes at 10 ns and 100 ns were processed in Pymol^49^ by removing water and Na^+^ and Cl^-^ ions from their structures (Figs. 4 and 5). The PDB files and dcd trajectories of MtFAAH-ligand complexes were loaded in VMD software^63^ for analysis of the MD simulation. VideoMach^64^ was coupled with VMD to generate the videos corresponding to MDS experiments (Supplementary Videos S1–S12).

### MtFAAH constructs

Qiagen RNeasy Plant Mini Kit was used for RNA extraction from stems of 21-day-old *Medicago truncatula* Gaertner (wild-type genotype Jemalong A17) seedlings. *M. truncatula* (A17) seeds were a gift from Dr. Rebecca Dickstein at the University of North Texas (UNT), as reported and utilized elsewhere^65^. Seedlings used for RNA extraction were grown in lighted chambers, and their use complied with international, national and/or institutional guidelines. cDNA synthesis was carried out with Applied Biosystems High Capacity cDNA Reverse Transcription Thermo Fisher kit, following the instructions of the manufacturer. Two rounds of PCR (nested PCR) were needed to yield the full-length coding sequence (CDS) of MtFAAH1 (Supplementary Fig. S12). The stop codon of MtFAAH1 was removed from its CDS with specific primers (Supplementary Table S8). Phusion High-Fidelity DNA Polymerase (New England BioLabs) was used for high fidelity PCR. The 1 Kb MassRuler DNA ladder (Thermo Fisher) was used to estimate PCR product size. Insertion of MtFAAH1 CDS into pTrcHIS2 plasmid (Invitrogen) was achieved by TA cloning, following the instructions of the manufacturer.

The CDS of MtFAAH2a was synthesized by GENEWIZ, and inserted into pUC57-Amp vector. Then, specific primers (Supplementary Table S8) were used for removal of the stop codon and sub cloning into pTrcHIS2 plasmid (Invitrogen) (Supplementary Fig. S12), as described above. All vectors were confirmed as correct by DNA sequencing prior to heterologous expression.

### MtFAAH recombinant protein production and purification

Recombinant MtFAAH1 was produced in *E. coli* BL21 (DE3) cells from the pTrcHIS2 plasmid (Invitrogen) with C-terminal c-myc and histidine (6X) tags. Overnight cultures were grown with 100 µg/mL ampicillin (GoldBio) at 37 °C and were inoculated into fresh LB containing 100 µg/mL ampicillin and grown at 37 °C until OD_600_ reached 0.5–1.0. Protein production was induced with 1 mM isopropyl β-D-1-thiogalactopyranoside (GoldBio) for 18 h at 16 °C. The cells were harvested by centrifugation at 4000 X*g* for 30 min at 4 °C then frozen at − 80 °C overnight. After thawing in lysis buffer (50 mM Tris pH 8.0, 100 mM NaCl, 1% (v/v) Triton X-100), 1 mM EDTA, 1 µM E-64, 1 µM pepstatin, 1 mg/mL lysozyme (Sigma) and 25 units/mL of Benzonase nuclease (Sigma) were all added. The cell pellet was suspended by rotation at 30 RPM for 30 min at 4 °C then sonicated (Ultrasonic processor GEX 130) for 8 min at 20% intensity with 30 s on pulses and 30 s off pulses. Cell debris was pelleted at 14,000 X*g* for 30 min at 4 °C and the lysate was incubated with Ni–NTA agarose resin (Qiagen) and suspended by rotation at 30 RPM for 1 h at 4 °C. After the cell lysate was completely separated from the resin using a gravity flow column, the resin was washed with two buffers: (1) 50 mM Tris–HCl pH 8.0, 500 mM NaCl, 1% Triton X-100, 10 mM Imidazole; (2) 50 mM Tris–HCl pH 8.0, 500 mM NaCl, 0.03% (w/v) dodecyl beta-D-maltoside (DDM), 25 mM Imidazole. The His-tagged, recombinant protein was then eluted from the resin with 50 mM Tris–HCl pH 8.0, 500 mM NaCl, 0.03% DDM, 250 mM imidazole, 1 mM EDTA, 1 µM E-64, 1 µM pepstatin. DTT (1 mM) was added fresh to the eluted fraction and stored at 4 °C overnight. The next day the eluted fraction was concentrated and exchanged to 50 mM Bis–Tris-Propane pH 9.0, 100 mM NaCl, 0.03% DDM using a 30,000 MWCO centrifugal filter device (Sartorius). Recombinant protein was further purified and fractionated using a Superdex 200 increase 10/300 GL column (Cytiva) on an AKTA Pure system (Cytiva). SEC-eluted fractions were observed at UV absorbance of 280 nm, fractionated and collected for SDS-PAGE analysis, LC/MS/MS, or enzyme activity assays.

Recombinant MtFAAH2a was purified with the same methods as MtFAAH1 (see above) except for buffer pH and detergent concentration. For MtFAAH2a, all buffers were at pH 7.5 and 0.06% DDM during the purification process. SEC fractions were collected for SDS-PAGE analysis, LC/MS/MS, and enzyme activity assays.

### LC/MS/MS analysis of MtFAAHs

Gel bands were dehydrated using 100% acetonitrile and incubated with 10 mM dithiothreitol in 100 mM ammonium bicarbonate, pH ≈ 8, at 56 °C for 45 min, dehydrated again and incubated in the dark with 50 mM iodoacetamide in 100 mM ammonium bicarbonate for 20 min. Gel bands were then washed with 100 mM ammonium bicarbonate and dehydrated again. Sequencing grade modified trypsin, chymotrypsin, and Asp-N was prepared to 0.01 µg/µL in 50 mM ammonium bicarbonate and ≈ 50 µL of this was added to each gel band so that the gel was completely submerged. Bands were then incubated at 37 °C overnight. Peptides were extracted from the gel by water bath sonication in a solution of 60% Acetonitrile/1% TCA and vacuum dried to ≈ 2 µL.

Peptides were then re-suspended in 2% acetonitrile/0.1% TFA to 20 µL. From this, 5 µL were automatically injected by EASYnLC 1000 onto a Thermo Acclaim PepMap 0.1 mm × 20 mm C18 peptide trap and washed for ≈ 5 min. Bound peptides were then eluted onto a Thermo Acclaim PepMap RSLC 0.075 mm × 250 mm C18 column over 35 min with a gradient of 5% B to 38% B in 24 min, ramping to 90% B at 25 min and held at 90% B for the duration of the run at a constant flow rate of 0.3 µL/min (Buffer A = 99.9% Water/0.1% Formic Acid, Buffer B = 99.9% Acetonitrile/0.1% Formic Acid). The column was maintained at 50 °C using an integrated column heater (PRSO-V1, Sonation GmbH, Biberach, Germany).

Eluted peptides were sprayed into a ThermoFisher Q-Exactive mass spectrometer using a Flex Spray spray ion source. Survey scans were taken in the Orbitrap (70,000 resolution, determined at m/z 200) and the top 15 ions in each survey scan were then subjected to automatic higher energy collision-induced-dissociation (HCD) with fragment spectra acquired at 17,500 resolution. The resulting MS/MS spectra were converted to peak lists using Mascot Distiller, v2.8.0.1 (www.matrixscience.com) and searched against a database that included FAAH protein sequences and, *E.coli* protein sequences available from Uniprot (www.uniprot.org) appended with common laboratory contaminants (downloaded from www.thegpm.org, cRAP project) using the Mascot searching algorithm, v 2.7.1. The Mascot output was then analyzed using Scaffold, v5.0.1 (www.proteomesoftware.com) to probabilistically validate protein identifications. Assignments validated using the Scaffold 1% FDR confidence filter are considered true. The mass spectrometry proteomics data have been deposited to the ProteomeXchange Consortium^66^ via the PRIDE^67,68^ partner repository with the dataset identifier PXD038494 and https://doi.org/10.6019/PXD038494.

### Molecular weight and oligomerization estimation

Standard curves were composed by plotting the distribution coefficient (K_av_) versus the logarithm of molecular weights of known standards (Thyroglobulin, γ-globulin, ovalbumin, myoglobin, and vitamin B12, Bio-Rad; apoferritin, Sigma) according to the Cytiva SEC Handbook, Appendix 6. Two separate standards and standard curves were prepared in relation to the different conditions used to purify the MtFAAHs Then, the K_av_ of each FAAH was calculated using the average elution volume of the peak from six purifications performed on different days. After interpolating this value onto the respective curve, we calculated the molecular weight and estimated the oligomerization states after taking the detergent aggregation number into account^35^. Calculations were made in GraphPad Prism 8.0.

### SDS-PAGE gel electrophoresis and Coomassie staining

Samples taken during the progress of the purification were quantified by BCA Rapid Gold assay (Thermo) and separated in a prepared 10% resolving gel by SDS-PAGE (Bio-Rad). Ten micrograms of the cell lysate, pelleted cell debris, and flow through along with 2 µg of the nickel-affinity purified protein and 2 ug of the SEC purified protein were subjected to SDS-PAGE. Samples were denatured in 4 × Laemmili buffer (Bio-Rad) with 10% (v/v) β-mercaptoethanol and electrophoresis was conducted at 175 constant volts for 50 min. Gels were then stained with QC Colloidal Coomassie blue stain (Bio-Rad) according to the manufacturer’s protocol (Fig. 6b,d; Supplementary Figs. S14–S16, S19, S20, S24–26). Alternatively, BIO-RAD stain-free gels were used in the experiments (Supplementary Figs. S13, S21–S23). Molecular weights (MW) and isoelectric points (pI) of the amino acid sequences were calculated with the ExPasy tool online (https://web.expasy.org/protparam). Without the stop codons, MtFAAH1 had a MW of 66.02 kDa and a pI of 5.83 whereas MtFAAH2a had a MW of 66.71 kDa and pI of 8.48. Gel bands were analyzed and compared to known standards (Precision Plus Protein Standards, Bio-Rad) in reference to the calculated molecular weights of the FAAHs. Uncropped/unedited SDS-PAGE gels used for Fig. 6b,d can be found in Supplementary Figs. S19, and S20, respectively. The uncropped/unedited SDS-PAGE gels used for Supplementary Fig. S13 can be found in Supplementary Figs. S21–S23 whereas the original gels used for Supplementary Figs. S14–S16 can be found in Supplementary Figs. S24–S26.

### GCMS-based assays for MtFAAH amidohydrolase activity

GCMS- based enzyme activity assays were conducted as described elsewhere^12^ to confirm substrates and reaction products, with a few modifications. Briefly, reaction buffer 1 (50 mM Bis–Tris Propane; pH = 9.0; 0.03% DDM) or reaction buffer 2 (50 mM Tris–HCl, 500 mM NaCl; pH = 7.5; 0.06% DDM) were mixed with 100 µM (final concentration diluted from 10 mM stock solution in DMSO) of NAE12:0. Then, 5 μg of recombinant MtFAAH1 or MtFAAH2a were added into reaction mixtures 1 or 2, respectively. Reactions with 1 μg of AtFAAH in reaction mixture 1 were included as positive control. The reaction mixture was incubated at 30 °C with shaking (120 RPM) for 1 h. Parallel reactions with boiled enzyme (15–30 min at 80–100 °C) were included as negative controls. Reactions were stopped by adding pre-heated isopropanol (IPA), followed by incubation at 70 °C for 30 min. Next, a chloroform-based extraction method was carried out to isolate the lipids generated from the reactions. Lipid products were dried under N_2_ and re-suspended in 50 µL of BSTFA for derivatization (30 min at 50 °C). Then, samples were dried under N_2_ and re-suspended in 70 µL of hexane. Samples were analyzed by GCMS (model Agilent 7693) under the following conditions; pulsed-split-less (injection mode) with an internal pressure and purge flow of 48 (kPa) and 50 (mL/min), respectively. Helium was the mobile carrier phase. The stationary phase was a capillary column (Agilent HP-5 ms GC column) with a length of 30 mm, an internal diameter of 0.25 mm and a film-thickness of 0.25 μM. The GCMS was adjusted for electron impact (EI) MS module and mass spectral data were collected in full-scan mode. Reaction products were identified as trymethylsilyl (TMS)-derivatives. Finally, identification and quantification of the lipid species from the reactions were performed by analysis of their peak retention times, corresponding mass spectra, TMS-ion diagnostic species and/or NIST (National Institute of Standards and Technology) library search.

### Fluorescamine-based assays for MtFAAH amidohydrolase activity

Fluorescamine-based enzyme activity assays were conducted as developed and described elsewhere^22,36^, with few modifications. For the enzyme activity assays at different pH conditions, recombinant MtFAAH1 or MtFAAH2a (1 μg) were incubated with 100 µM of NAE12:0 in reaction buffer 1 (100 mM K-phosphate, 6 mM of K_2_-phosphate; pH = 6), reaction buffer 2 (25 mM HEPES, 100 mM NaCl; pH = 7), reaction buffer 3 (25 mM HEPES, 100 mM NaCl; pH = 8), or reaction buffer 4 (25 mM Tris–HCl, 10 mM MgCl_2_; pH = 9) in a 96-well multitier plate for 5–10 min at 30 °C. DDM (0.03% or 0.06%) were added into each reaction with MtFAAH1 or MtFAAH2a, respectively. Substrates were added from stock solutions to desired final concentrations to start the reactions. Parallel reactions with boiled enzyme (30 min at 80–100 °C) were included as negative controls. Three independent reactions (replicates) were carried out in the experiments. Reactions were stopped by adding 20 µL of phenylmethylsulfonyl fluoride (PMSF) (10 mM stock in DMSO). An aliquot of 15 μL was mixed with 45 μL of fluorescamine (3.6 mM stock in acetone) and 97.5 μL of milliq water in a 96-well microplate for fluorescence-based assays. Reaction mixtures were incubated for 5 min at room temperature. Fluorescence was measured in a BioTek Synergy 2 Multi-Mode Microplate Reader set at 390 nm (excitation) and 475 nm (emission). Raw fluorescent values from the reactions were subtracted from the negative control readings. Then, to transform fluorescent values into µmol of ethanolamine produced, standard curves were built separately with varying concentrations of ethanolamine (Sigma-Aldrich) reacted with fluorescamine. Enzyme activity assays at five different temperature conditions (20, 30, 40, 50, or 80 °C) were also carried out as described above.

For the purification table (Supplementary Table S7), cell lysate, IMAC or SEC purified fractions were used as enzyme sources for enzyme activity testing against NAE12:0, following the strategy stated above. Total activity is represented as an enzyme unit (U), and this represents the μmol of product (ethanolamine) generated per unit of time (min). Specific activity was calculated by dividing total activity by the amount (mg) of protein utilized in the assays. Yield is represented as percentage values, and it represents the enzyme activity that is retained following each purification step. The yield of IMAC or SEC fractions was calculated by dividing the total activity of each of those steps by the total activity derived from the crude cell lysate. Purification level was calculated as a ratio between the specific activity of IMAC or SEC fractions and the specific activity calculated for the crude cell lysate. The average of triplicate independent reactions is presented in Supplementary Table S7.

For the enzyme kinetic assays, recombinant MtFAAH1 or MtFAAH2a (1 μg) were incubated with increasing concentrations of NAE or NAE-like substrates (5–100 μM) in reaction buffer (25 mM HEPES, 100 mM NaCl) at pH = 9 and 0.03% DDM for MtFAAH1 and at pH = 7.5 and 0.06% DDM for MtFAAH2a. Reaction mix was incubated for 5–10 min at 30 °C. Parallel reactions with boiled enzyme (30 min at 100 °C) were included as negative controls. Three independent reactions (technical replicates) were carried out in the experiments. Substrates included; NAE12:0 (synthesized in house), NAE18:2 (Cayman Chemical), NAE-9-HOD (synthesized in house, as reported elsewhere^23^), OdDHL (Sigma-Aldrich), OtDHL (Sigma-Aldrich), *p*-coumaryl-HL (Sigma-Aldrich), *p*-coumaryltyramine (gift from Dr. David Mackey), and affinin (gift from Dr. Jorge Molina Torres). Reactions were stopped by adding 20 µL of phenylmethylsulfonyl fluoride (PMSF) (10 mM in DMSO). An aliquot of the reaction was mixed with fluorescamine and water as described above. The mix was incubated for 5 min at room temperature. Fluorescence was measured as described above.

To transform fluorescent values into µmol of amine produced, standard curves were built with increasing concentrations of ethanolamine (Sigma-Aldrich) (for NAEs), homoserine lactone (Sigma-Aldrich) (for AHLs and *p*-coumaryl-HL), tyramine (Sigma-Aldrich) (for *p*-coumaryltyramine), or isobutyl amine (for affinin) (Sigma-Aldrich) standards. Apparent enzyme kinetic parameters were calculated in GraphPad Prism 8.0. Fitted curves had R^2^ values from 0.54 to 0.94 for MtFAAH1 and from 0.78 to 0.94 for MtFAAH2a.

## Supplementary Information

Supplementary Information 1.

Supplementary Video 1.

Supplementary Video 2.

Supplementary Video 3.

Supplementary Video 4.

Supplementary Video 5.

Supplementary Video 6.

Supplementary Video 7.

Supplementary Video 8.

Supplementary Video 9.

Supplementary Video 10.

Supplementary Video 11.

Supplementary Video 12.

## Data Availability

The mass spectrometry proteomics data generated and analyzed during the current study are available via ProteomeXchange with identifier PXD038494.
