# Supplementary material for: Two legume fatty acid amide hydrolase isoforms with distinct preferences for microbial- and plant-derived acylamides
Source: Sci Rep. 2023 May 9;13:7486. doi: 10.1038/s41598-023-34754-z (PMC10169808; doi:10.1038/s41598-023-34754-z)
Supplement: Supplementary file 1 — Supplementary Information 1. [file 41598_2023_34754_MOESM1_ESM.pdf]

Supplementary Information for

Two Legume Fatty Acid Amide Hydrolase Isoforms with Distinct Preferences for Microbial- and Plant-Derived Acylamides

Omar Arias-Gaguancela, Emily Herrell, Mina Aziz, & Kent D. Chapman*

*Author for correspondence; chapman@unt.edu

This PDF file includes:

Supplementary Figures S1 to S26
Supplementary Tables S1 to S8
Legends for Supplementary Videos S1 to S12

Other supporting materials for this manuscript include:

Supplementary Videos S1 to S12

```
AtFAAH          ----MGKYQVMKRASEVDLST---VKYKAETMKAPHLTGLSFKLFVNLLEAPLIGSLIVD      53
MtFAAH1         ----MGKKRVMVPAKDVDLSS---IKYEPEIVQAPHLTGFWFRFFVRLIEAPLIGPFLLT      53
MtFAAH2a        MGLFKSKRVVYKPVKDVNLGPDSTEFYLQANVKAPRMTGILVKIVAWLLECRIIGAFLLY      60
MtFAAH2b        MSLFKRKCVVYTPANDVDLSPYSTEFYLQANVKAPRMTGILVKIFTYLLELPIIGTILLY      60
                      *  *   ..:*:*.      *    ::**::**: .::.. *:*  :** :::

AtFAAH          YLKKDNGMTKIFRNTVIPEEPMFRPEFPSQ-EPEHDVVIVGEDESPIDRLETALKCLPQY      112
MtFAAH1         MLKKENKIDQLLRNTVFPEEPMFKPEYPPQ-EKEHSVVELDEDGRPEGRVESALNCLPHY      112
MtFAAH2a        ILKGNNLIHKYITNADIEEPPLYVPLHHFEDHKEQEVKSLDLALTPPDKVQVAIDCLPTT      120
MtFAAH2b        ILLKKNLIHELITNAELEESPLYVPLHDFEDIEEKDVKFIDPSSSPPEKVQHAIDCLPIS      120
                 *  .* : : : *: : * *:: * .  :   *:.*  :.    *  ::: *:.***

AtFAAH          DPSRSLHADPVSSFRYWKIRDYAYAYRSKLTTPLQVAKRIISIIEEFGYDKPPTPFLIRF      172
MtFAAH1         DPAKLW-ENSSATFRYWKIRDYAYAYQSRKVTPSMVAESIISMIEENGIDKPPTPLLLSF      171
MtFAAH2a        LQRPI--NGTKPSFNRWTIMDYFRAYSSGDITPHMVAERFIAAVDESSKPTLQMGFFINY      178
MtFAAH2b        VGKKP--NGTNP-FCRWTIMDYSKAYRSGDITPRLVAESFVAAIDESIKPPLQMGFFIHY      177
                        .    *  *.* **  ** *   **  **: ::: ::*         ::: :

AtFAAH          DANEVIKQAEASTRRFEQGNPISVLDGIFVTIKDDIDCLPHPTNGGTTWLHEDRSVEKDS      232
MtFAAH1         DAAEVRKQAAASTQRFESGNPLSILDGIFIAIKDDIDCHPHPSTGGSTWMHEVRDVKKDA      231
MtFAAH2a        NVEDILRQANESTLRYQKGEPISVLDGVPVAIKDEIDCLPYPTTGGTKWLHKQRPCTDDA      238
MtFAAH2b        NVDDILRQATESTLRYQRGEPISVLDGVPVAIKDEIDCLPYPTTGGTKWLHKERPCKDDA      237
                :. :: :**  ** *:: *:*:*:***: ::***:*** *:*:.**:.*:*: *   .*:

AtFAAH          AVVSKLRSCGAILLGKANMHELGMGTTGNNSNYGTTRNPHDPKRYTGGSSSGSAAIVAAG      292
MtFAAH1         VCVSRLRSCGVIFIGKTNMHEFGMGTTGNNSNYGTARNPHAPDRYTGGSSSGPAAIVASG      291
MtFAAH2a        CCIKRLRLCGAVLVGKTNMHELGAGTSGINPHYGATRNPHNASRIAGGSSSGSAAVVSAG      298
MtFAAH2b        CCVKRLRQCGTILVGKSNMHELGSGTSGINPHYGPTRNPYDCKKIAGGSSGGSASLVSAG      297
                  :.:** **.:::**:****:* **:* * :** :***:  .: :****.* *::*::*

AtFAAH          LCSAALGTDGGGSVRIPSALCGITGLKTTYGRTDMTGSLCEGGTVEIIGPLASSLEDAFL      352
MtFAAH1         LCSAALGTDGGGSVRIPSSLCGVVGLKINYGRTSMEGSLCDSGTVEVIGPIASTVEDAML      351
MtFAAH2a        LCPVALGVDGGGSVRMPAALCGIVGLKPTFSRIPHSGVIPLNWTVGMVGILAGTVEDSLI      358
MtFAAH2b        LCPVALGVDGGGSVRMPAALCGVVGLKPTFARIPHDGVLPINWTVGMVGILAGTVEDAMI      357
                ** .***.*******:*::***:.*** .:.*    * :  . ** ::* :*.::**:::

AtFAAH          VYAAILGSSSADRYNLKPSPPCFPKLLSHNGSNAIGSLRLGKYTKWFNDVSSSDISDKCE      412
MtFAAH1         VYAAMLGASPANRISMKPSTPCLPTLSSDDDTDALRSLRIGIYTPWFNNVHSTEVSDKCE      411
MtFAAH2a        TYAAISGEIPSRQPSSIPAKINLPLLPL---TKSISKIKLAKYGKWFDDCS-DDVRICCS      414
MtFAAH2b        VYAALSGEIPSHHPSSVLTKINIPRLSS---TKSISHIRLAKYGKWFDDCS-NDVKVCCS      413
                .***: *   : : .   :   :* *     :.::  :::. *  **::    ::   *.

AtFAAH          DILKLLSNNHGCKVVEIVVPELEEMRAAHVISIGSPTLSSLTPYCEAGKNSKLSYDTRTS      472
MtFAAH1         DALNLLSKAHGCEVVEVVIPEIVEMRTAHLVSIGSECLSSLNPDIEDGKGVKLSYDTRTS      471
MtFAAH2a        GALNKLQGHYGWKIVDVTIPEIEVMRLAHYSTIGSECNTSLDYFQD-KNLADFGWDARVA      473
MtFAAH2b        LALHKLQDHYNWKIIDVTVPEIEVMRLAHYITIGSECSTALDSYKE-KNFAELGWDVRVA      472
                  *: *.  :. :::::.:**:  ** **  :***   ::*    :  :  .:.:*.*.:

AtFAAH          FAIFRSFSASDYIAAQCLRRRLMEYHLNIFKDVDVIVTPTTGMTAPVIPPDALKNGETNI      532
MtFAAH1         LALFQSFTAADYVAAQCIRRRIMHYFMEIFKKVDVIVTPTTGMTAPRIPPSALKSGETDM      531
MtFAAH2a        LKIYGSFSSMEYIKAQKIRNRQLQFHKKIFSEADIIVSPTTGVTAYPIQDDALKTGELDY      533
MtFAAH2b        QSIYGAFSGIEYVKAQRIRNRQLQFHKKIFSEADVIVSPTTGVTAYPIQDDALKTGELDY      532
                  :: :*:. :*: ** :*.* :.:. :**...*:**:****:**  *  .***.** :

AtFAAH          QVTTDLMRFVLAANLLGFPAISVPVGYDKEGLPIGLQIMGRPWAEATVLGLAAAVEELAP      592
MtFAAH1         PTTGYLMRFVVPANLLGLPAISVPVGYDKEGLPIGLQVIGRPWAEATILRVAAAVEKLCG      591
MtFAAH2a        VNGAALIRYSIAGNFLGLPAVTVPVGYDKLGLPIGLQFIGRPWAEATLIHLAFAMQTICL      593
MtFAAH2b        VNGAALVRYSIAGNFLGLPAVTVPVGYDKFGLPIGLQFIGRPWSEATLIHLAFAMQAICM      592
                     *:*: : .*:**:**::******* *******.:****:***:: :* *:: :.

AtFAAH          VT-KKPAIFYDILNTN  607
MtFAAH1         ESKRRPVTYYDVLGAN  607
MtFAAH2a        SEYRKPAIFYDLLRTN  609
MtFAAH2b        PDYRKPELYYDLLRR-  607
                   ::*  :**:*
```

Figure S1. Clustal Omega alignment between AtFAAH, MtFAAH1, MtFAAH2a, and MtFAAH2b amino acid sequences.

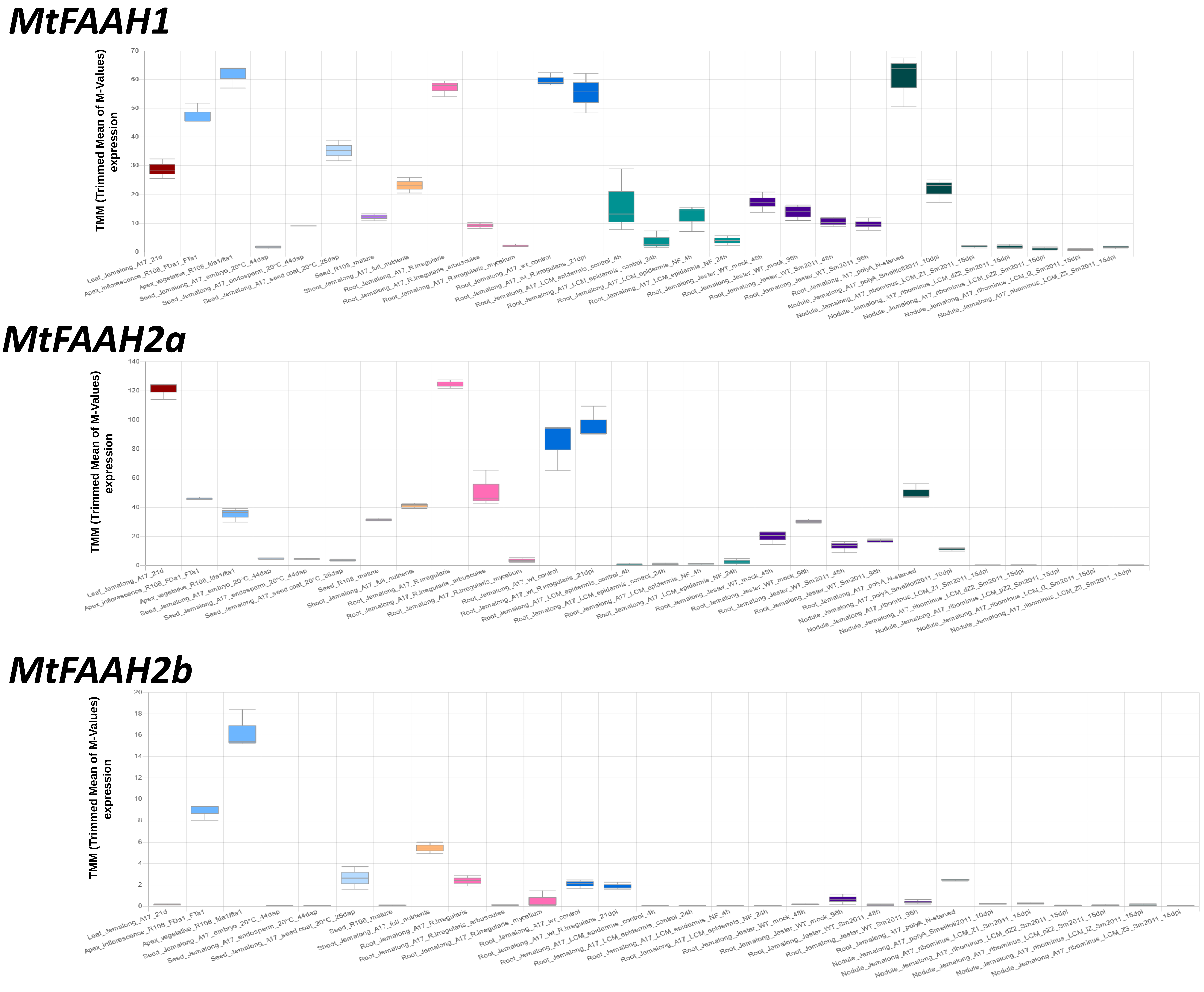

Figure S2. Transcript expression profiles of *MtFAAH1*, *MtFAAH2a* and *MtFAAH2b* obtained from the *Medicago truncatula* Gene Expression Atlas "MtExpress". Legend titles "TMM (Trimmed Mean of M-Values) expression" have been added to the Y-axis of each profile to specify the units used in the expression profiles.

MtFAAH1

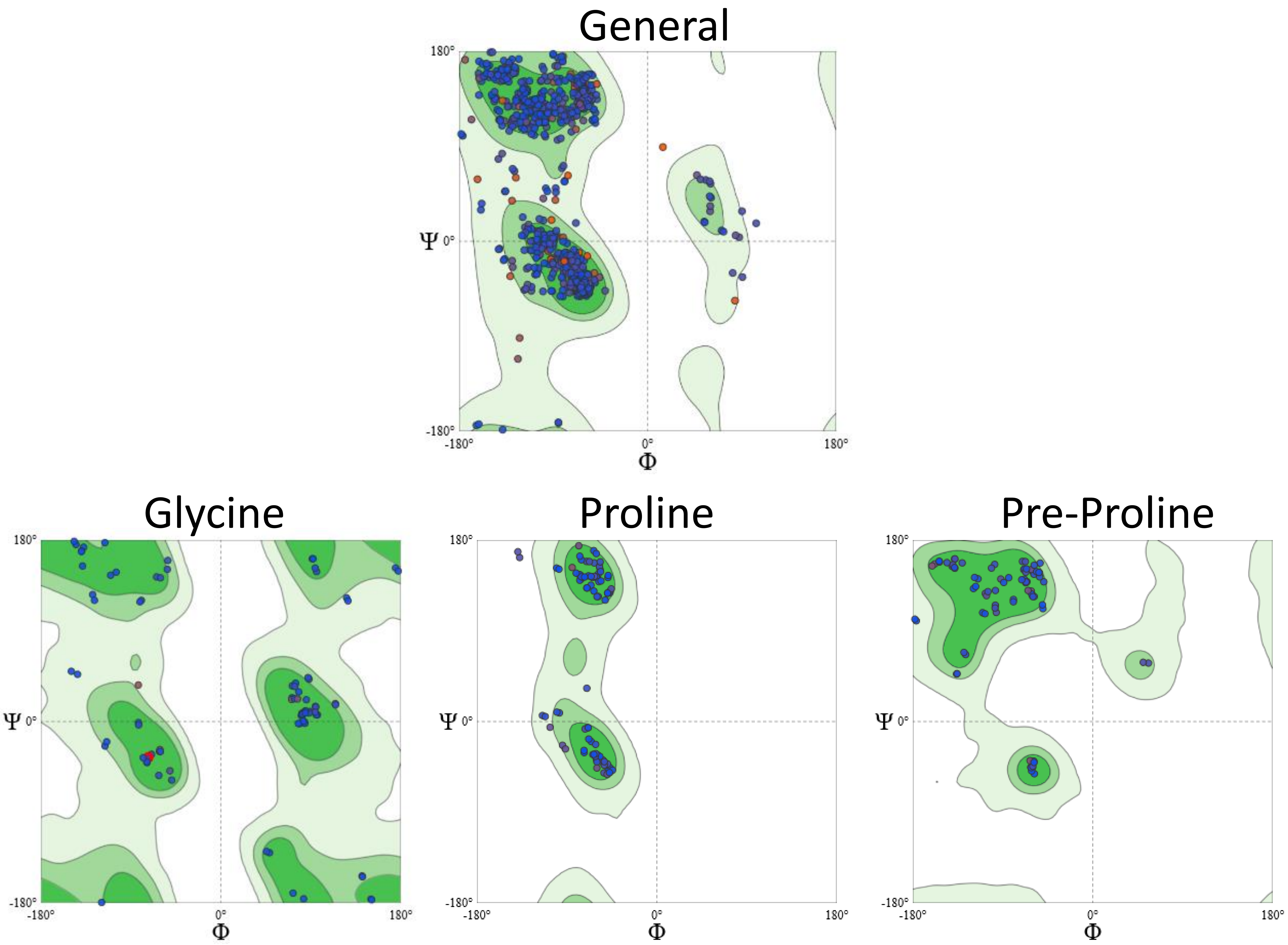

Figure S3. General, glycine, proline and pre-proline Ramachandran derived from MtFAAH1 homology model build with SWISS-MODEL.

MtFAAH2a

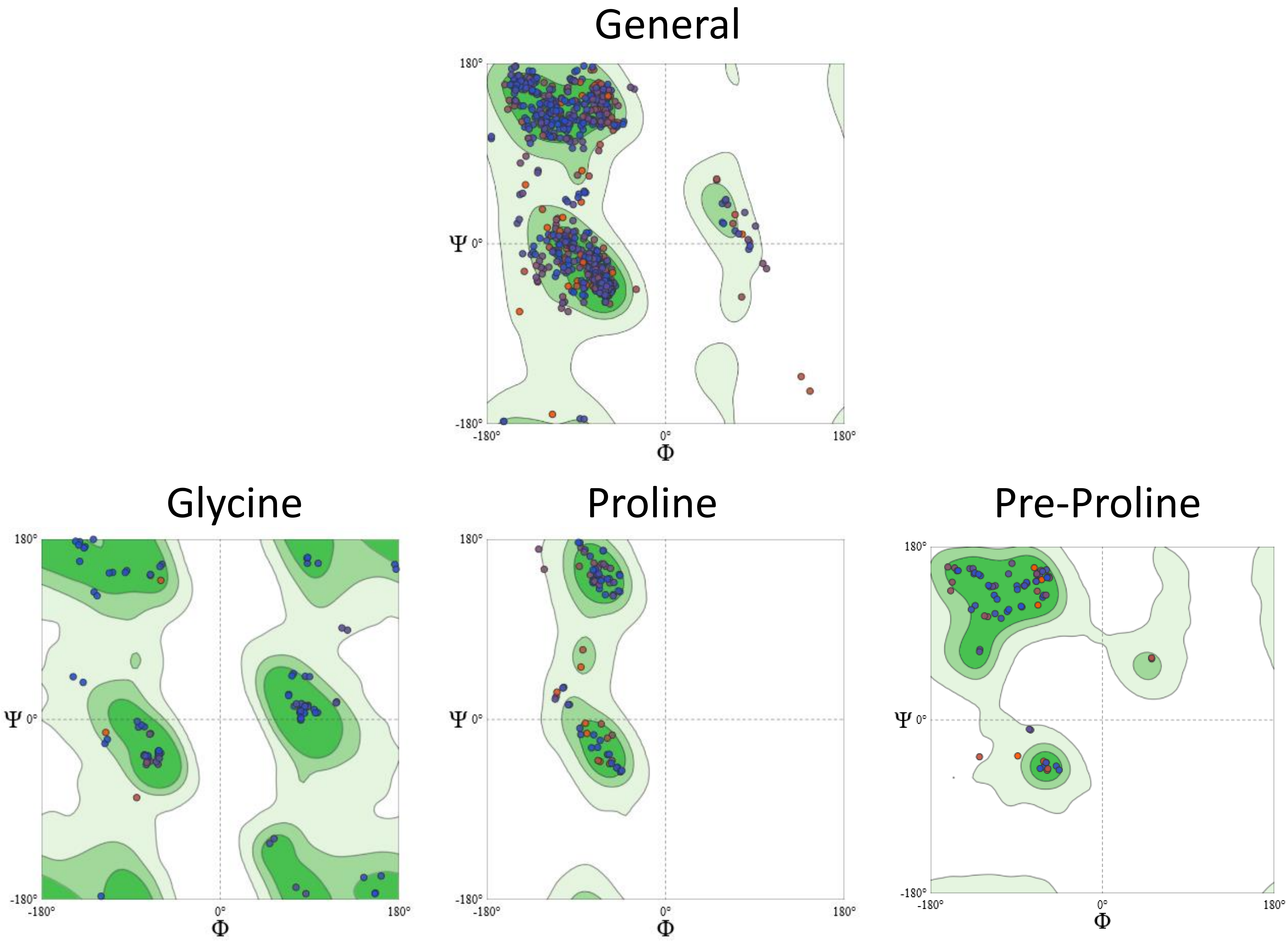

Figure S4. General, glycine, proline and pre-proline Ramachandran derived from MtFAAH2a homology model build with SWISS-MODEL

MtFAAH1 (Subunit A)

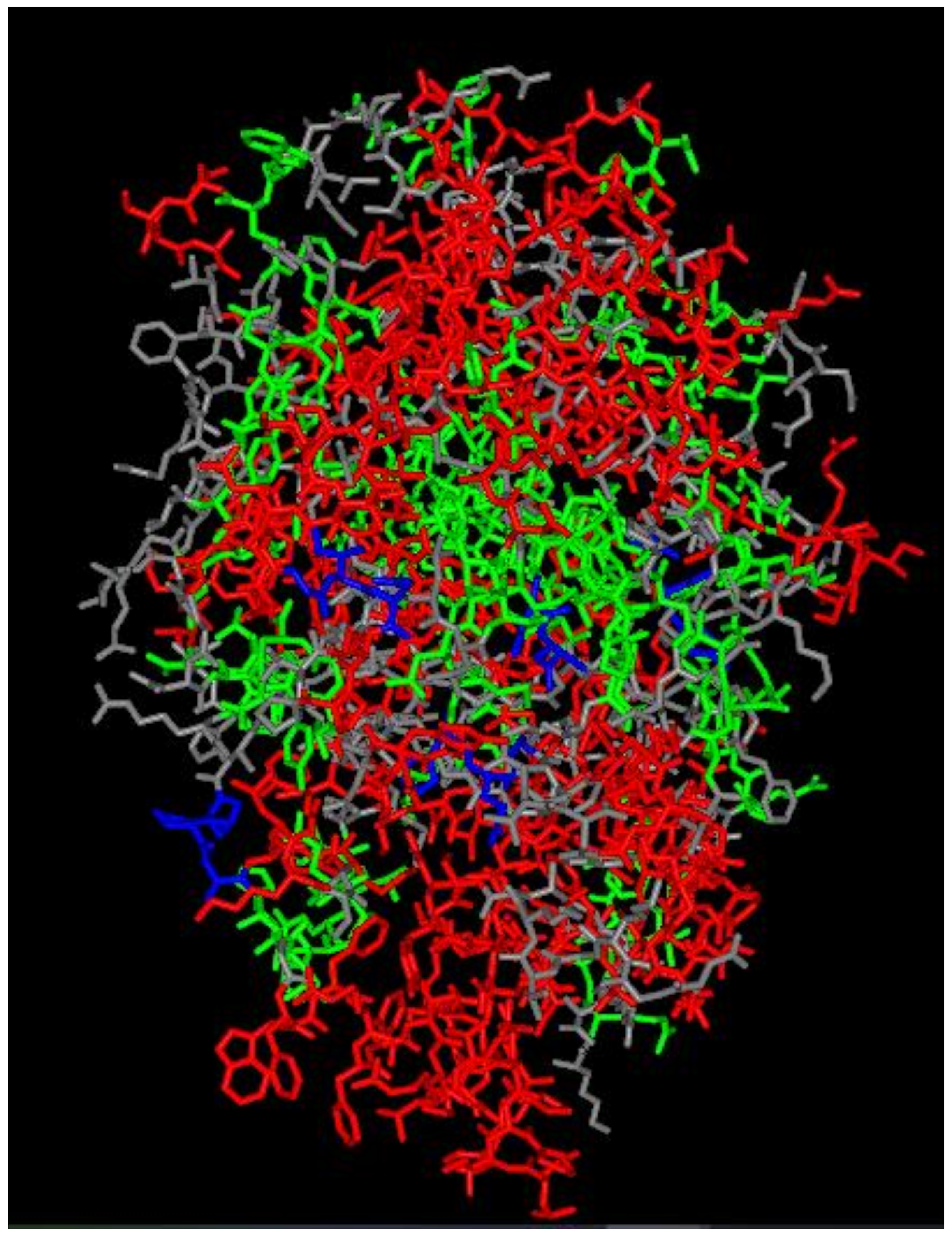

Global secondary structure content
Helix: 45.8%
Sheet: 24.1%
Turn: 2.3%
Coil: 27.7%

Helix (red)
Sheet (green)
Turn (blue)
Coil (grey)

MtFAAH2a (Subunit A)

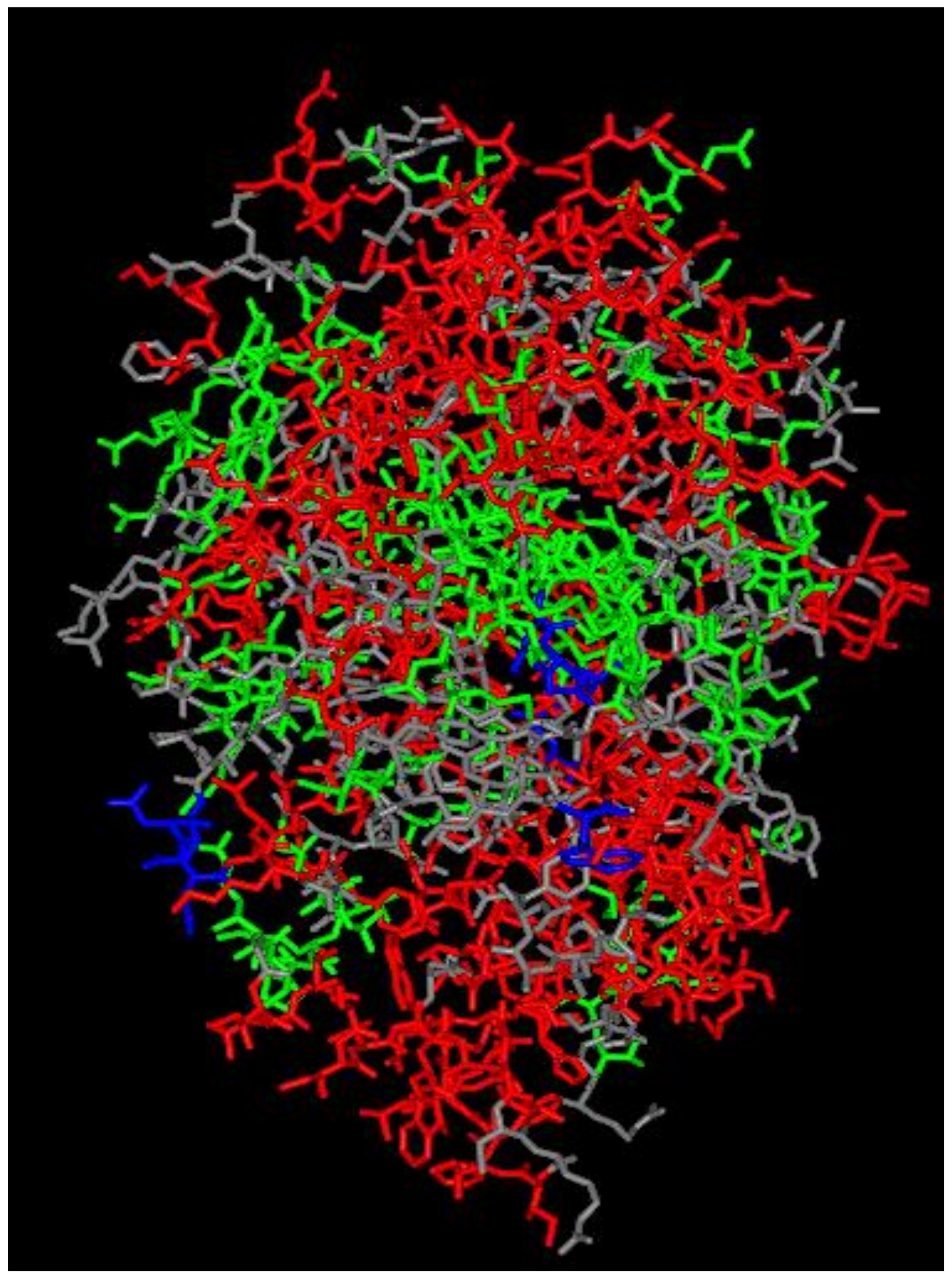

Global secondary structure content
Helix: 45.3%
Sheet: 24.8%
Turn: 1.7%
Coil: 28.2%

Figure S5. Secondary structure content of the subunit A of MtFAAH1 and MtFAAH2a. Values are presented as percentage respect to global content. Antheprot3D was used for content prediction, using the default settings. Color code; red (helix), green (sheet), blue (turn), and grey (coil) sticks.

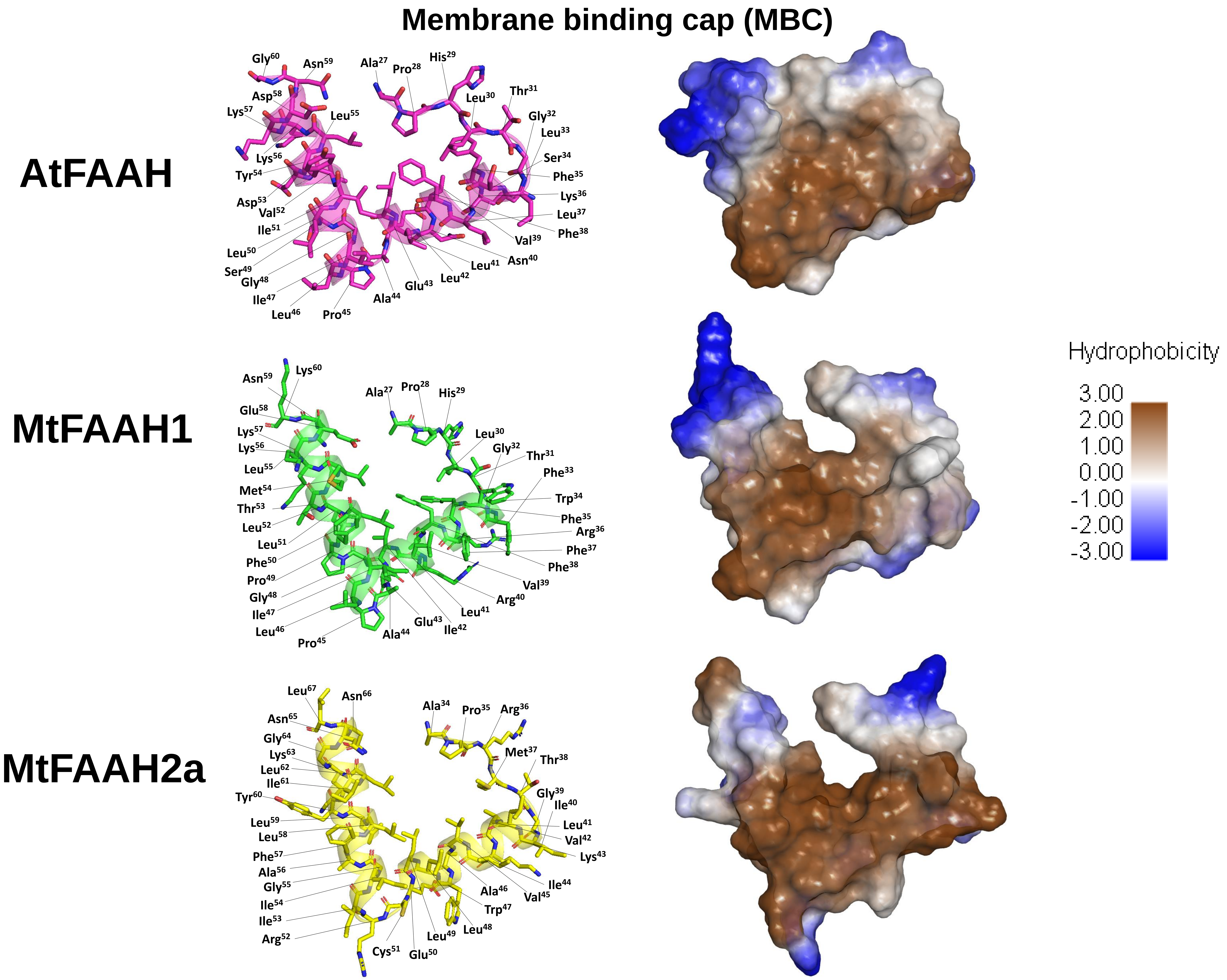

Figure S6. Side-by-side comparisons of the membrane binding caps (MBCs) of *A. thaliana* and *M. truncatula* FAAHs. Left panel; sticks with faded ribbons represent MBCs of AtFAAH (purple), MtFAAH1 (green), and MtFAAH2a (yellow). Right panel; hydrophobicity surface profiles of the same MBCs. Hydrophobicity scale ranges from 3.00 for highest (brown) or -3.00 for lowest (blue) hydrophobic regions.

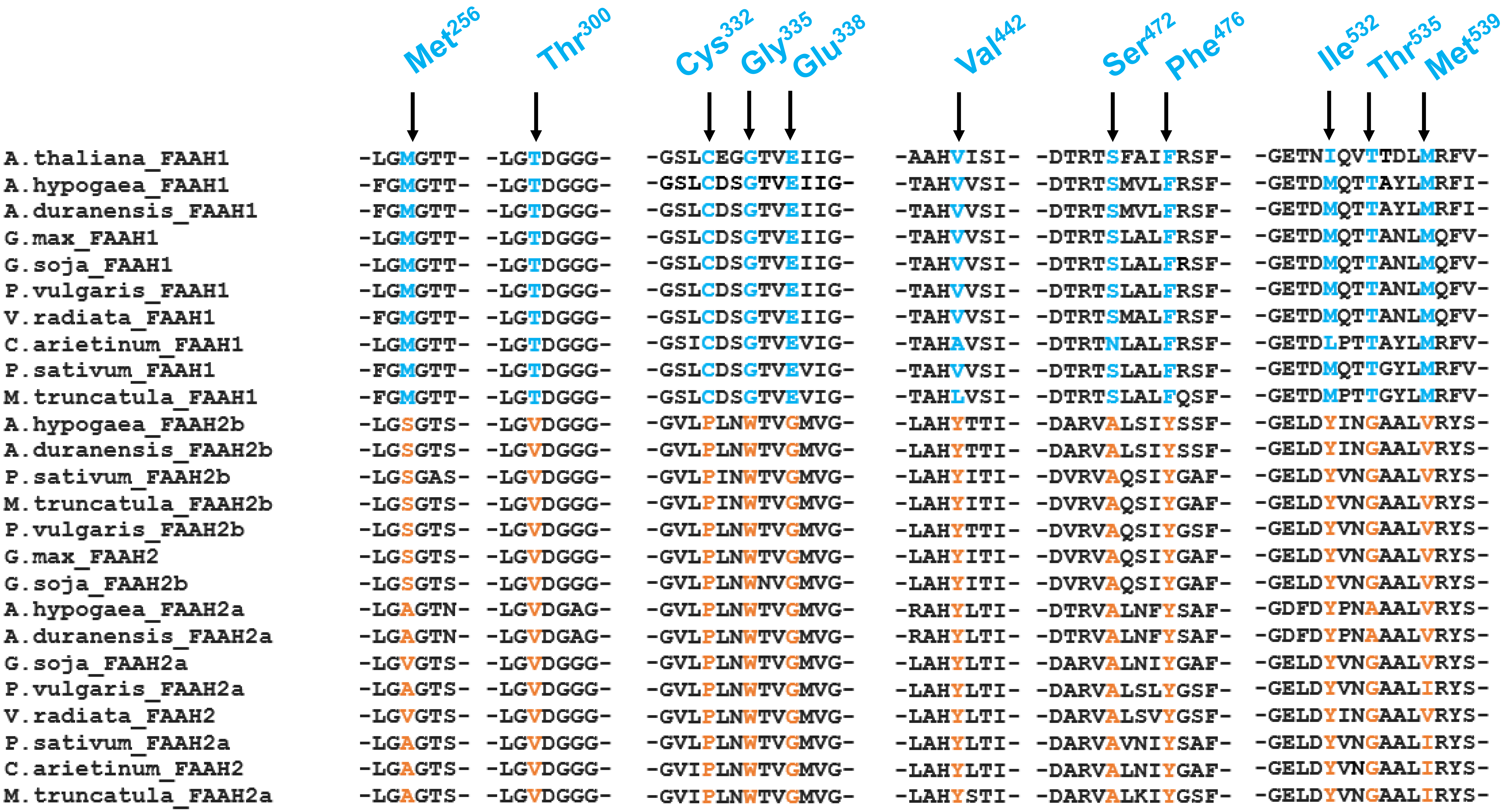

Figure S7. Conserved and key changes in residues of the substrate binding pockets (SBPs) of FAAH1 and FAAH2 groups. Arrows point at selected residues in the SBP that are conserved within FAAH1 or FAAH2 isoforms. Residue numbers of *Arabidopsis thaliana* FAAH are indicated at the top of the alignment as reference. Highlighted in blue and orange fonts are the residues that differ between FAAH1 and FAAH2, respectively.

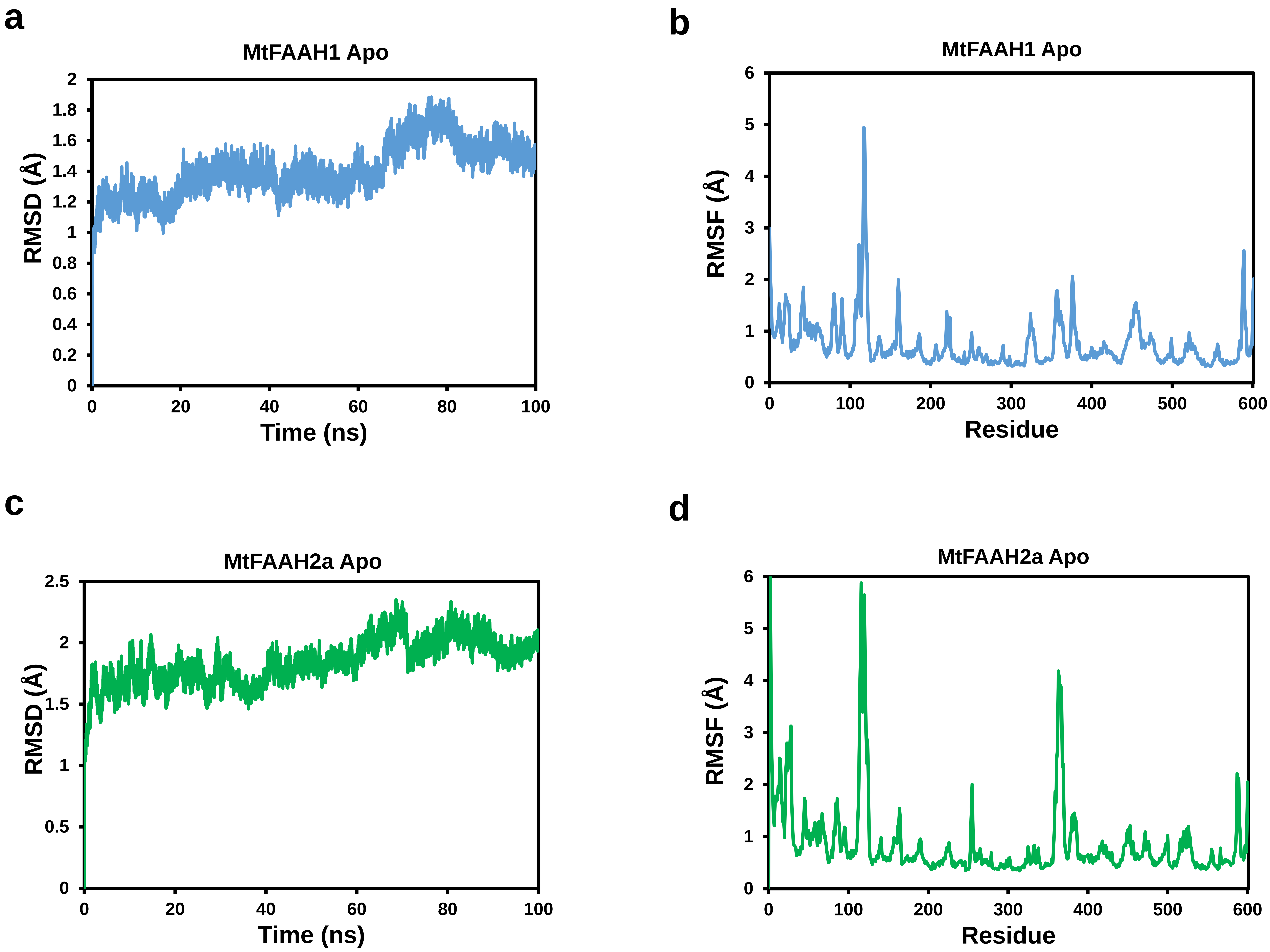

Figure S8. Root-mean-square deviation (RMSD) and root-mean-square fluctuation (RMSF) plots of MtFAAH1 (a, b) (represented as blue lines) and MtFAAH2a (c, d) (represented as green lines) following molecular dynamic simulation (MDS) experiments during 100 ns. Trajectories are represented in Angstroms (Å).

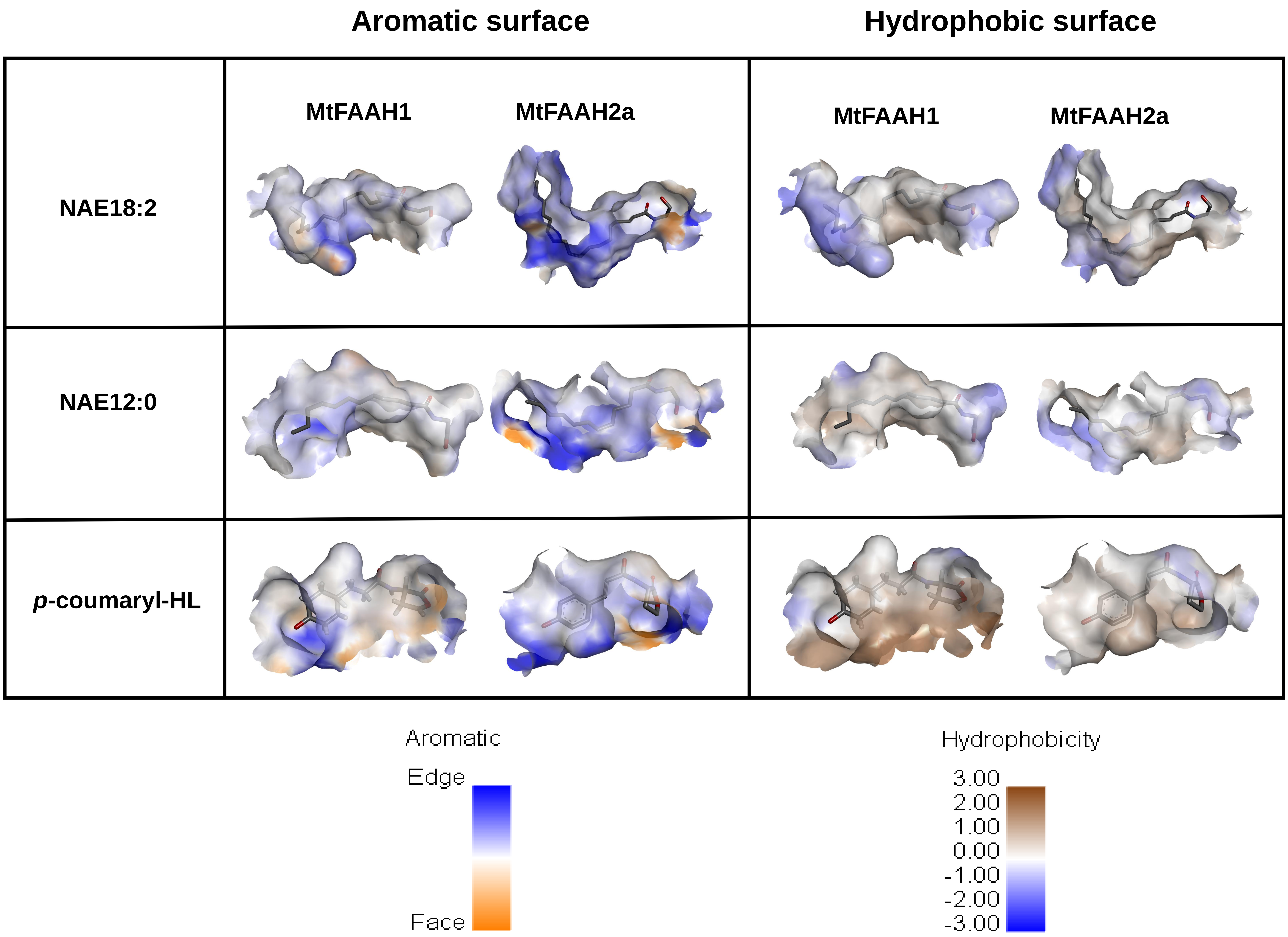

Figure S9. Aromatic and hydrophobic surfaces of MtFAAH1 and MtFAAH2a docked with NAE18:2, NAE12:0 or *p*-coumaryl-HL. The Apo forms of MtFAAH1 and MtFAAH2a were subjected to molecular dynamics simulations during 100 ns prior to the docking experiments. Aromatic scale describes the edge (blue)-to-face (orange) conformations of residues with an aromatic side chain. The hydrophobicity scale ranges from 3.00 for highest (brown) or -3.00 for lowest (blue) hydrophobic regions.

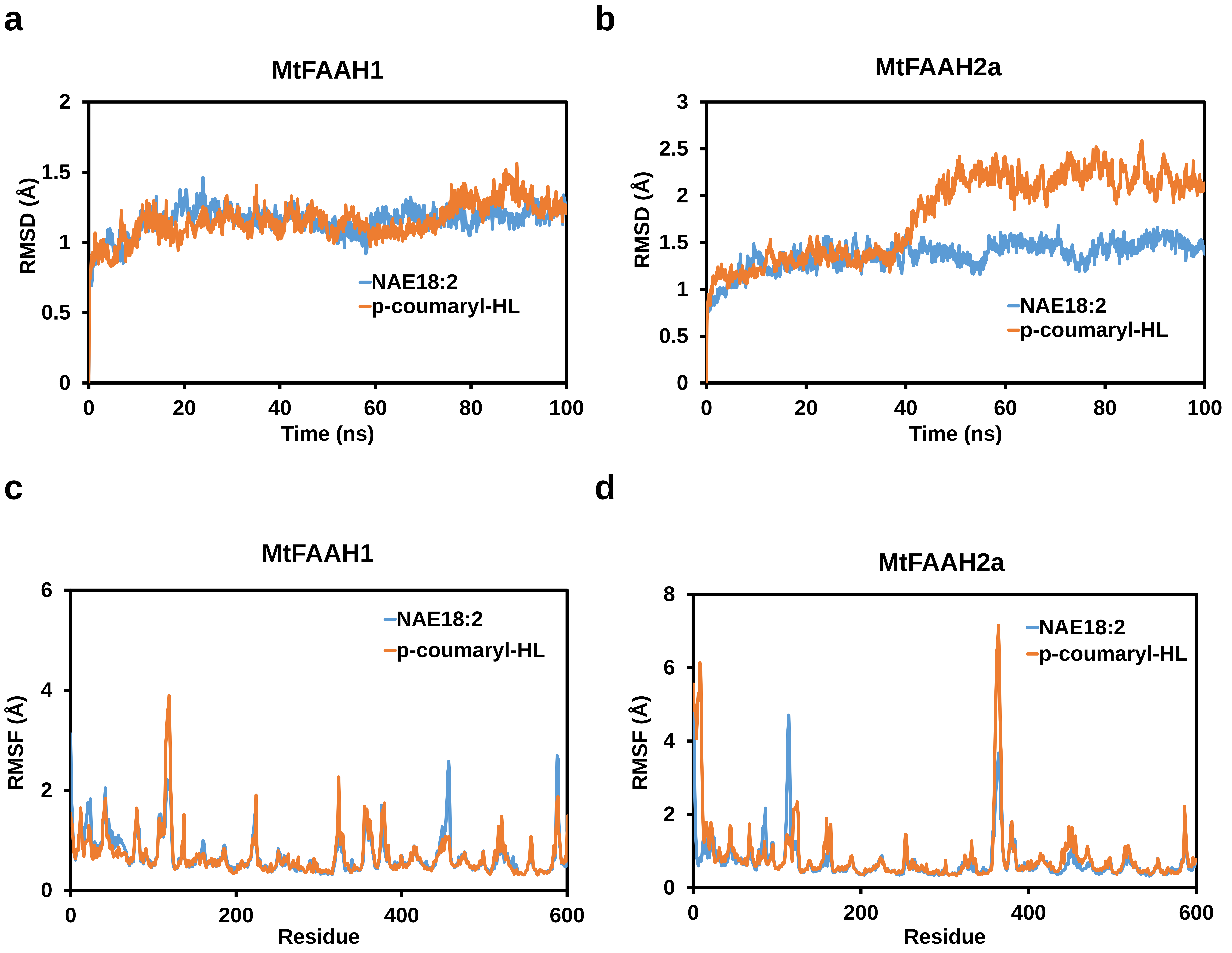

Figure S10. Root-mean-square deviation (RMSD) and root-mean-square fluctuation (RMSF) plots of MtFAAH1 (a, b) and MtFAAH2a (c, d) bound to NAE18:2 (blue lines) or *p*-coumaryl-HL (orange lines) ligands. Molecular dynamic simulation (MDS) experiments were conducted during 100 ns. Trajectories are represented in Angstroms (Å).

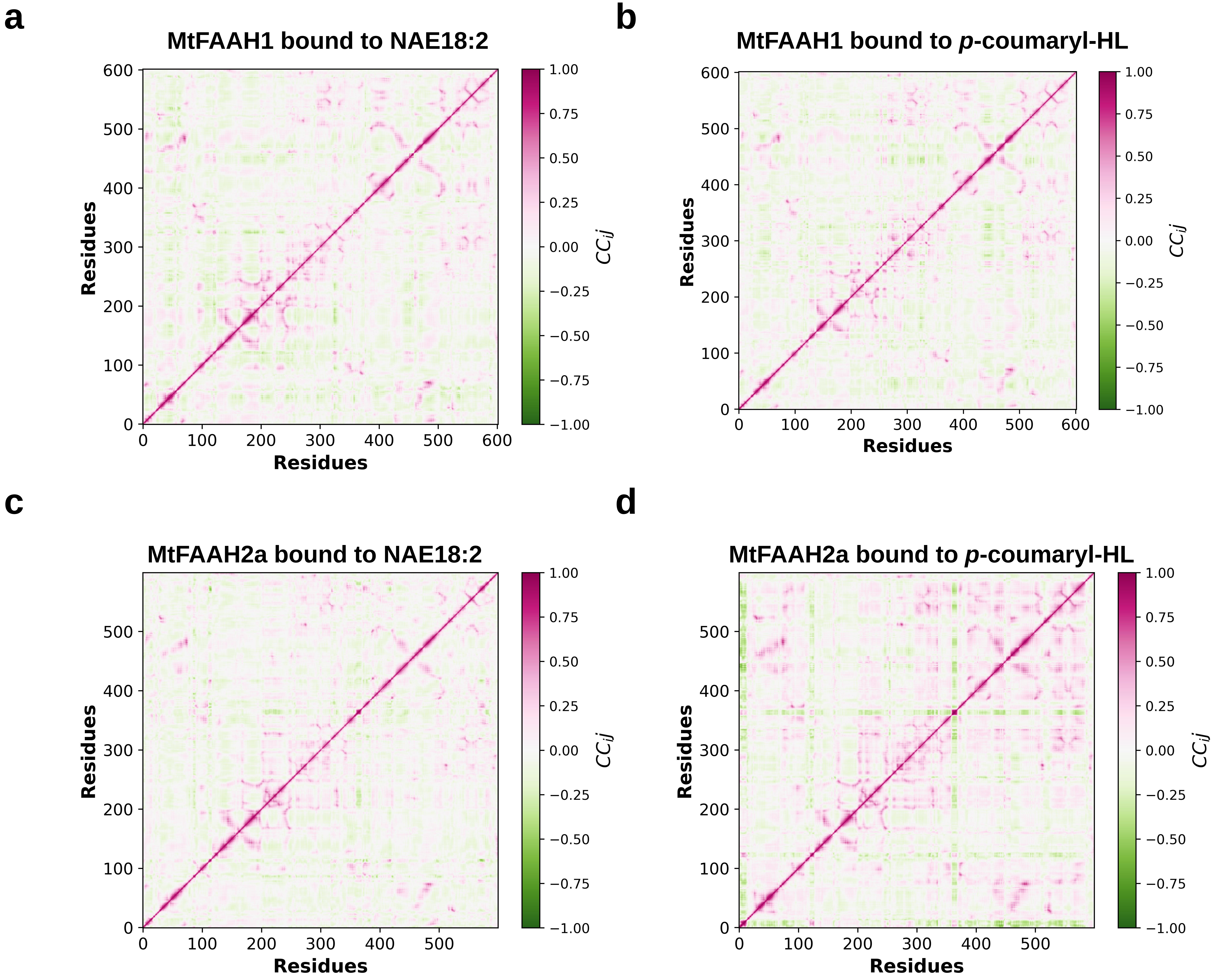

Figure S11. Pearson's cross correlation analysis was generated for MtFAAH1 (a, b) and MtFAAH2a (c, d) bound to NAE18:2 or p-coumaryl-HL.

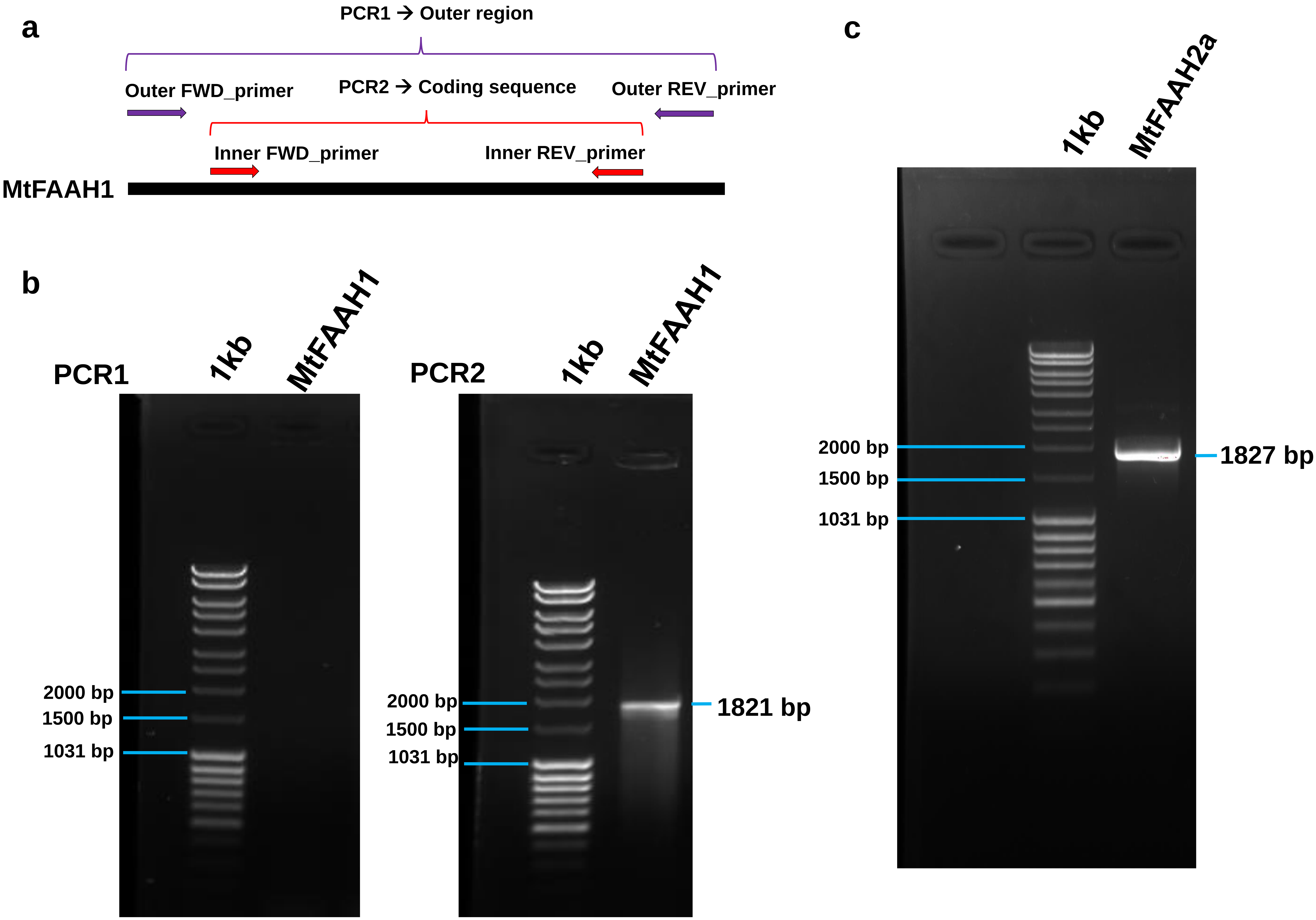

Figure S12. PCR amplification of MtFAAH1 and MtFAAH2a coding sequences. (a) Strategy for MtFAAH1 amplification with nested PCR. Abbreviations: FWD (forward), REV (Reverse). (b) The results of PCR1 with external primers and PCR2 with internal primers for MtFAAH1 are shown. (c) PCR amplification of synthesized MtFAAH2a coding sequence. A 1kb MassRuler DNA ladder was used to estimate PCR product size. The PCR amplicons for MtFAAH1 or MtFAAH2a correspond to their coding sequences without their stop codons.

a

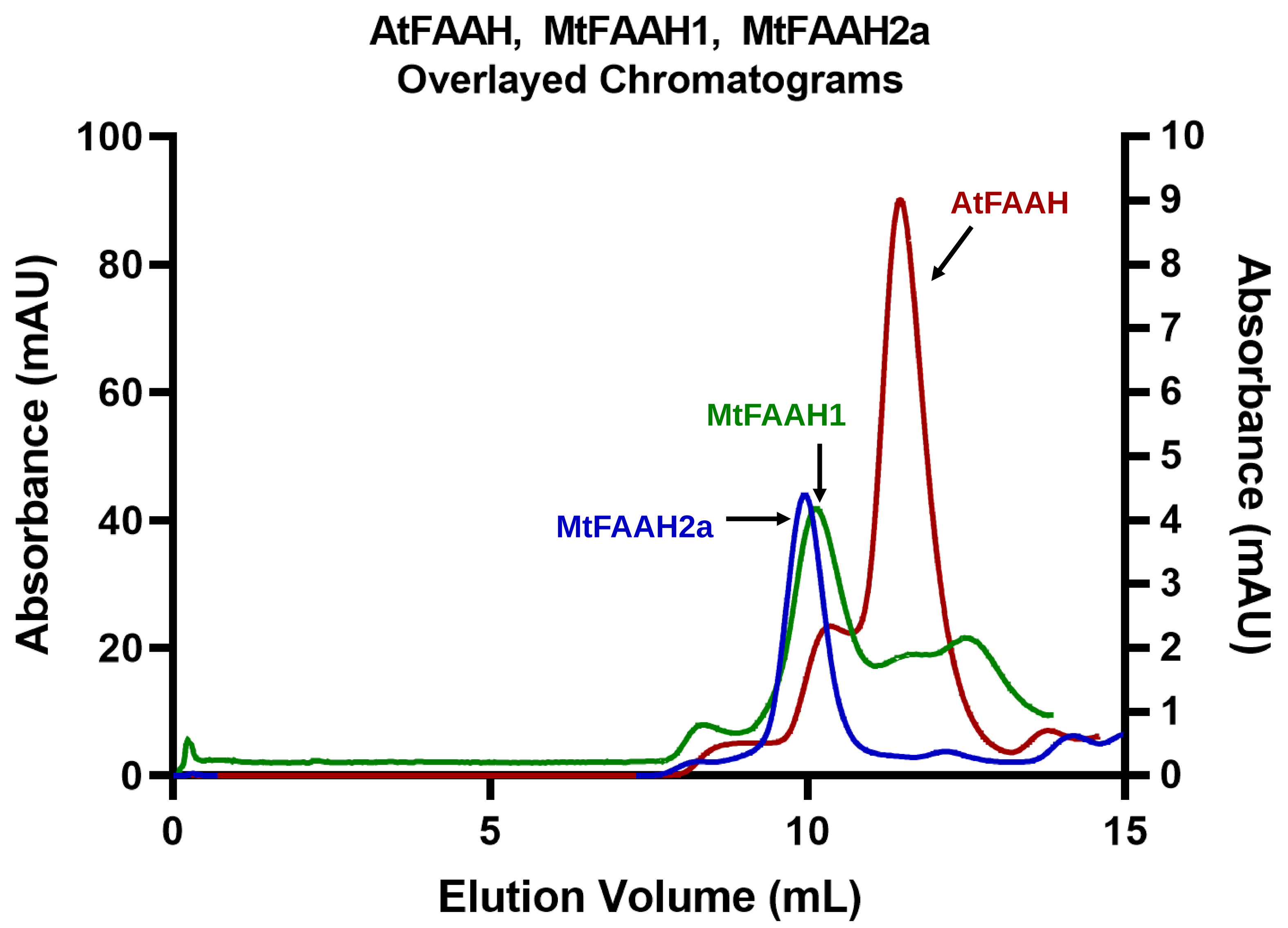

b

Size Exclusion Chromatographic Characterization of FAAH Proteins			
**FAAH**	**Average Elution Volume (mL)**	**Apparent Molecular Weight (kDa)**	**Estimated Subunits in Oligomer**
AtFAAH	11.55±0.09 (s.d.)*	174	2✣
MtFAAH1	10.08±0.14 (s.d.)	346	4
MtFAAH2a	10.14±0.09 (s.d.)	371	4

*n=6

✣=(Apparent Molecular Weight - DDM Molecular Weight) / (Subunit Size)

c

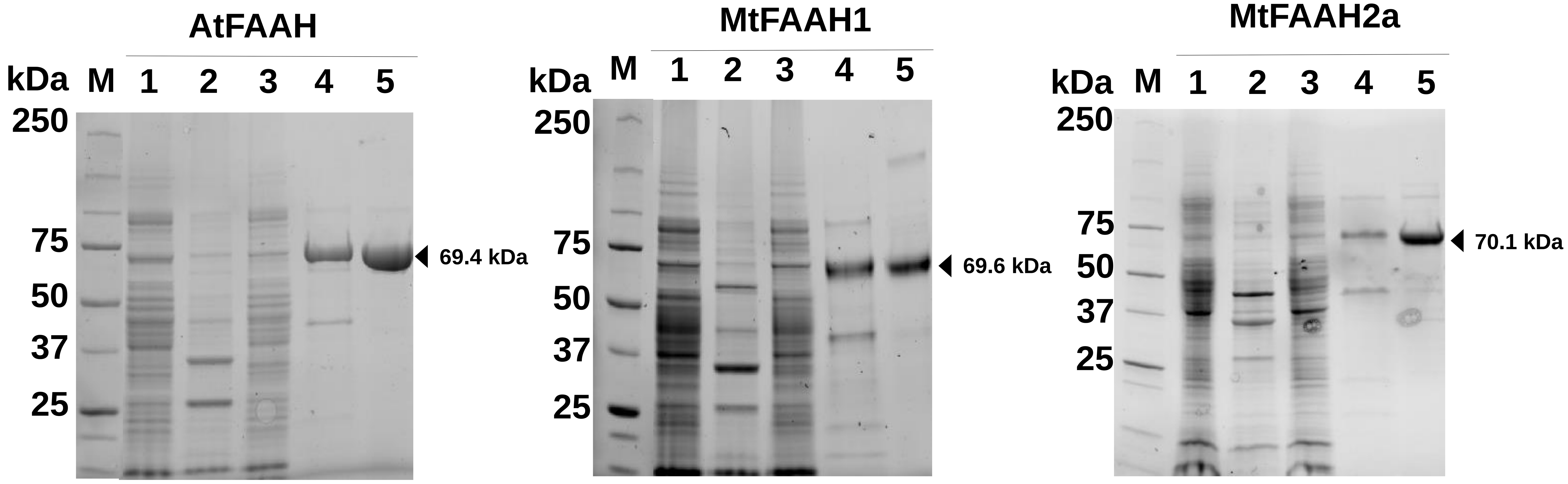

Figure S13. Comparison of AtFAAH, MtFAAH1, and MtFAAH2a molecular weights and oligomeric states. (a) SEC chromatograms of FAAH purifications showing MtFAAH1 and MtFAAH2a eluting as tetramers and AtFAAH eluting as a dimer. Left y-axis corresponds to MtFAAH2a (blue) and AtFAAH (red) peaks. Right y-axis corresponds to MtFAAH1 peak (green) (b) Table showing average elution volume, apparent molecular weight of FAAHs, and estimated subunits in the oligomers. (c) Stain free SDS-PAGE gels of fractions taken during AtFAAH, MtFAAH1, and MtFAAH2a purifications. Molecular weight ladder (lane M), cell lysate of BL21 (DE3) cells after sonication (lane 1), pelleted cell debris after sonication (lane 2), flow through of nickel affinity column (lane 3), nickel affinity purified protein (lane 4), size exclusion chromatography purified protein (lane 5). The uncropped/unedited SDS-PAGE gels used in Supplementary Fig. S13 (panel c) can be found in Supplementary Fig. S21-S23

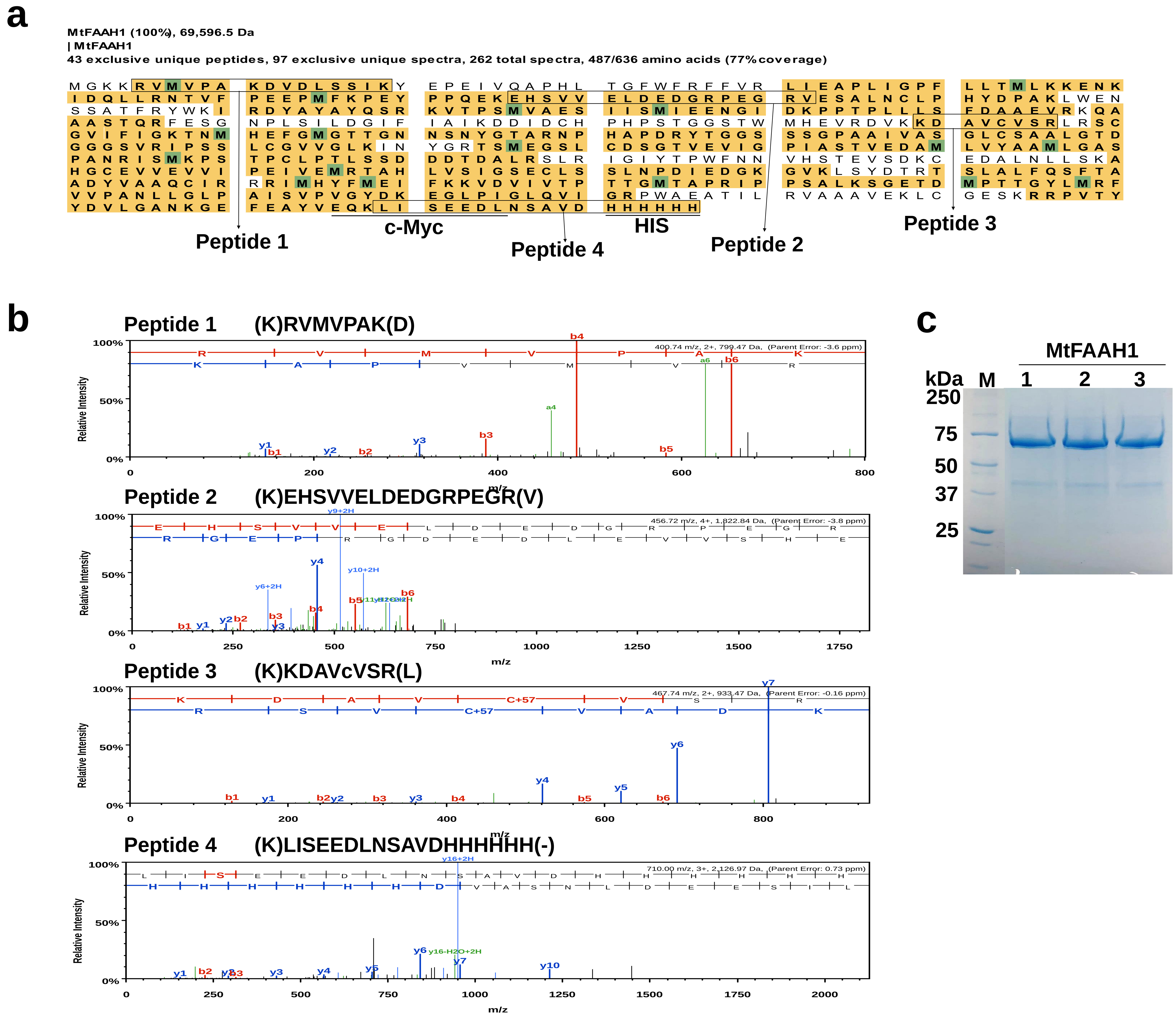

Figure S14. Liquid chromatography with tandem mass spectrometry (LC/MS/MS) of MtFAAH1. (a) Amino acid sequence of MtFAAH1. Identified sequences after digestion are highlighted with the c-Myc and HIS tags labelled. Four regions were selected (boxed) for spectra analysis. (b) Corresponding spectra of the regions selected in panel (a). (c) Coomassie stained SDS-PAGE gel of MtFAAH1 samples purified by a Superdex 200 increase 10/300 GL column. Protein marker was loaded in lane M and identical samples were loaded in lanes 1, 2, and 3. Samples were cut out and analyzed by LC/MS/MS. The uncropped/unedited SDS-PAGE gels used in Supplementary Fig. 14 (panel c) can be found in Supplementary Fig. S24.

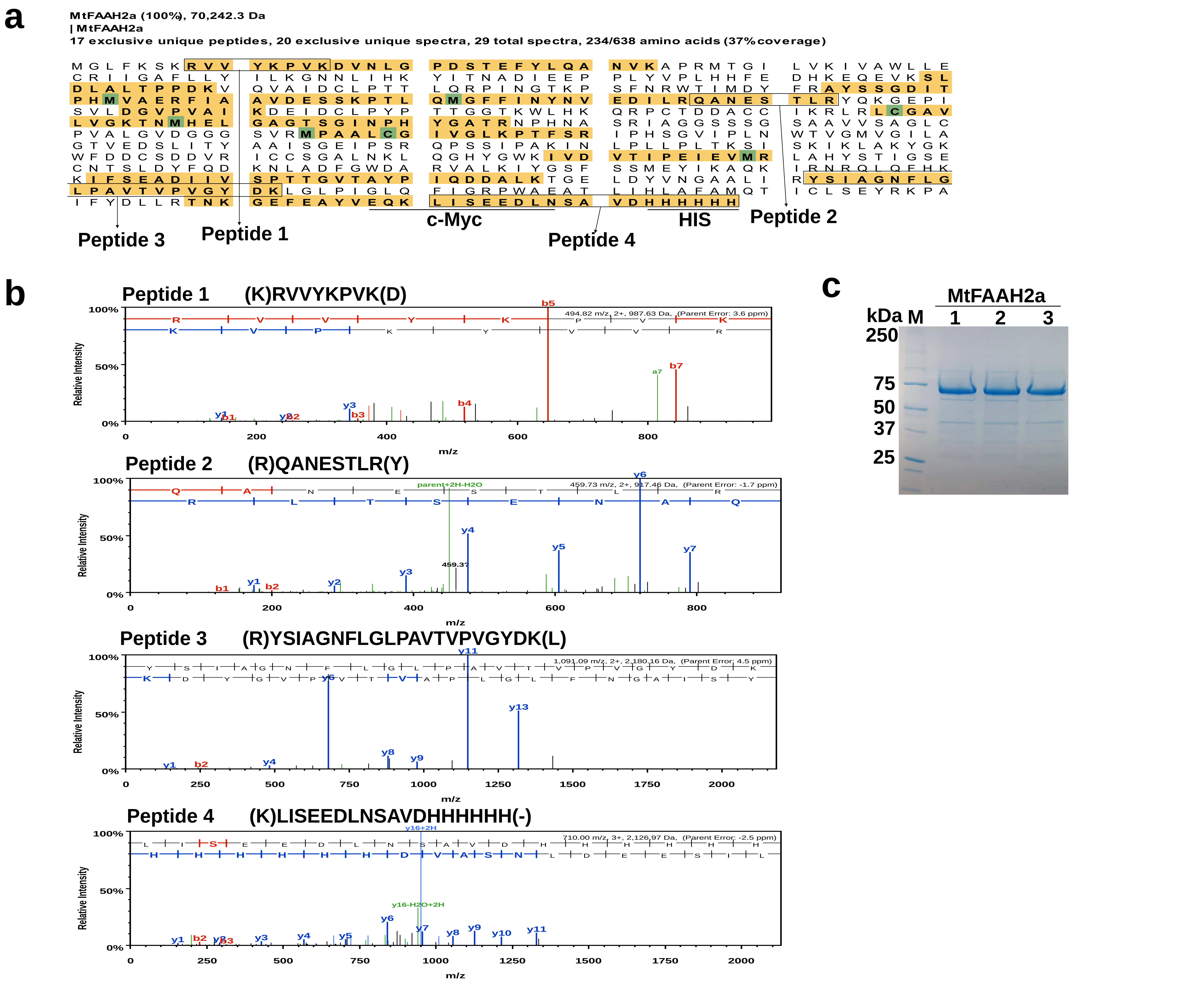

Figure S15. Liquid chromatography with tandem mass spectrometry (LC/MS/MS) of MtFAAH2a. (a) Amino acid sequence of MtFAAH2a. Identified sequences after digestion are highlighted with the c-Myc and HIS tags labelled. Four regions were selected (boxed) for spectra analysis. (b) Corresponding spectra of the regions selected in panel (a). (c) Coomassie stained SDS-PAGE gel of MtFAAH2a samples purified by a Superdex 200 increase 10/300 GL column. Protein marker was loaded in lane M and identical samples were loaded in lanes 1, 2, and 3. Samples were cut out and analyzed by LC/MS/MS. The uncropped/unedited SDS-PAGE gels used in Supplementary Fig. 15 (panel c) can be found in Supplementary Fig. S25.

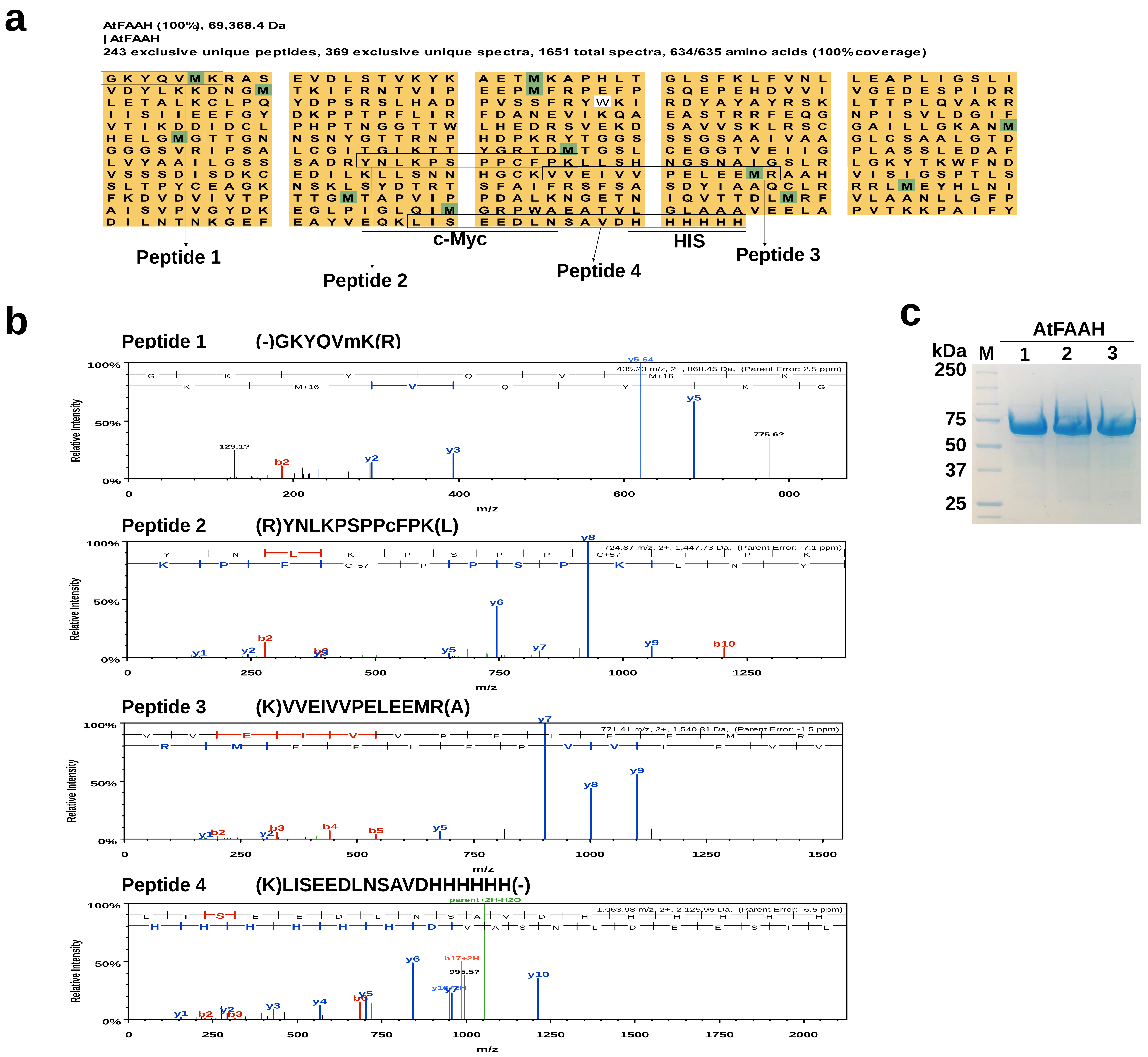

Figure S16. Liquid chromatography with tandem mass spectrometry (LC/MS/MS) of AtFAAH. (a) Amino acid sequence of AtFAAH. Identified sequences after digestion are highlighted with the c-Myc and HIS tags labelled. Four regions were selected (boxed) for spectra analysis. (b) Corresponding spectra of the regions selected in panel (a). (c) Coomassie stained SDS-PAGE gel of AtFAAH samples purified by a Superdex 200 increase 10/300 GL column. Protein marker was loaded in lane M and identical samples were loaded in lanes 1, 2, and 3. Samples were cut out and analyzed by LC/MS/MS. The uncropped/unedited SDS-PAGE gels used in Supplementary Fig. 16 (panel c) can be found in Supplementary Fig. S26.

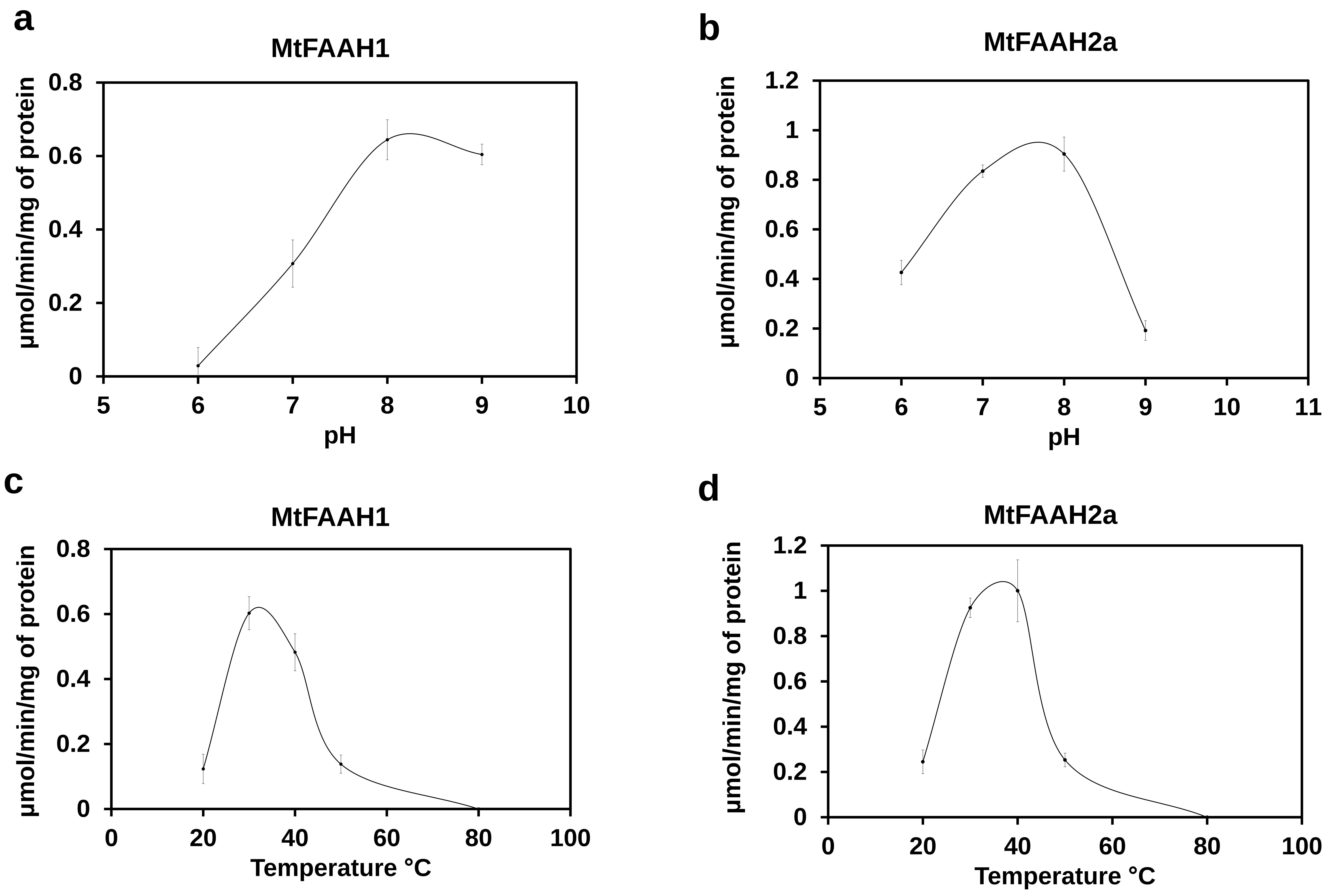

Figure S17. Activity of MtFAAH1 and MtFAAH2a at different pH and temperature conditions. (a) MtFAAH1 or (b) MtFAAH2a were incubated with NAE12:0 in reaction buffers at three different pH values, 6.0, 7.0, 8.0 and 9.0. (c) MtFAAH1 or (d) MtFAAH2a were incubated with NAE12:0 at five different temperatures (20, 30, 40, 50, or 80 °C). Activity is reported as µmol of ethanolamide produced per unit of time (min) per amount of protein used (mg). Data represent means ± S.D. of triplicate assays.

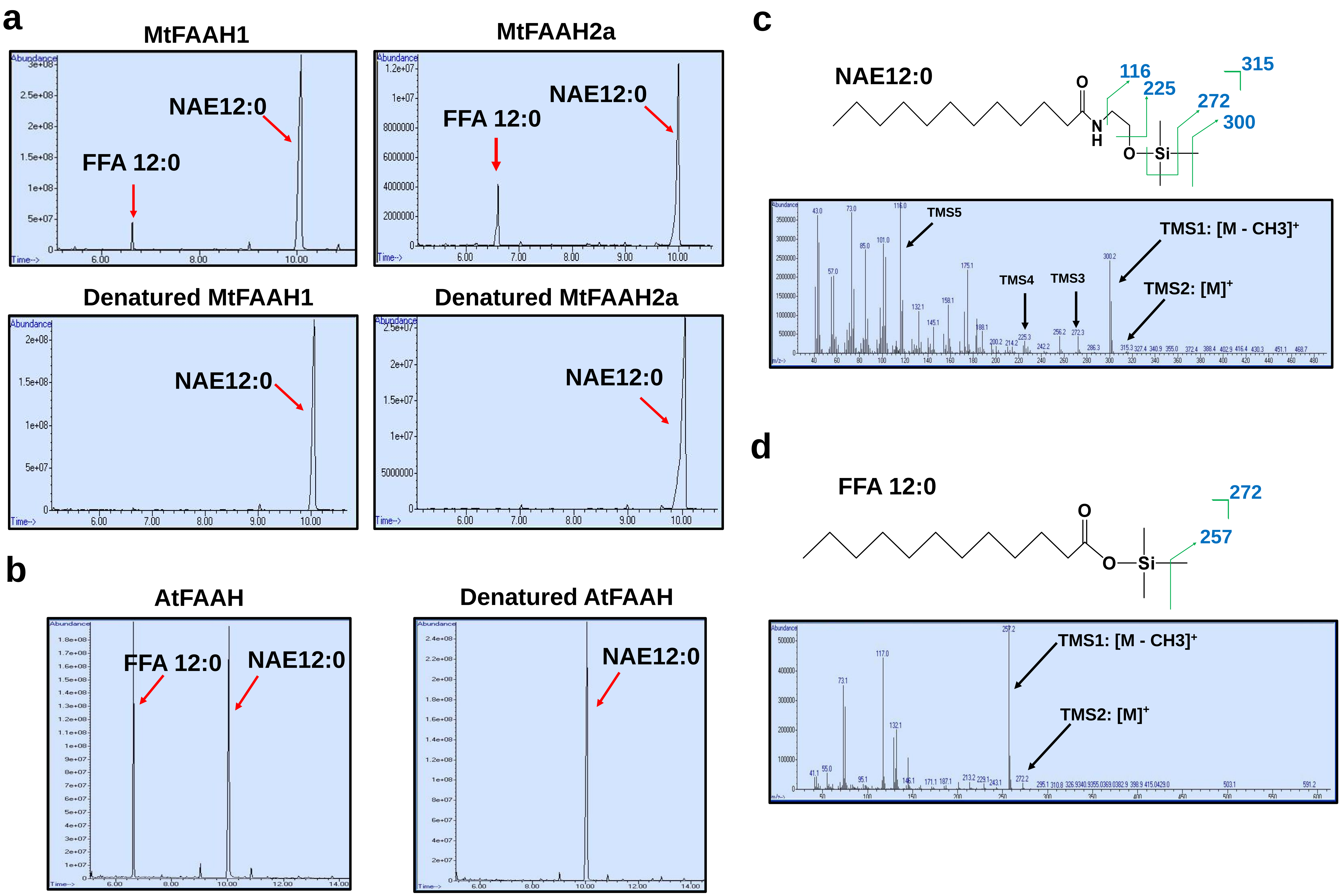

Figure S18. GCMS activity assays for MtFAAH1, MtFAAH2a, or AtFAAH towards NAE12:0. (a) Representative chromatograms in full scan mode show NAE12:0 and its corresponding FFA 12:0 product at retention times of ≈9.6 and ≈6.6 min, respectively. Reactions with denatured MtFAAHs were used as negative controls. MtFAAHs were denatured by boiling MtFAAHs at 80-100 °C for 15-30 minutes. (b) Reaction with AtFAAH was included in the experiments, as positive control. Reaction with denatured AtFAAH was used as negative control. Chemical structures and representative mass spectra plots for trimethylsilyl (TMS)-derivatives of (c) NAE12:0 or (d) FFA 12:0. Arrows point at the different diagnostic TMS ions in each structure.

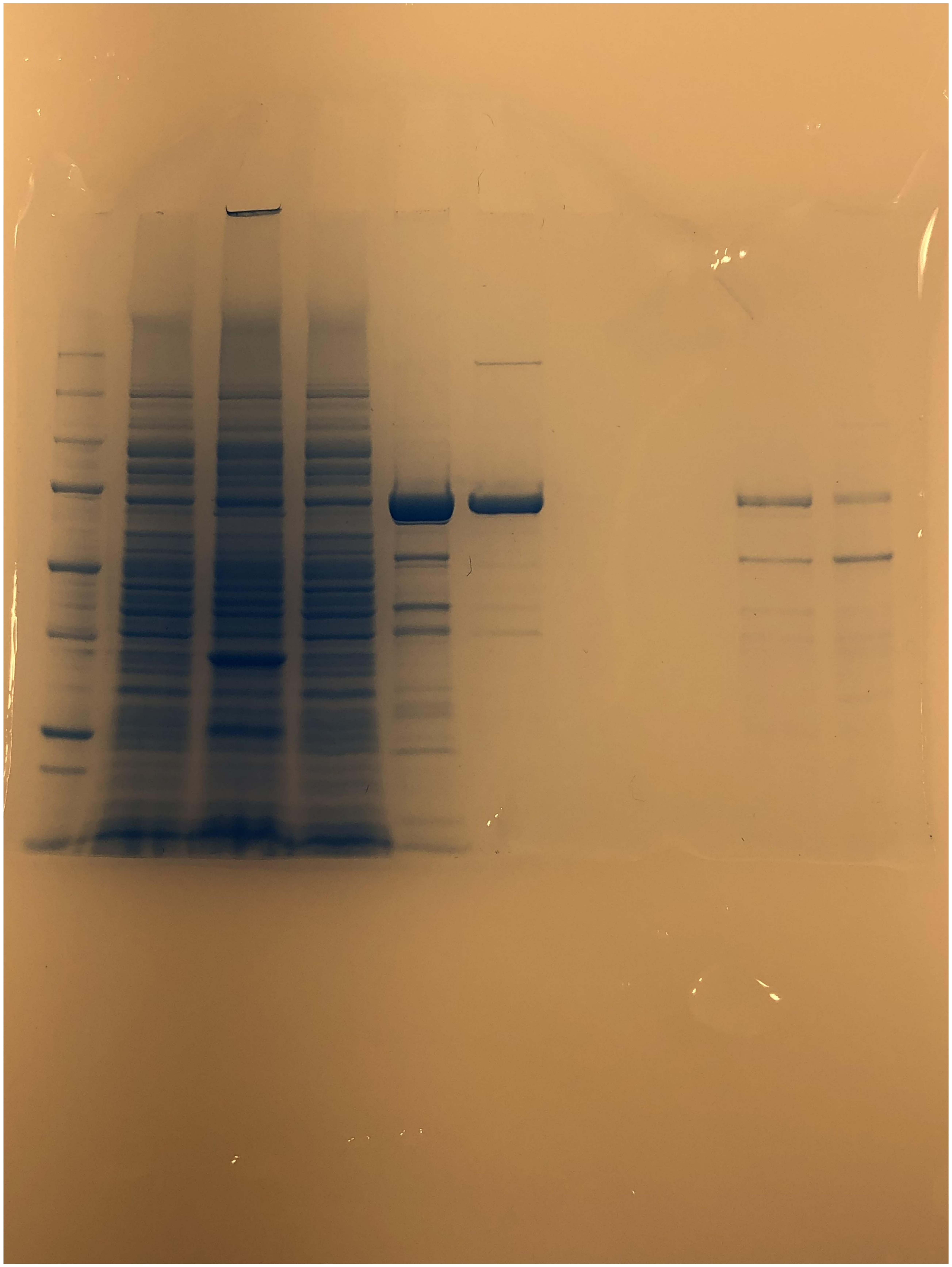

Figure S19. Uncropped/unedited SDS-PAGE gel used in Figure 6b.

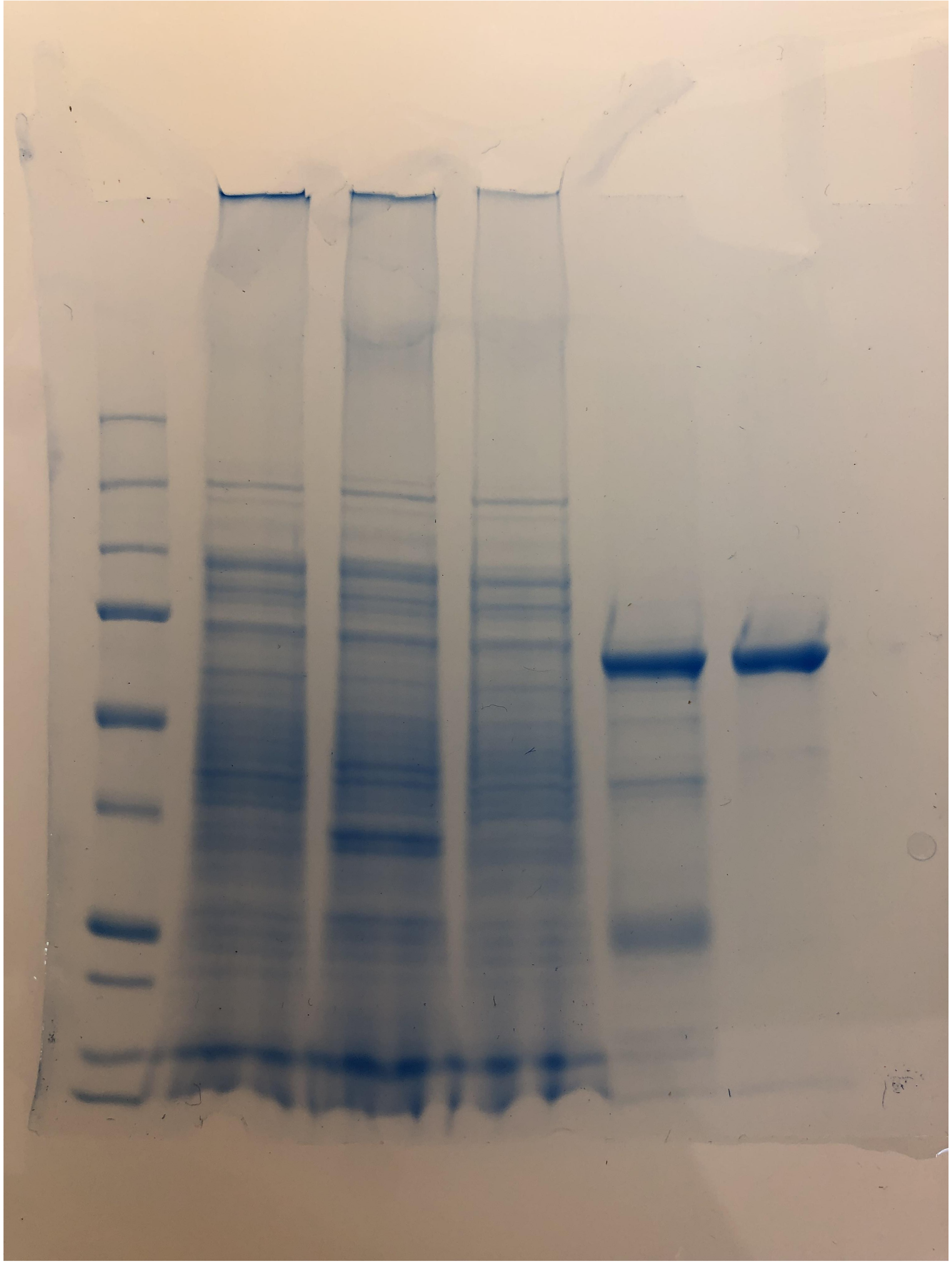

Figure S20. Uncropped/unedited SDS-PAGE gel used in Figure 6d.

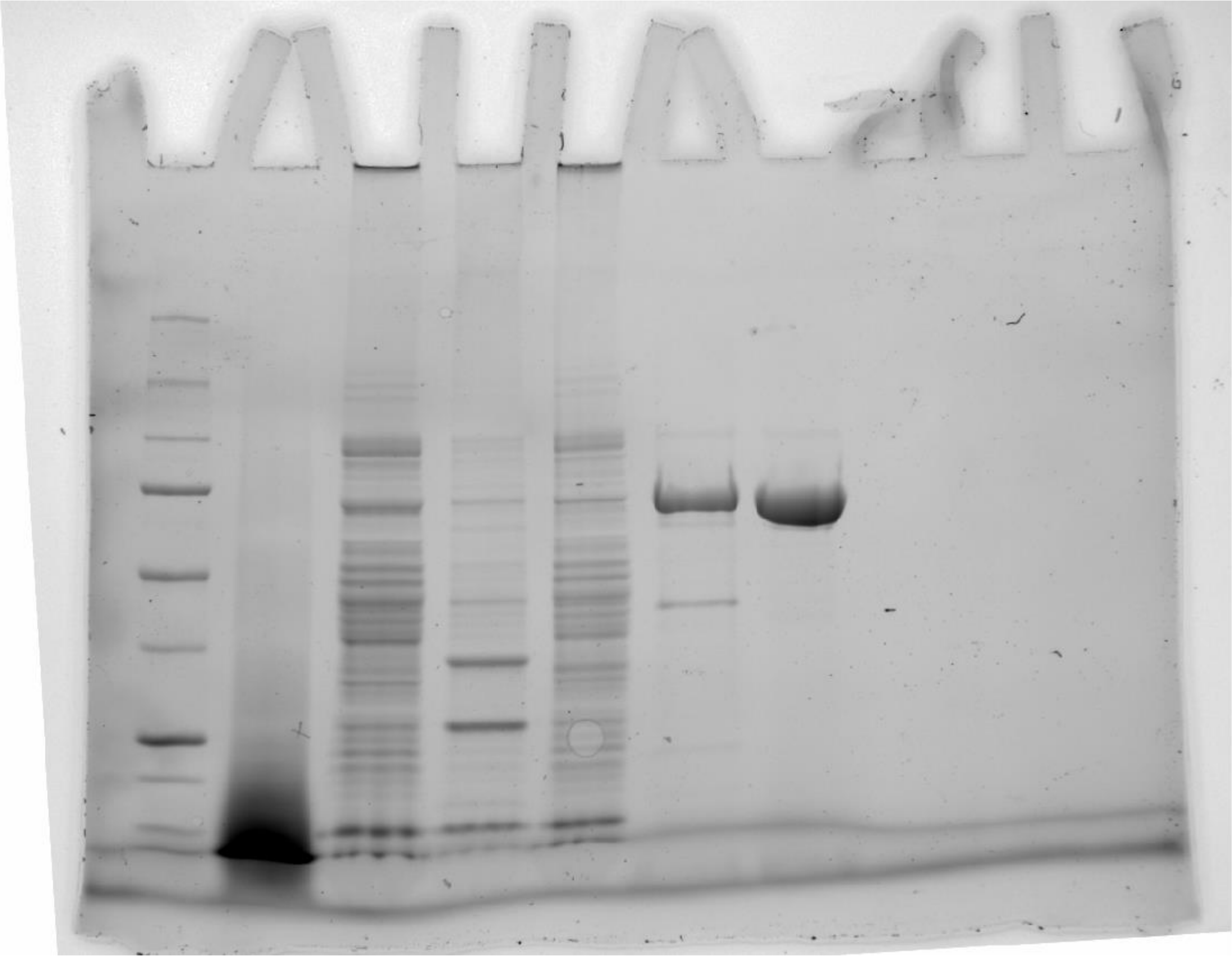

Figure S21. Uncropped/unedited SDS-PAGE gel used in Supplementary Figure S13 (panel c), corresponds to AtFAAH protein purification.

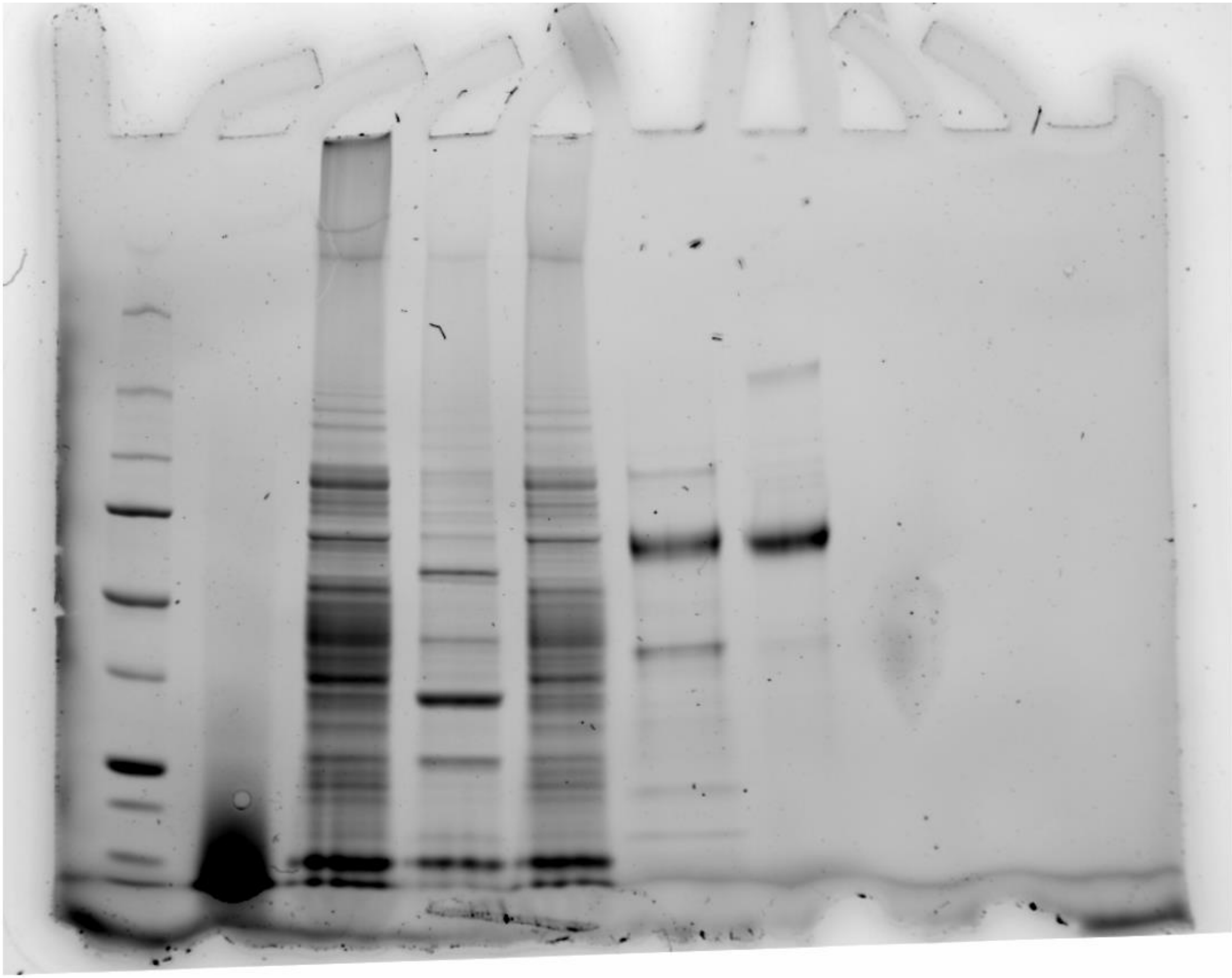

Figure S22. Uncropped/unedited SDS-PAGE gel used in Supplementary Figure S13 (panel c), corresponds to MtFAAH1 protein purification.

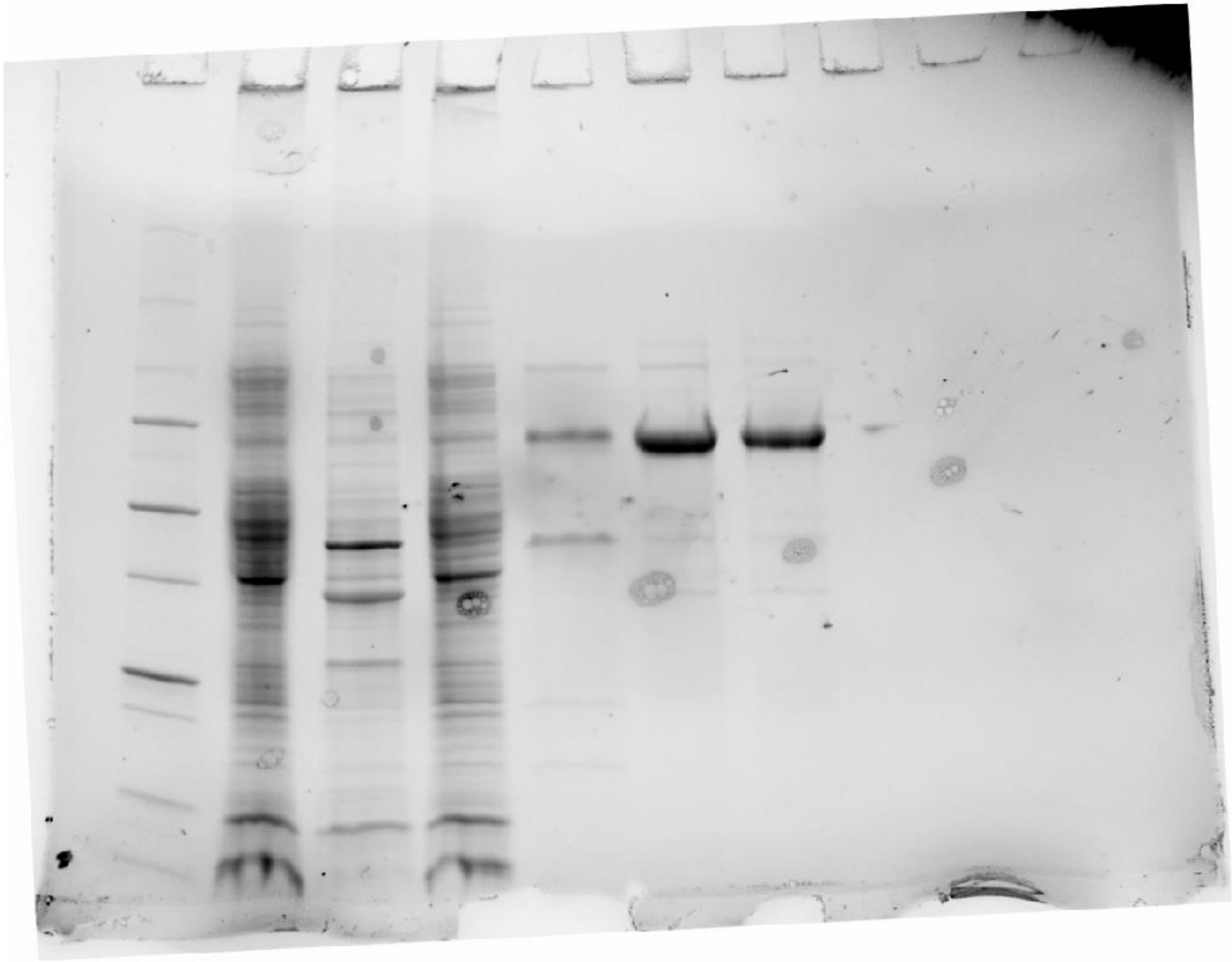

Figure S23. Uncropped/unedited SDS-PAGE gel used in Supplementary Figure S13 (panel c), corresponds to MtFAAH2a protein purification.

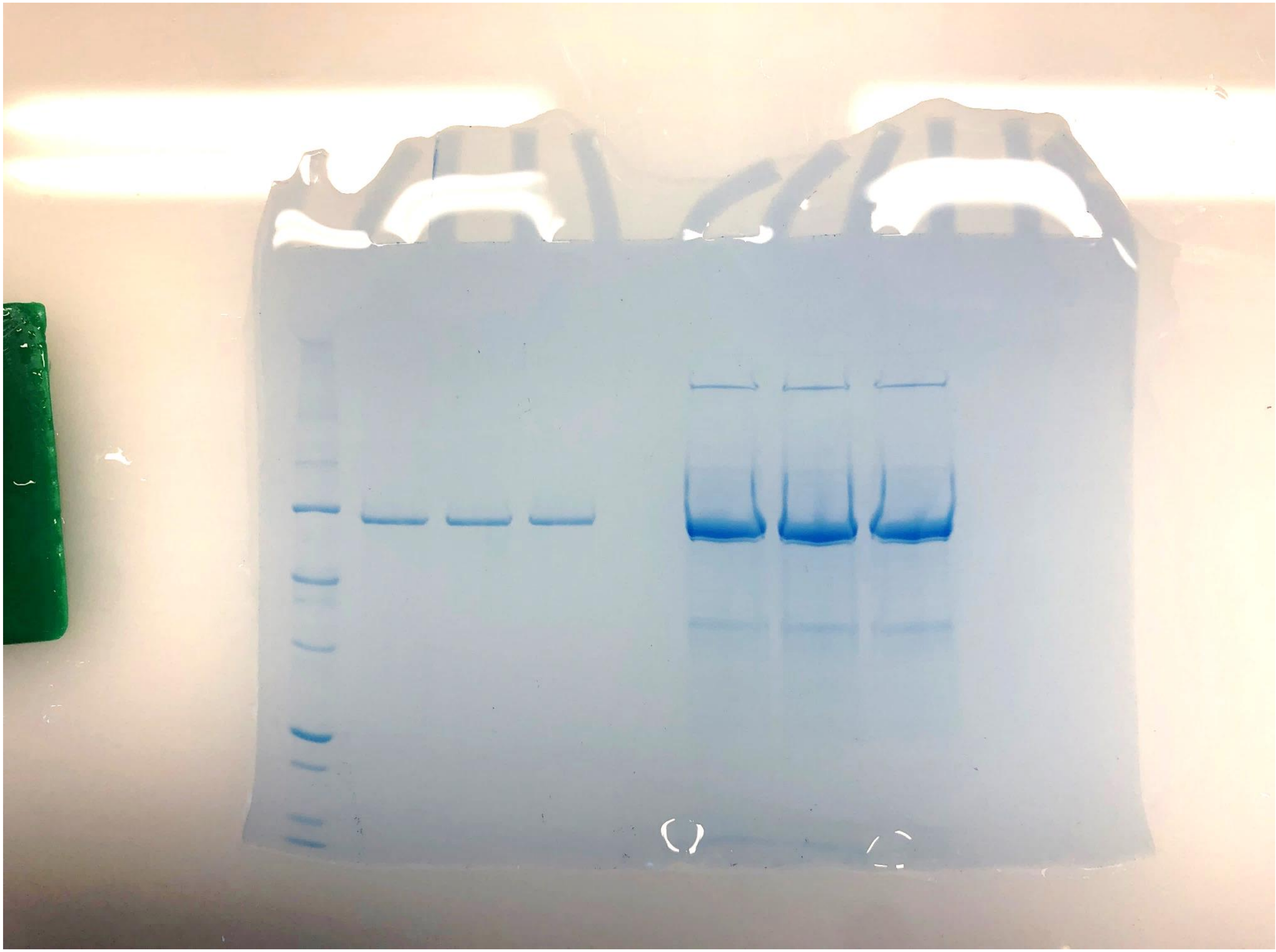

Figure S24. Uncropped/unedited SDS-PAGE gel used in Supplementary Figure S14, corresponding to AtFAAH protein purification.

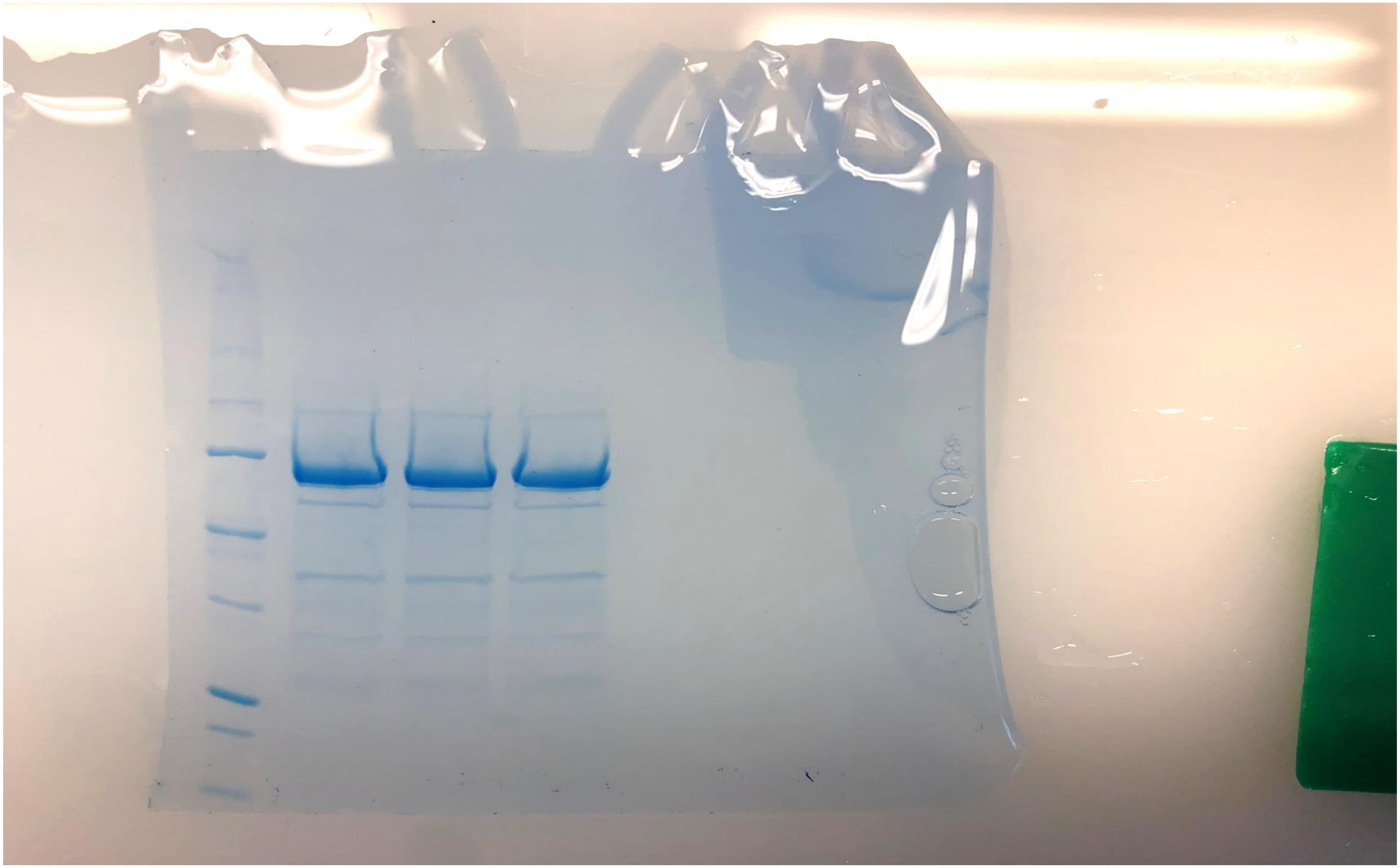

Figure S25. Uncropped/unedited SDS-PAGE gel used in Supplementary Figure S15, corresponding to MtFAAH1 protein purification.

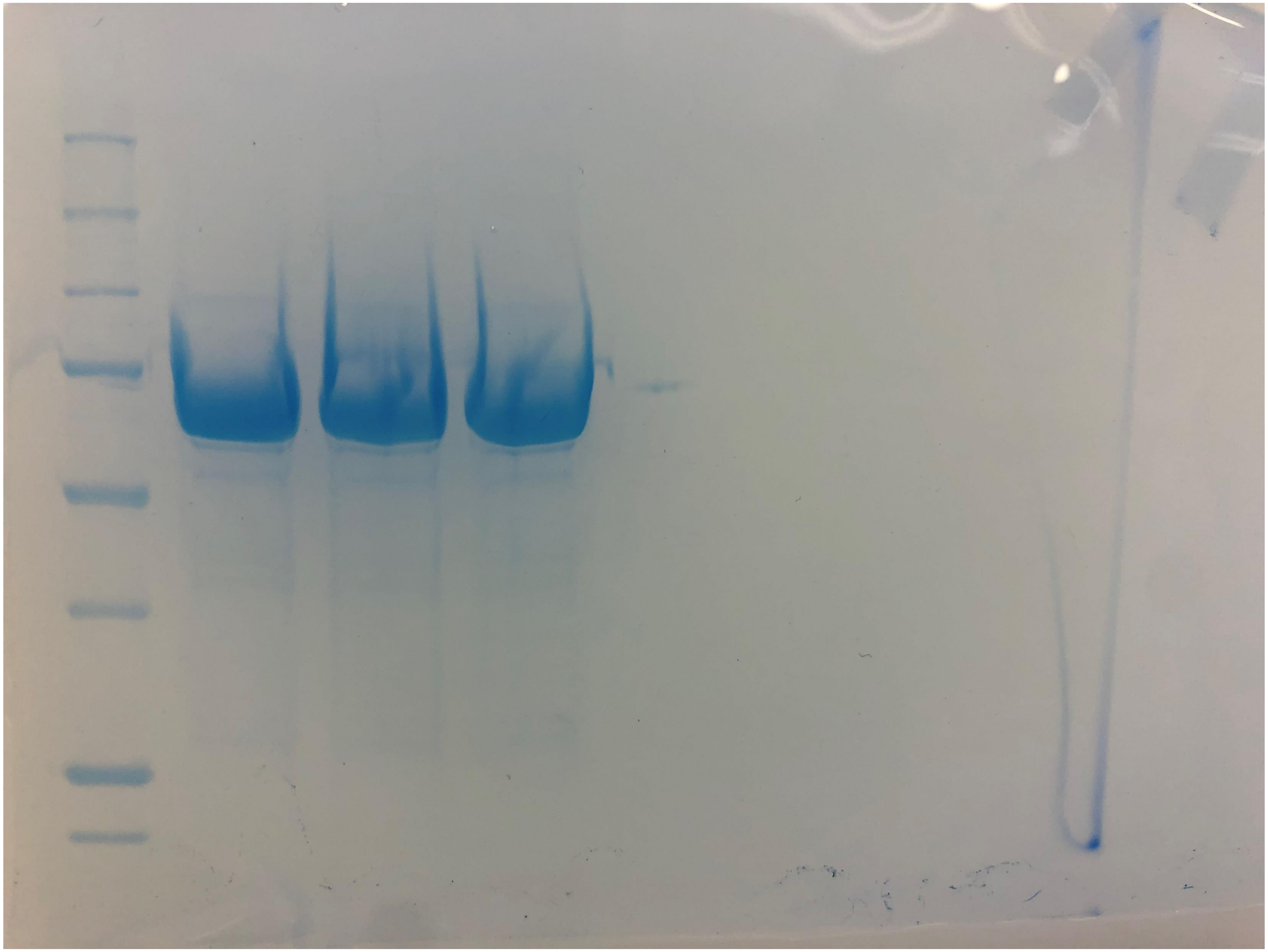

Figure S26. Uncropped/unedited SDS-PAGE gel used in Supplementary Figure S16, corresponding to MtFAAH2a protein purification.

Table S1. Aminoacid FAAH sequences of selected plant species. FAAHs of ten different legumes are compiled in the table. *A. thaliana* FAAH sequence was included in the analysis as FAAH1 control. Table contains the scientific name, predicted FAAH group category, and NCBI accession for each sequence.

Plant species	FAAH group	NCBI amino acid accessions
Arabidopsis thaliana	FAAH1	NP_201249.2
Medicago truncatula	FAAH1	XP_003593320.2
Medicago truncatula	FAAH2a	XP_003630360.1
Medicago truncatula	FAAH2b	XP_003602690.2
Glycine max	FAAH1	XP_014621333.1
Glycine max	FAAH2	XP_003528069.1
Pisum sativum	FAAH1	KAI5448288.1
Pisum sativum	FAAH2a	KAI5384119.1
Pisum sativum	FAAH2b	KAI5404910.1
Phaseolus vulgaris	FAAH1	XP_007148324.1
Phaseolus vulgaris	FAAH2a	XP_007159883.1
Phaseolus vulgaris	FAAH2b	XP_007137744.1
Arachis hypogaea	FAAH1	XP_025681944.1
Arachis hypogaea	FAAH2a	XP_025637667.1
Arachis hypogaea	FAAH2b	XP_025673234.1
Vigna radiata	FAAH1	XP_014518779.1
Vigna radiata	FAAH2	XP_014507133.1
Cicer arietinum	FAAH1	XP_004485635.1
Cicer arietinum	FAAH2	XP_004503894.1
Arachis duranensis	FAAH1	XP_015943396.1
Arachis duranensis	FAAH2a	XP_015955807.1
Arachis duranensis	FAAH2b	XP_015937468.1
Glycine soja	FAAH1	XP_028204397.1
Glycine soja	FAAH2a	KHN16930.1
Glycine soja	FAAH2b	KHN09653.1

Table S2. Pairwise similarity matrix for AtFAAH, and MtFAAHs. Data derived from Clustal Omega alignments.

% Similarity	AtFAAH	MtFAAH1	MtFAAH2a	MtFAAH2b
**AtFAAH**	100	65.84	43.83	43.65
**MtFAAH1**	65.84	100	42.43	43.07
**MtFAAH2a**	43.83	42.43	100	77.59
**MtFAAH2b**	43.65	43.07	77.59	100

Table S3. SWISS-MODEL parameters derived from the homology models created for MtFAAH1 and MtFAAH2a.

Model	Template	GMQE	QMEANDisCo Global	QSQE	Seq Identity (%)	Oligo-state	Found by	Method	Resolution	Seq Similarity	Range	Coverage	Description
MtFAAH1	6dhv.1.A	0.90	0.88 ± 0.05	0.69	65.79	homo-dimer	HHblits	X-ray	2.10Å	0.50	4 - 605	1	Fatty acid amide hydrolase
MtFAAH2a	6dhv.1.A	0.80	0.79 ± 0.05	0.51	43.79	homo-dimer	HHblits	X-ray	2.10Å	0.41	8 - 607	0.98	Fatty acid amide hydrolase

Table S4. General, glycine, proline and pre-proline Ramachandran scores corresponding to homology models for MtFAAH1 and MtFAAH2a. The MolProbity tool build within SWISS-MODEL was utilized for calculation of all scores.

Model	MolProbity Results		
MtFAAH1	MolProbity Score	1.1	
	Clash Score	0.76	(A275 ARG-A561 GLU)
	Ramachandran Favoured	95.59%	
	Ramachandran Outliers	0.67%	B24 ILE, A458 ASP, A333 SER, A604 LEU, B380 ASP, B9 PRO, A9 PRO, B604 LEU
	Rotamer Outliers	1.17%	A333 SER, B90 VAL, B411 GLU, A411 GLU, A382 ASP, B333 SER, B380 ASP, A7 MET, A368 LYS, B382 ASP, A416 LEU, B1 MET
	C-Beta Deviations	7	B379 SER, A333 SER, A604 LEU, A384 ASP, A579 ILE, A383 THR, B333 SER
	Bad Bonds	0 / 9443	
	Bad Angles	86 / 12834	A458 ASP, B380 ASP, (B542 VAL-B543 PRO), A244 PHE, (A542 VAL-A543 PRO), (B381 ASP-B382 ASP), (B531 MET-B532 PRO), B244 PHE, (B307 ILE-B308 PRO), (A380 ASP-A381 ASP), (A531 MET-A532 PRO), (A520 PRO-A521 PRO), (B453 ASN-B454 PRO), (A453 ASN-A454 PRO), (A382 ASP-A383 THR), (B520 PRO-B521 PRO), B95 ASP, (A307 ILE-A308 PRO), B29 HIS, A95 ASP, A604 LEU, B12 ASP, (A73 GLU-A74 PRO), A12 ASP, A455 ASP, (B73 GLU-B74 PRO), B458 ASP, (A378 SER-A379 SER), (A509 THR-A510 PRO), (B509 THR-B510 PRO), A29 HIS, (B283 GLY-B284 PRO), (A283 GLY-A284 PRO), B87 HIS, (B379 SER-B380 ASP), A222 HIS, B495 HIS, B210 HIS, B604 LEU, A495 HIS, (A519 ILE-A520 PRO), B251 HIS, (B519 ILE-B520 PRO), B212 HIS, B545 ASN, A251 HIS, B222 HIS, B440 HIS, A212 HIS, A205 ASP, A210 HIS, A402 HIS, A545 ASN, B175 GLU, B402 HIS, A440 HIS, B132 ASP, A421 HIS, (B603 VAL-B604 LEU), B187 PHE, B271 HIS, B421 HIS, (A117 LEU-A118 TRP), (A77 LYS-A78 PRO), A271 HIS, B602 ASP, (A143 THR-A144 PRO), B253 PHE, (B143 THR-B144 PRO), (B59 ASN-B60 LYS), A253 PHE, (B210 HIS-B211 PRO), B455 ASP, (B395 THR-B396 PRO), (B212 HIS-B213 PRO), B505 ASP, A111 HIS, B592 GLU, B33 PHE, A200 PHE, B70 PHE, (A59 ASN-A60 LYS), (B77 LYS-B78 PRO), B111 HIS
	Cis Non-Proline	8 / 1119	(A117 LEU-A118 TRP), (A279 GLY-A280 SER), (A380 ASP-A381 ASP), (A382 ASP-A383 THR), (B279 GLY-B280 SER), (B378 SER-B379 SER), (B380 ASP-B381 ASP), (B381 ASP-B382 ASP)
	Cis Prolines	4 / 86	(A163 PRO-A164 PRO), (A210 HIS-A211 PRO), (B163 PRO-B164 PRO), (B210 HIS-B211 PRO)
	Twisted Prolines	2 / 86	(A8 VAL-A9 PRO), (B8 VAL-B9 PRO)
MtFAAH2	MolProbity Score	1.72	
	Clash Score	6.01	(B426 TRP-B584 LEU), (A426 TRP-A584 LEU), (A390 ILE-A583 HIS), (B508 ILE-B584 LEU), (B12 LYS-B435 GLU), (A508 ILE-A584 LEU), (A420 LEU-A426 TRP)
	Ramachandran Favoured	94.31%	
	Ramachandran Outliers	1.34%	A338 PRO, B338 PRO, A168 PRO, B168 PRO, B460 ASP, B130 PRO, B340 ASN, B606 LEU, A123 ARG, A340 ASN, A13 PRO, B13 PRO, B366 GLU, A366 GLU, A405 CYS, B405 CYS
	Rotamer Outliers	0.99%	B438 VAL, A438 VAL, A42 VAL, B593 LEU, B236 ASP, A12 LYS, B67 LEU, A67 LEU, A400 LYS, B157 ARG
	C-Beta Deviations	8	A405 CYS, B405 CYS, B384 LEU, A101 ASP, A606 LEU, B375 ILE, A329 SER, B391 SER
	Bad Bonds	6 / 9486	B384 LEU-B385 PRO, A426 TRP, B384 LEU, B385 PRO, A583 HIS, B426 TRP
	Bad Angles	92 / 12904	(B384 LEU-B385 PRO), B385 PRO, B384 LEU, A583 HIS, B507 ASP, A182 ASP, (B167 LYS-B168 PRO), (A384 LEU-A385 PRO), A507 ASP, (A117 LEU-A118 PRO), A212 ASP, (A167 LYS-A168 PRO), (B106 PRO-B107 PRO), (A106 PRO-A107 PRO), (B314 MET-B315 PRO), (A592 CYS-A593 LEU), B16 ASP, B212 ASP, B333 HIS, (A314 MET-A315 PRO), (B123 ARG-B124 PRO), (B365 GLY-B366 GLU), (A123 ARG-A124 PRO), A14 VAL, B14 VAL, (A365 GLY-A366 GLU), (A511 SER-A512 PRO), (B511 SER-B512 PRO), A460 ASP, B258 HIS, A258 HIS, B426 TRP, A17 VAL, A333 HIS, B152 HIS, B175 PHE, A92 HIS, A175 PHE, (B337 ILE-B338 PRO), A188 ASN, B105 THR, A152 HIS, A483 MET, A12 LYS, (B170 LEU-B171 GLN), B270 HIS, A120 THR, A278 HIS, B92 HIS, A229 HIS, A547 ASN, B229 HIS, A420 LEU, (A337 ILE-A338 PRO), B443 HIS, B423 HIS, B547 ASN, A443 HIS, (A84 VAL-A85 PRO), A132 PHE, (B84 VAL-B85 PRO), A231 GLN, B278 HIS, B462 ASN, (A371 GLN-A372 PRO), A423 HIS, (B519 TYR-B520 PRO), B342 THR, (A519 TYR-A520 PRO), B69 HIS, (B150 THR-B151 PRO), A270 HIS, A69 HIS, (B371 GLN-B372 PRO), (A150 THR-A151 PRO), A342 THR, B12 LYS, B584 LEU
	Cis Non-Proline	6 / 1122	(A170 LEU-A171 GLN), (A286 GLY-A287 SER), (A592 CYS-A593 LEU), (B93 LYS-B94 GLU), (B170 LEU-B171 GLN), (B286 GLY-B287 SER)
	Cis Prolines	3 / 76	(A217 LEU-A218 PRO), (B217 LEU-B218 PRO), (B384 LEU-B385 PRO)

Table S5. List of residues that are predicted to be part of the substrate binding pocket (SBPs) of AtFAAH, MtFAAH1, or MtFAAH2a.

	Predicted substrate binding pocket (SBP)		
**Number**	**AtFAAH**	**MtFAAH1**	**MtFAAH2a**
1	M25	V25	V32
2	A27	A27	A34
3	L55	L55	L62
4	N59	N59	N66
5	M61	I61	I68
6	K205	K204	K211
7	D207	D206	E213
8	G255	G254	G261
9	M256	M255	A262
10	G257	G256	G263
11	T258	T257	T264
12	S281	S280	S287
13	T300	T299	V306
14	S305	S304	S311
15	C332	C331	P338
16	G335	G334	W341
17	E338	E337	G344
18	H441	H440	H443
19	V442	L441	Y444
20	I445	I444	I447
21	S472	S471	A473
22	I475	L474	I476
23	F476	F475	Y477
24	F479	F478	F480
25	I532	M531	Y533
26	T535	T534	G536
27	T536	G535	A537
28	M539	M538	I540

Table S6. List of residues predicted to be part of helices regions expected to interact with acyl or aryl tails of ligands in the substrate binding pockets of plant FAAHs. *Arabidopsis thaliana* (AtFAAH) was used to predict corresponding residues in the helices in *Medicago truncatula* (MtFAAHs).

Helix region			
**Number**	**AtFAAH**	**MtFAAH1**	**MtFAAH2a**
1	Asn531	Asp530	Asp532
2	Ile532	Met531	Tyr533
3	Gln533	Pro532	Val534
4	Val534	Thr533	Asn535
5	Thr535	Thr534	Gly536
6	Thr536	Gly535	Ala537
7	Asp537	Tyr536	Ala538

Table S7. Purification table of recombinant MtFAAH1 and MtFAAH2a. Standard deviation represent the mean of three technical replicates. Data represent means ± S.D. of triplicate assays with cell lysate, IMAC or SEC purified fractions. NAE12:0 (100 μM) was used as substrate for the reactions. Abbreviations: Enzyme unit (U) represents the μmol of product (ethanolamine) generated per unit of time (min)

FAAH	Purification Step	Volume (mL)	Total Protein (mg)	Total activity (U)	Specific activity (U/mg)	Yield (%)	Purification (fold)
MtFAAH1	Crude Cell Lysate	10	160	$1.0 \times 10^{-3} \pm 4.1 \times 10^{-5}$	$6.3 \times 10^{-6} \pm 2.6 \times 10^{-7}$	100	1
	IMAC Purified	10	0.080	$2.6 \times 10^{-4} \pm 9.8 \times 10^{-5}$	$3.3 \times 10^{-3} \pm 1.2 \times 10^{-3}$	26	521
	SEC Purified	2	0.020	$1.5 \times 10^{-4} \pm 2.8 \times 10^{-5}$	$7.5 \times 10^{-3} \pm 1.4 \times 10^{-3}$	15	1162
MtFAAH2a	Crude Cell Lysate	10	220	$1.3 \times 10^{-3} \pm 3.1 \times 10^{-5}$	$6.1 \times 10^{-6} \pm 1.4 \times 10^{-7}$	100	1
	IMAC Purified	10	0.120	$3.5 \times 10^{-4} \pm 4.0 \times 10^{-5}$	$2.9 \times 10^{-3} \pm 3.3 \times 10^{-4}$	26	480
	SEC Purified	2	0.020	$4.0 \times 10^{-4} \pm 5.2 \times 10^{-5}$	$2.0 \times 10^{-2} \pm 2.6 \times 10^{-3}$	30	3263

Table S8. Primers used in this study. Oligo name, sequence, purpose, target, annealing temperature (Tm), and predicted size (base pairs-bp) are indicated in the table.

Oligo name	Oligo sequence (5' to 3')	Purpose	Target	Tm (°C)	Size (bp)
pTrcHis_FWD	GAGGTATATATTAATGTATCG	Sequencing	Plasmids pTrcHis2 TOPO	51	N/A
pTrcHis_REV	GATTTAATCTGTATCAGG	Sequencing	Plasmids pTrcHis2 TOPO	50	
Nest_MtFA1_FWD	ACACCTCTCTTTCTACTTCTACGTTTG	PCR1	MtFAAH1	60	2067
Nest_MtFA1_REV	TGCCAAACCGTAAATCCTGT	PCR1	MtFAAH1	60	
MtFAAH1_FWD	ATGGGGAAGAAGCGTGTAATGGTGCCG	Cloning/Sequencing/ PCR2	MtFAAH1	75	1821
MtFAAH1_REV	GTTAGCCCCCAGAACATCGTAGTATGTCAC	Cloning/Sequencing/PCR2	MtFAAH1	72	
FW_RT_faah1	TGGTGGATCCTCTTCAGGTC	Sequencing	MtFAAH1	60	439
REV_RT_faah1	ACAACCATGTGCCTTTGACA	Sequencing	MtFAAH1	60	
MedFAAH2a_FW	ATGGGTTTGTTCAAAAGTAAACGTGTG	Cloning/Sequencing	MtFAAH2a	59	1827
MedFAAH2a_REV	ATTTGTTCTGAGCAGATCATAAAAAATTG	Cloning/Sequencing	MtFAAH2a	58	
MedFAAH2a_FWD1	ACCACACATGGTTGCAGAAA	Sequencing	MtFAAH2a	60	800
MedFAAH2a_REV1	GAGCACCGGAACAGCATATT	Sequencing	MtFAAH2a	60	

Supplementary Video S1. Top view representation of 100 ns molecular dynamic simulation (MDS) of MtFAAH1 (blue cartoon) bound to NAE18:2 (cyan spheres= acyl carbons; white spheres= hydrogen; red spheres= oxygen; blue spheres= nitrogen). Residues in the helix region (residues 530-536) are shown as yellow sticks. For clarity and visualization purposes, water, Na+ and Cl- ions have been removed from the system.

Supplementary Video S2. Bottom view representation of 100 ns molecular dynamic simulation (MDS) of MtFAAH1 (blue cartoon) bound to NAE18:2 (cyan spheres= acyl carbons; white spheres= hydrogen; red spheres= oxygen; blue spheres= nitrogen). Residues in the helix region (residues 530-536) are shown as yellow sticks. For clarity and visualization purposes, water, Na+ and Cl- ions have been removed from the system.

Supplementary Video S3. Representation of 100 ns molecular dynamic simulation (MDS) of the MAC of MtFAAH1 (blue VDW spheres) bound to NAE18:2 (yellow VDW spheres). For clarity and visualization purposes, water, Na+ and Cl- ions have been removed from the system.

Supplementary Video S4. Top view representation of 100 ns molecular dynamic simulation (MDS) of MtFAAH1 (blue cartoon) bound to *p*-coumaryl-HL (cyan sticks= acyl carbons; white sticks= hydrogen; red sticks= oxygen; blue sticks= nitrogen). Residues in the helix region (residues 530-536) are shown as yellow sticks. For clarity and visualization purposes, water, Na+ and Cl- ions have been removed from the system.

Supplementary Video S5. Bottom view representation of 100 ns molecular dynamic simulation (MDS) of MtFAAH1 (blue cartoon) bound to *p*-coumaryl-HL (cyan sticks= acyl carbons; white sticks= hydrogen; red sticks= oxygen; blue sticks= nitrogen). Residues in the helix region (residues 530-536) are shown as yellow sticks. For clarity and visualization purposes, water, Na+ and Cl- ions have been removed from the system.

Supplementary Video S6. Representation of 100 ns molecular dynamic simulation (MDS) of the MAC of MtFAAH1 (blue VDW spheres) bound to *p*-coumaryl-HL (red sticks). For clarity and visualization purposes, water, Na+ and Cl- ions have been removed from the system.

Supplementary Video S7. Top view representation of 100 ns molecular dynamic simulation (MDS) of MtFAAH2a (green cartoon) bound to NAE18:2 (cyan spheres= acyl carbons; white spheres= hydrogen; red spheres= oxygen; blue spheres= nitrogen). Residues in the helix region (residues 532-538) are shown as purple sticks. For clarity and visualization purposes, water, Na+ and Cl- ions have been removed from the system.

Supplementary Video S8. Bottom view representation of 100 ns molecular dynamic simulation (MDS) of MtFAAH2a (green cartoon) bound to NAE18:2 (cyan spheres= acyl carbons; white spheres= hydrogen; red spheres= oxygen; blue spheres= nitrogen). Residues in the helix region (residues 532-538) are shown as purple sticks. For clarity and visualization purposes, water, Na+ and Cl- ions have been removed from the system.

Supplementary Video S9. Representation of 100 ns molecular dynamic simulation (MDS) of the MAC of MtFAAH2a (green VDW spheres) bound to NAE18:2 (purple VDW spheres). For clarity and visualization purposes, water, Na+ and Cl- ions have been removed from the system.

Supplementary Video S10. Top view representation of 100 ns molecular dynamic simulation (MDS) of MtFAAH2a (green cartoon) bound to *p*-coumaryl-HL (cyan spheres= acyl carbons; white spheres= hydrogen; red spheres= oxygen; blue spheres= nitrogen). Residues in the helix region (residues 532-538) are shown as purple sticks. Tyr533 and Trp341 are represented as orange and blue sticks, respectively. For clarity and visualization purposes, water, Na+ and Cl- ions have been removed from the system.

Supplementary Video S11. Bottom view representation of 100 ns molecular dynamic simulation (MDS) of MtFAAH2a (green cartoon) bound to *p*-coumaryl-HL (cyan spheres= acyl carbons; white spheres= hydrogen; red spheres= oxygen; blue spheres= nitrogen). Residues in the helix region (residues 532-538) are shown as purple sticks. Tyr533 and Trp341 are represented as orange and blue sticks, respectively. For clarity and visualization purposes, water, Na+ and Cl- ions have been removed from the system.

Supplementary Video S12. Representation of 100 ns molecular dynamic simulation (MDS) of the MAC of MtFAAH2a (green VDW spheres) bound to *p*-coumaryl-HL (not visible due to closure of MAC). For clarity and visualization purposes, water, Na+ and Cl- ions have been removed from the system.
